# ERα/PR crosstalk is altered in the context of the ERα Y537S mutation and contributes to endocrine therapy-resistant tumor proliferation

**DOI:** 10.1038/s41523-023-00601-7

**Published:** 2023-11-30

**Authors:** Rosemary J. Huggins, Geoffrey L. Greene

**Affiliations:** Ben May Department for Cancer Research, University of Chicago, Chicago, IL USA

**Keywords:** Breast cancer, Transcription

## Abstract

The constitutively active *ESR1* Y537S mutation is associated with endocrine therapy (ET) resistance and progression of metastatic breast cancer through its effects on estrogen receptor (ERα) gene regulatory functions. However, the complex relationship between ERα and the progesterone receptor (PR), known as ERα/PR crosstalk, has yet to be characterized in the context of the ERα Y537S mutation. Using proximity ligation assays, we identify an increased physical interaction of ERα and PR in the context of the ERα Y537S mutation, including in the nucleus where this interaction may translate to altered gene expression. As such, more than 30 genes were differentially expressed in both patient tumor and cell line data (MCF7 and/or T47D cells) in the context of the ERα Y537S mutation compared to ERα WT. Of these, *IRS1* stood out as a gene of interest, and ERα and PR occupancy at chromatin binding sites along *IRS1* were uniquely altered in the context of ERα Y537S. Furthermore, siRNA knockdown of *IRS1* or treatment with the IRS1 inhibitor NT-157 had a significant anti-proliferative effect in ERα Y537S cell lines, implicating IRS1 as a potential therapeutic target for restoring treatment sensitivity to patients with breast cancers harboring ERα Y537S mutations.

## Introduction

The use of endocrine adjuvant therapy (ET) in hormone-sensitive estrogen receptor (ERα)-positive breast cancers has significantly improved outcomes and relapse-free survival^1^. Unfortunately, ~25% of patients who are treated with ET for 5 years develop somatic *ESR1* (estrogen receptor gene) point mutations that drive therapy resistance and contribute to the progression of metastatic breast cancer. ERα Y537S is one of the most frequently identified ERα mutations in patients, with this mutation appearing in ~30% of circulating tumor cells from blood samples and at least 20% of metastatic tumors^2–6^.

Notably, ERα Y537S is very rarely found in primary treatment-naïve tumors and is associated with tumor progression, suggesting that ET results in selective pressure toward more resistant and aggressive metastases^3^. Previous structural assessment in our lab demonstrated that ERα Y537S stabilizes the activating function-2 (AF-2) cleft of the ERα ligand binding domain (LBD) in the agonist-bound conformation, which facilitates constitutive activity of the LBD, even in the absence of estradiol^7^. Conversely, ERα Y537S interferes with the antagonist state of AF-2, resulting in reduced affinity of antagonists for the receptor and resistance to inhibition by selective estrogen receptor modulators and degraders (SERMs and SERDs)^7^. Further investigation into the effects of ERα Y537S on the transcription factor activity of ERα identified ~900 genes that were significantly induced in MCF7 and T47D ERα Y537S cell lines, including several genes that were uniquely bound by ERα Y537S compared to unmutated ERα WT^3^.

While gene expression changes associated specifically with mutant ERα have understandably been the main focus in terms of assessing the effects of ERα Y537S, there are alterations to progesterone receptor (PR)-mediated gene expression as well. Previous research in our lab and others has assessed ERα/PR crosstalk and found that, in ERα+/PR+ treatment-naïve cells, combined modulation of both receptors promoted tumor regression, and chromatin binding profiles indicated that PR alters ERα-associated gene expression in the ERα WT context^8–11^. However, the effect of ERα Y537S on ERα/PR crosstalk has not been thoroughly investigated. Given that liganded ERα regulates *PGR* (PR gene) transcription, it is highly likely that the constitutively active ERα Y537S mutation results in altered PR expression and activity^9,12–15^. In this study, we aimed to determine the effects of the ERα Y537S mutation on ERα/PR crosstalk and resulting transcriptional activities and to elucidate how this interaction leads to ET resistance in ERα-positive breast cancer. We identified a unique transcriptome associated with the ERα Y537S mutation at shared regulatory binding sites of ERα and PR, including near *IRS1*. Our results suggest that inhibition of insulin receptor substrate-1 (IRS1) may restore therapeutic sensitivity to patients with ET-resistant breast cancer.

## Results

### PR agonism contributes to increased ERα/PR proximity in the context of the ERα Y537S mutation

The ERα Y537S mutation is often found in treatment-resistant metastatic breast cancers, and thus it is of significant interest to fully characterize the phenotypic effects of the mutation as well as how it may be targeted. Experiments were carried out in MCF7 and T47D cells expressing either unmutated ERα (ERα WT), heterozygous ERα WT/Y537S (ERα Y537S-het), or homozygous ERα Y537S/Y537S (ERα Y537S-hom). Though patient tumors tend to harbor heterozygous ERα mutations^16^, assessing the mutation in isolation (as with ERα Y537S-hom) is critical to understanding the phenotypic effects of the mutation without the interference of the unmutated receptor.

Unless otherwise noted, all experiments were carried out in cells treated with either hormone-deprivation (HD, phenol-red free media containing charcoal-stripped FBS) or estradiol (E2)-deprivation with PR-stimulation (10 nM R5020). HD was included because ERα Y537S mutations arise during or after ET, resulting in E2-independent ERα activity^17–19^. Though post-menopausal patients generally have very low levels of circulating progesterone, pre-menopausal patients are still exposed to progesterone at varying levels^20^. Thus, including E2-deprivation with PR stimulation alongside HD is important for understanding the relationship of ERα with liganded or unliganded PR, and how that is altered in the context of the ERα Y537S mutation.

**Fig. 1:**
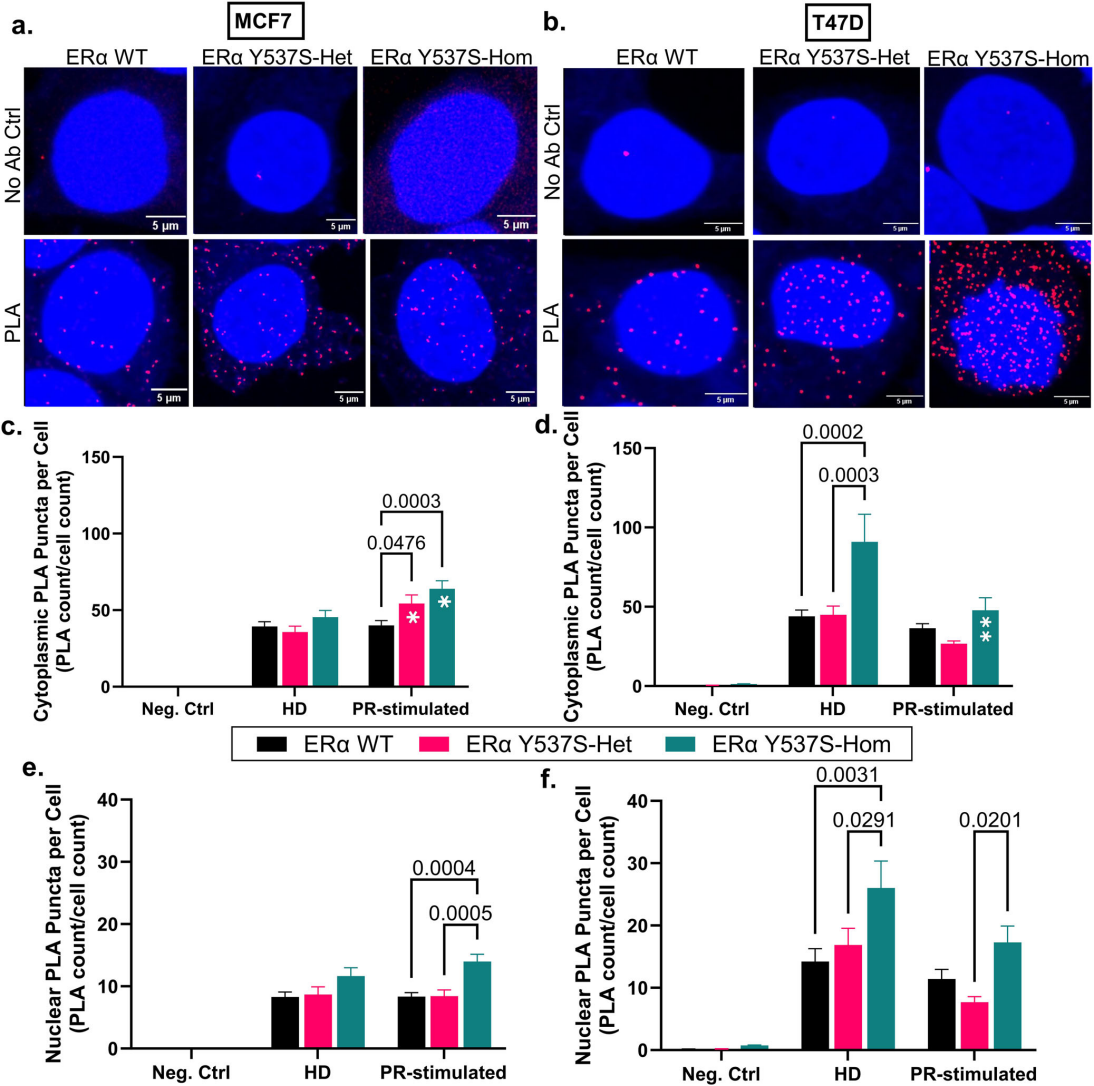
ERα/PR proximity-based interaction is increased in the context of ERα Y537S-hom relative to ERα WT or Y537S-het. Representative confocal images of PLA (red puncta) and DAPI (blue nuclei)-stained cells after vehicle treatment in **a** MCF7 and **b** T47D cells. Scale bars represent 5 um. Quantification of average cytoplasmic PLA puncta counts per cell for **c** MCF7 and **d** T47D cells. Quantification of average nuclear PLA puncta counts per cell for **e** MCF7 and **f** T47D cells. Data represents 3 replicates with error bars indicating standard error of the mean (SEM). *P*-values comparing cell variants are indicated. Asterisks within bars indicate statistically significant differences between HD and PR-stimulated treatments within a given cell variant (*p < 0.05, **p < 0.005).

Given the reported role of ERα/PR crosstalk in breast cancer progression, we first investigated the effect of ERα Y537S on the proximity-based interaction of the two receptors. Proximity ligation assays (PLA) against probed antibodies for ERα and PR identified puncta formation indicative of ERα/PR proximity-based interaction in the cytoplasm and nucleus of all cell variants, suggesting a role of ERα/PR interaction at chromatin as well as outside the nucleus (Fig. 1, Supplementary Figs. 1 and 2). Greater puncta formation per cell in MCF7 and T47D cells expressing ERα Y537S-hom indicates increased ERα/PR proximity compared to ERα WT or ERα Y537S-het cells (Fig. 1c–f). Treatment responsiveness concerning ERα/PR proximity was cell line-dependent; PLA puncta formation in MCF7 ERα Y537S-hom cells was induced in response to PR-stimulation with R5020 treatment (Fig. 1c, e) while their T47D counterparts were particularly sensitive to hormone-deprived (HD) conditions and (to a lesser extent) PR-stimulation (Fig. 1d, f). These results indicate that constitutively active ERα Y537S may contribute to increased ERα/PR physical interaction to a certain extent, and hormone-dependent PR activation drives even greater interaction of the two hormone receptors in the context of the ERα Y537S mutation. This is likely due to the fact that there are significantly higher levels of PR in ERα Y537S-hom cells compared to ERα WT or ERα Y537S-het cells (Supplementary Fig. 3), corresponding with increased ERα transcription factor activity driving PR expression^9,12–15^. With more PR present, ERα/PR physical interaction more readily occurs in the context of the homozygous ERα Y537S mutation.

### Homozygous expression of the ERα Y537S mutation results in a distinct transcriptome in MCF7 and T47D cell lines

RNA-seq was completed in MCF7 and T47D cell variants to assess transcriptomal changes associated with the ERα Y537S mutation. Two-hour treatment with 10 nM R5020 to stimulate PR was selected based on time course analysis of ERα WT transcription factor activity, as quantified by *SGK1* (an ERα and PR target gene) mRNA expression (Supplementary Fig. 4). Because ERα is constitutively active in the context of the ERα Y537S mutation, the optimal timepoint for transcriptome analysis was based on peak 17β-estradiol (E2)-induced activation of ERα WT (Supplementary Fig. 4a). To further validate the selection of two-hour PR stimulation as well as the quality of RNA for RNA-seq, we assessed *SGK1* expression in each ERα cell variant of MCF7 and T47D cells (Supplementary Fig. 4b, c). *SGK1* expression was largely increased in response to PR stimulation, across both cell lines and all variants (ERα WT, Y537S-het, and Y537S-hom). Notably, both MCF7 and T47D ERα Y537S-hom cells displayed elevated *SGK1* expression (relative to ERα WT) even in the absence of PR stimulation, highlighting the constitutive transcription factor activity of ERα Y537S (Supplementary Fig. 4b, c).

**Fig. 2:**
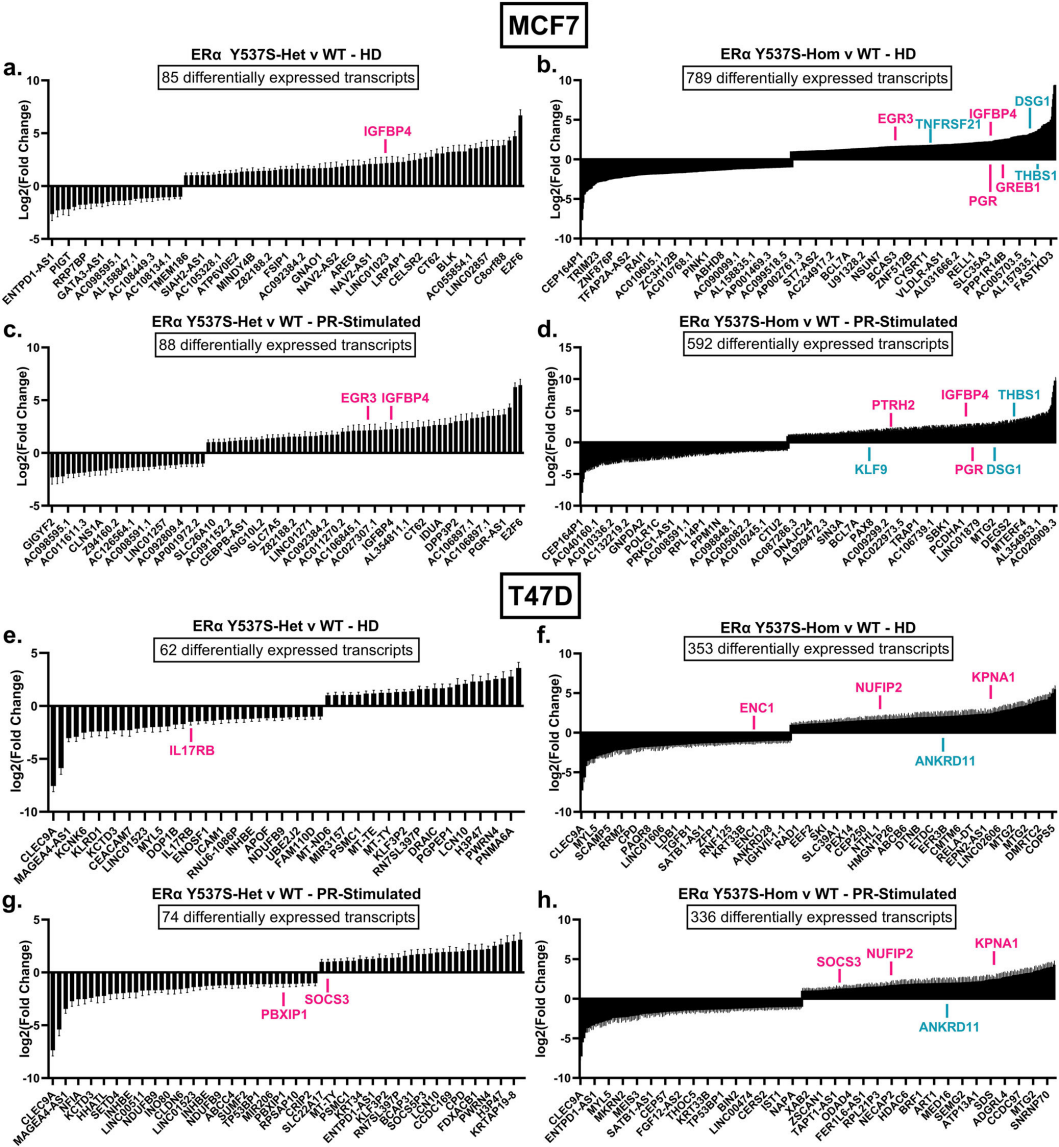
Cells expressing ERα Y537S-hom are transcriptomally unique from both ERα Y537S-het and ERα WT cells. Plots of log2(fold change) for differentially expressed transcripts (|log2(FC)|>1, p-adj. < 0.05) in **a**–**d** MCF7 cells and **e**–**h** T47D cells expressing ERα Y537S-het (**a**, **c**, **e**, **g**) or ERα Y537S-hom (**b**, **d**, **f**, **h**) relative to ERα WT, after HD (**a**, **b**, **e**, **f**) or PR-stimulated (**c**, **d**, **g**, **h**) treatment. Data represents 3 replicates with error bars indicating SEM. Differentially expressed ERα (pink) and PR (teal) target genes are noted above or below their corresponding bars.

Triplicate RNA-seq data clustered tightly for each cell line variant (ERα WT, Y537S-het, or Y537S-hom) and treatment (HD or PR-stimulated) (Supplementary Fig. 5). In both MCF7 and T47D cells and regardless of treatment, ERα Y537S-hom cells differentially expressed significantly more genes than ERα Y537S-het cells when each was compared to ERα WT (Fig. 2, gene expression data available through NCBI Gene Expression Omnibus, #GSE243454). Notably, this includes differential expression of numerous ERα and PR target genes in the context of ERα Y537S-hom under either HD or PR-stimulated conditions, highlighting a hormone-independent transcriptome in the context of the ERα Y537S mutation (Fig. 2, ERα and PR target genes noted in pink and teal, respectively). In total, over 600 genes and 350 genes were found to be differentially expressed in the context of the ERα Y537S mutation (heterozygous and homozygous, compared to ERα WT) in MCF7 and T47D, respectively (Fig. 2). These findings are in line with previous studies on the effect of the Y537S mutation on ERα-driven gene expression^3,16^.

We next filtered these data to include only genes containing shared cis-regulatory regions of ERα and PR binding identified by Khushi et al. This allowed us to focus on gene expression changes that might be a direct result of altered ERα/PR crosstalk, whereas previous research investigated transcriptomal changes correlated with ERα Y537S more generally^3,16,21,22^. The dataset from Khushi et al. was selected due to the stringent removal of biases using their previously published Binding Sites Analyser (BiSA) tool, leading to higher confidence in the resulting overlapping ERα/PR shared regulatory regions^23^. Similar to the pre-filtered data, MCF7 and T47D ERα Y537S-hom cells differentially expressed significantly more overlapping ERα/PR-shared regulatory genes than their respective ERα Y537S-het counterparts (Supplementary Table 3). These findings uncovered a distinct transcriptome associated with ERα Y537S in a context without clouding of data by the presence of ERα WT. However, without further analyses, these data are largely correlative and do not offer insight into the clinical significance or mechanism by which ERα Y537S alters ERα/PR-shared regulatory gene expression.

### Differentially expressed genes are conserved between MCF7, T47D, and patient tumors expressing ERα Y537S mutations

**Fig. 3:**
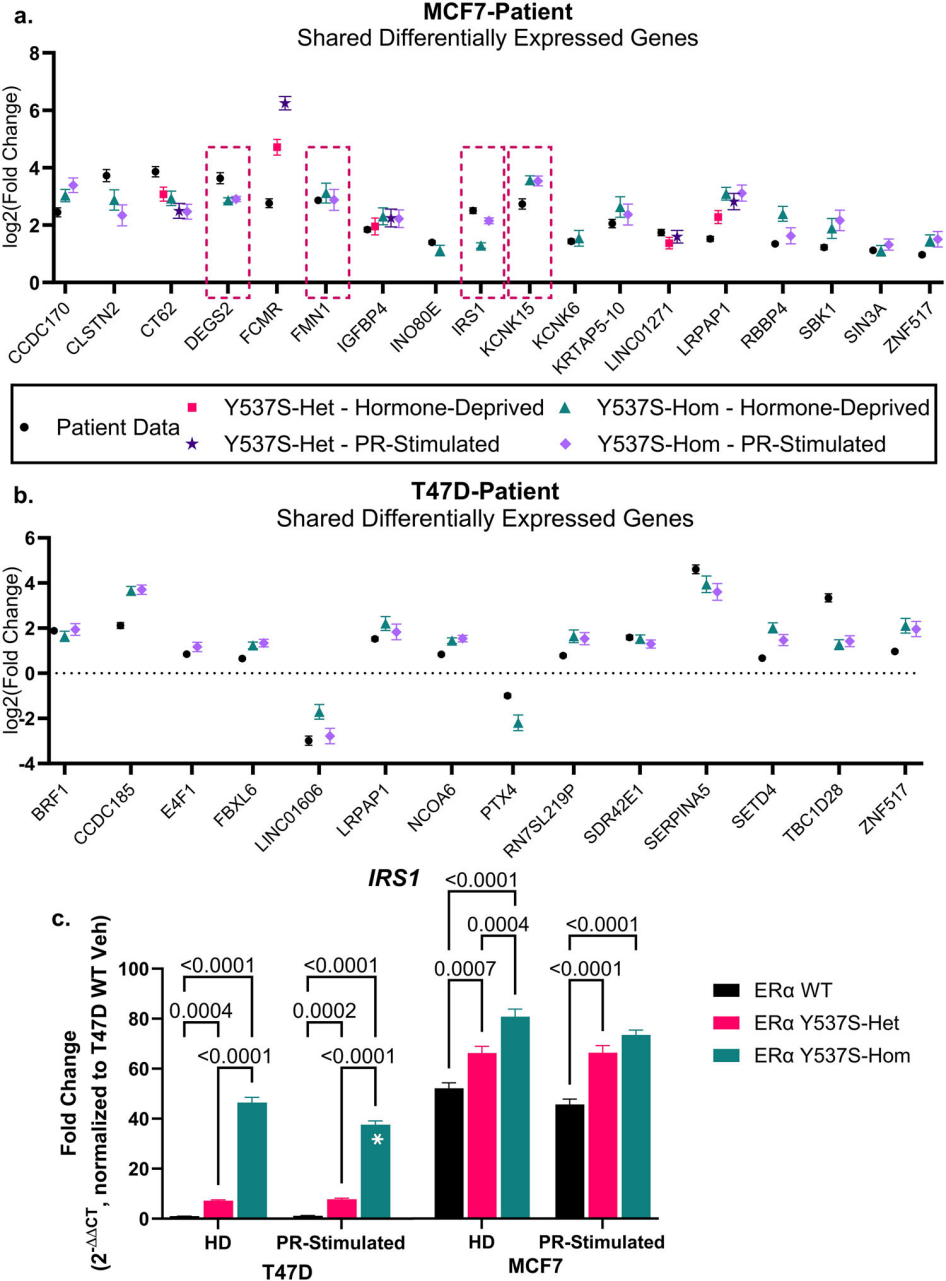
Patient breast cancers harboring ERα Y537S mutations share differential expression of several potential shared ERα/PR genes with immortalized cell lines. Log_2_(fold change) of differentially expressed genes shared between ERα Y537S-expressing patient tumor transcriptome data and **a** MCF7 and **b** T47D cell lines. Differentially expressed genes with potential shared ERα/PR regulatory binding sites, as defined by Khushi et al., are outlined in pink dashed lines. Differentially expressed genes are those with *p* < 0.05 and |log_2_(FC)|>1, where fold change is relative to matched tumors or cell lines expressing ERα WT. **c** Quantification of IRS1 mRNA expression in MCF7 and T47D cell variants, relative to T47D ERα WT expression levels. Data represent the average of 3 biological replicates with error bars indicating SEM. *P*-values comparing cell variants are indicated. Asterisks within bars indicate statistically significant differences between HD and PR-stimulated treatments within a given cell variant (*p < 0.05).

To determine the clinical relevance of the transcriptomal changes observed in MCF7 and T47D cell lines, we analyzed de-identified hormone receptor-positive breast cancer patient tumor RNA-seq data obtained from the publicly available MET500 and Personal Oncogenomics 570 (POG570) datasets^24,25^. Ten datasets from tumors containing ERα Y537S mutations were analyzed for differential gene expression relative to site-matched ERα WT tumor datasets, which identified 2043 differentially expressed genes in the context of ERα Y537S (Supplementary Fig. 6, Supplementary Table 4). Of these, 18 genes were also differentially expressed in MCF7 (2.4-fold over-enrichment, *p* = 0.2831 based on hypergeometric distribution analysis) and 14 in T47D cells expressing ERα Y537S (4.2-fold over-enrichment, *p* = 0.0104, Fig. 3a, b). Notably, most of the differentially expressed genes were upregulated (as opposed to downregulated) in both patient tumors and cell line data, and this upregulation occurred independent of ERα or PR stimulation (Fig. 3a, b, Supplementary Table 4). This highlights the known ligand-independent activity of ERα Y537S.

Of the genes differentially expressed in both cell lines and patient tumors containing ERα Y537S mutations, only four contained potential ERα-PR shared regulatory binding sites, as identified by Khushi et al. These were *DEGS2* (Delta-4-Desaturase, Sphingolipid 2), *FMN1* (Formin 1), *IRS1* (Insulin Receptor Substrate 1), and *KCNK15* (Potassium Two Pore Domain Channel Subfamily K Member 15), all of which were expressed ~2- to 4-fold more in MCF7 ERα Y537S-hom cells (independent of hormone stimulation) and patient tumors than their respective ERα WT counterparts (Fig. 3a, outlined in dashed lines). Previous studies implicate IRS1 in crosstalk interactions with both ERα and PR, as well as pro-proliferative signaling in breast cancer^26–30^. Additionally, Li et al. characterized a similar increase in IRS1 expression in ERα Y537S cells, as well as an upregulated insulin-like growth factor (IGF) signature corresponding with increased downstream PI3K-AKT signaling. Interestingly, these IRS1-related gene expression changes were also observed in cells expressing another common ERα mutation, namely ERα D538G^31^.

Given the prior interest in IRS1, we further confirmed *IRS1* expression at the mRNA level through RT-qPCR (Fig. 3c). Although RNA-seq sensitivity identified differential expression of *IRS1* in only MCF7 ERα Y537S-hom, specific analysis with RT-qPCR identified that *IRS1* is upregulated in both T47D and MCF7 cells expressing ERα Y537S-het or -hom. Though baseline levels of IRS1 expression are lower in T47D cells (which likely explains its absence from RNA-seq analysis), there is a ~40-fold increase in IRS1 expression in T47D ERα Y537S-hom cells, relative to ERα WT. Similar to the ERα/PR proximity-based interaction discussed in the previous section, IRS1 expression decreased slightly in the T47D ERα Y537S-hom cells upon PR stimulation, again highlighting a particular cell line-dependent PR sensitivity (Fig. 3c).

### Occupation of ERα and PR at *IRS1* regulatory binding sites is altered in the context of the ERα Y537S mutation

**Fig. 4:**
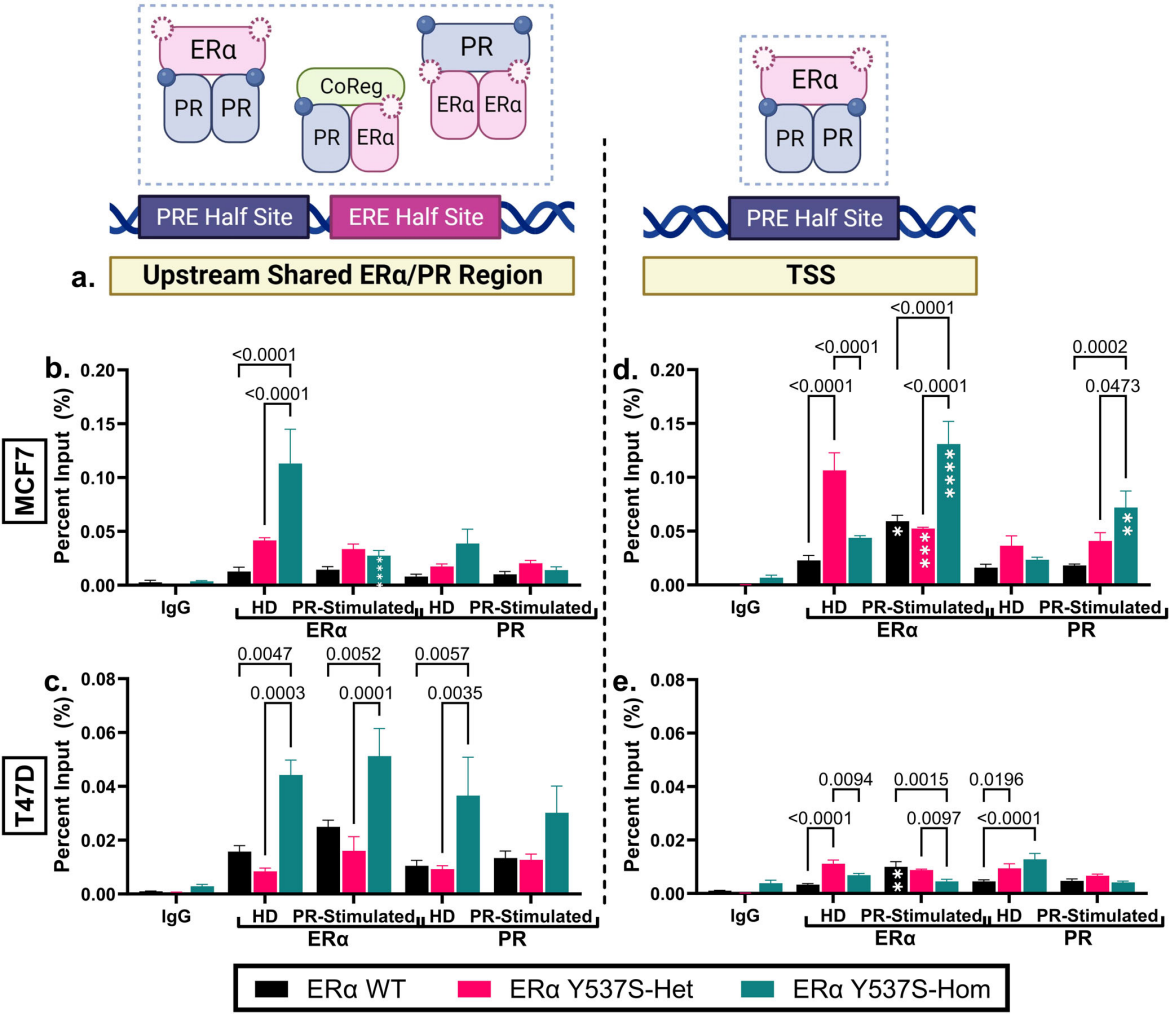
ERα and PR chromatin binding at *IRS1* is altered in the context of ERα Y537S. **a** Diagram depicting *IRS1* chromatin sites assessed for ERα and PR binding using ChIP-qPCR. Potential binding conformations at each site are outlined in dashed lines. Graphic created with Biorender.com. Chromatin binding of ERα and PR at distinct regions of *IRS1* is depicted in **a** for **b**, **d** MCF7, and **c**, **e** T47D cell variants. For all chromatin immunoprecipitation analyses (**b**–**e**), data represents the % of input chromatin analyzed. Data represent the average of 3 biological replicates with error bars indicating SEM. *P*-values comparing cell variants are indicated. Asterisks within bars indicate statistically significant differences between HD and PR-stimulated treatments within a given cell variant (*p < 0.05, **p < 0.005, **p < 0.001, **p < 0.0001).

To determine if differential expression of *IRS1* in the context of the ERα Y537S mutation could be a result of altered ERα/PR crosstalk, we next assessed ERα and PR genomic binding at two chromatin binding sites depicted in Fig. 4a and referred to here as *IRS1*-Upstream (distal location, contains both an estrogen response element (ERE) half site and a progesterone response element (PRE) half site) and *IRS1*-TSS (proximal location near transcription start site (TSS), contains a PRE half site). In HD MCF7 and HD or PR-stimulated T47D cells, ERα and PR chromatin occupancy at *IRS1*-Upstream increased significantly in the context of ERα Y537S-hom compared to either ERα WT or Y537S-het (Fig. 4b, c). This suggests that the ERα Y537S mutation not only alters the transcription factor activity of ERα but also that of PR. Importantly, these ERα Y537S-associated increases in PR chromatin occupancy at *IRS1*-Upstream occur despite the absence of PR ligand, highlighting a role of ERα Y537S in driving hormone-independent PR activity.

While binding of ERα and PR at *IRS1*-Upstream decreased upon PR-stimulation in MCF7 ERα Y537S-hom (Fig. 4b), chromatin binding of both proteins increased proportionally at *IRS1*-TSS under the same conditions (Fig. 4d). These findings highlight an R5020-dependent preference for ERα/PR binding at IRS1-TSS, specifically in MCF7 cells expressing ERα Y537S-hom. This may be because this site contains only a PRE (Fig. 4a), which may require liganded PR for binding to occur.

### Altered ERα/PR chromatin binding at *IRS1* corresponds with altered IRS1 expression

**Fig. 5:**
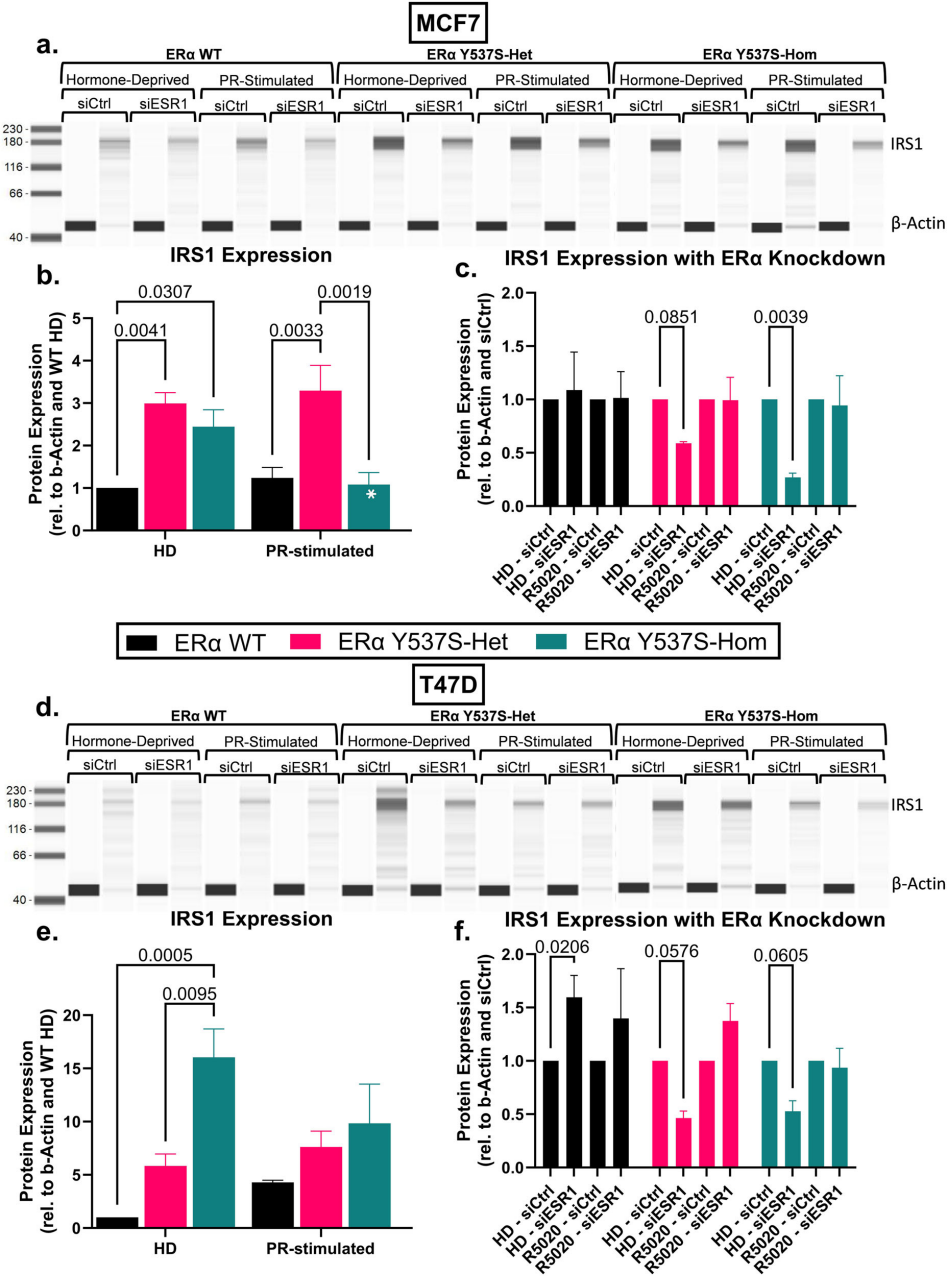
IRS1 protein expression is increased in the context of the ERα Y537S mutation. Quantification of IRS1 protein in **a**–**c** MCF7 and **d**–**f** T47D cell variants. Representative lane images from ProteinSimple WES quantification for **a** MCF7 and **d** T47D cell variants. Paired lanes indicate β-actin loading control and IRS1 expression for cell variants transfected with a negative control siRNA or si*ESR1*. **b**, **e** Quantification of IRS1 expression based on signal/noise ratio from WES quantification, with normalization to β-actin loading control and hormone-deprived ERα WT. **c**, **f** Comparison of relative IRS1 expression in siCtrl and si*ESR1* samples, with normalization to β-actin loading control and to paired siCtrl, to assess the effect of ERα knockdown on IRS1 expression in each cell variant and treatment. Data represent the average of 3 biological replicates with error bars indicating SEM. *P*-values comparing cell variants are indicated. Asterisks within bars indicate statistically significant differences between HD and PR-stimulated treatments within a given cell variant (*p < 0.05).

While ERα Y537S-associated changes to ERα/PR crosstalk as related to chromatin occupancy of the two transcription factors are interesting on their own, we next assessed the expression of IRS1 to determine if these cistromal changes translated to altered protein expression. As noted previously, *IRS1* mRNA expression was manyfold higher in both T47D and MCF7 cells expressing ERα Y537S-het or -hom (Fig. 3c). Similarly, IRS1 protein expression was significantly increased in both MCF7 and T47D cells expressing ERα Y537S-het or -hom under hormone-deprived conditions (Fig. 5a, b, d, e). Interestingly, PR-stimulation partially reduced IRS1 expression in MCF7 cells expressing homozygous ERα Y537S, suggesting that PR activity may negatively regulate IRS1 expression (Fig. 5b). This may correspond with the increased ERα/PR preference for the PRE-containing IRS1-TSS binding site discussed previously (Fig. 4d).

To confirm that increased expression of IRS1 was associated with ERα Y537S activity, we assessed IRS1 protein expression after small interfering RNA (siRNA) knockdown of *ESR1* (knockdown confirmation in Supplementary Fig. 7). IRS1 expression decreased by ~50% in HD MCF7 and T47D ERα Y537S-het or -hom cells upon *ESR1* knockdown (Fig. 5c, f). PR stimulation restored IRS1 protein levels upon *ESR1* knockdown, highlighting a shared role of ERα Y537S and PR in regulating IRS1 expression (Fig. 5c, f). Interestingly, *ESR1* knockdown did not affect IRS1 expression in MCF7 ERα WT cells, and IRS1 expression actually increased in T47D ERα WT cells upon *ESR1* knockdown (Fig. 5c, f). Together, these results highlight an ERα Y537S-specific mechanism by which IRS1 expression is elevated in the context of the endocrine therapy resistance-associated mutation.

### Inhibition of IRS1 by NT-157 depletes the proliferative effect of the ERα Y537S mutation

**Fig. 6:**
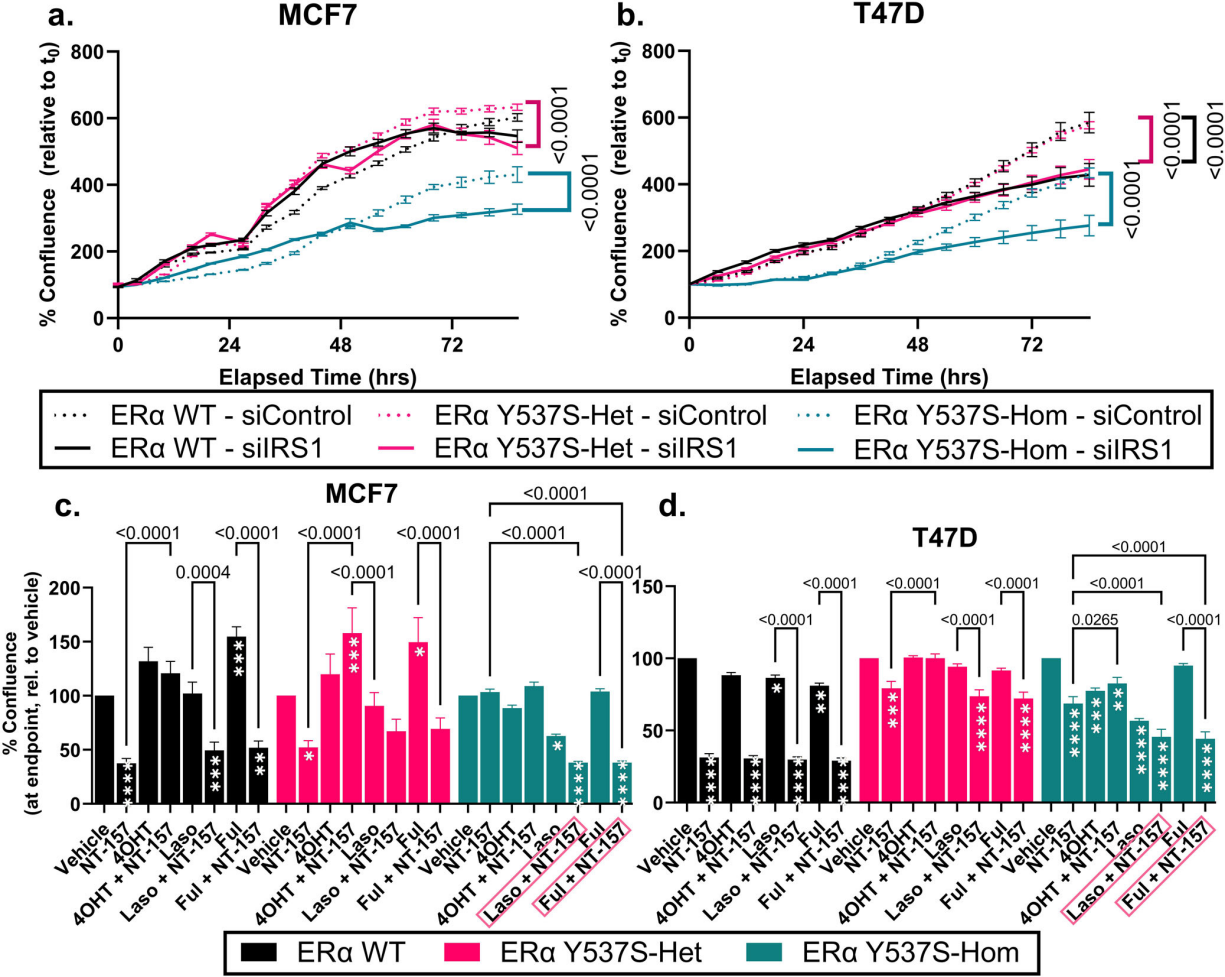
IRS1 depletion or inhibition effectively inhibits proliferation of cells expressing ERα Y537S. Proliferation, as measured by % cell confluence relative to the initial timepoint (t_0_), upon siRNA knockdown of *IRS1* is shown in **a** MCF7 and **b** T47D cell lines. Proliferation of **c** MCF7 and **d** T47D cells treated with Vehicle, 4OHT, laso, or ful, alone or in combination with NT-157. Graphs show % confluence after 5 days of treatment, normalized to vehicle. Data represent the average of 3 biological replicates with error bars indicating SEM. *P*-values indicate a significant change in proliferation at endpoint compared to each respective single drug treatment (NT-157, 4OHT, laso, or ful alone). Asterisks within bars indicate a significant change in proliferation compared to vehicle treatment (*p < 0.05, **p < 0.005, **p < 0.001, **p < 0.0001).

To assess the functional significance of upregulated expression of IRS1 in the context of the ERα Y537S mutation, we assessed the effect of *IRS1* siRNA knockdown on proliferation of MCF7 and T47D cells expressing ERα WT, ERα Y537S-het, or ERα Y537S-hom (knockdown confirmation in Supplementary Fig. 8). Depletion of *IRS1* resulted in significantly decreased proliferation of both MCF7 and T47D cells expressing ERα Y537S, highlighting a potential therapeutic sensitivity of this endocrine therapy-resistant mutation (Fig. 6a, b).

Due to the antiproliferative effect of IRS1 knockdown in MCF7 and T47D cells expressing ERα Y537S, we next investigated if NT-157, a small molecule inhibitor of IRS1, would similarly reduce cell growth. NT-157 functions by degrading IRS1 and IRS2, leading to the inhibition of IGF-1R/IRS1/2, PI3K, and AXL-mediated signaling pathways^30,32,33^. NT-157 reduces in vitro cell growth and in vivo tumor growth in models of uveal melanoma, chronic myeloid leukemia, myeloproliferative neoplasms, osteosarcoma, and prostate cancer^33–38^. Additionally, recent studies have found NT-157 to inhibit proliferation in breast cancer cell lines, including those resistant to tamoxifen^26,39^. Though NT-157 has yet to be approved for use clinically, several IGF-1R inhibitors, including cixutumumab, have proved to be well-tolerated and effective in stabilizing several advanced cancers including Ewing’s sarcoma and adrenocortical carcinoma^40–42^.

As a single treatment, 5 uM NT-157 effectively reduced the proliferation of all MCF7 and T47D ERα cell variants apart from MCF7 ERα Y537S-hom (Fig. 6c, d). 5 uM NT-157 falls within the range of effective doses used in previous studies in breast and prostate cancer cell lines^38,39^. To determine the efficacy of combining ET with IRS1 inhibition via NT-157, MCF7 and T47D ERα cell variant proliferation was assessed over 5 days of treatment with 100 nM 4OHT (a SERM), 100 nM Laso (a novel SERM), or 1 uM Ful (a SERD), each alone or in combination with 5 uM NT-157.

Across both MCF7 and T47D cell variants, proliferation was largely unaffected by treatment with 4OHT, and combined treatment with 4OHT and NT-157 did not improve inhibition beyond that of single NT-157 treatment (Fig. 6c, d). In fact, NT-157 alone effectively reduced the proliferation of MCF7 and T47D ERα WT cells by more than 50%; combined treatment of NT-157 with all SERMs/SERDs tested did little to enhance this inhibitory effect in the ERα WT context (Fig. 6c, d, black). MCF7 and T47D ERα Y537S-het cells were similarly responsive to NT-157 treatment as ERα WT cells and combination treatments did not add to the antiproliferative effect of NT-157 alone (Fig. 6c, d, pink). Interestingly, in both MCF7 and T47D ERα Y537S-hom cells, a combination of either lasofoxifene or fulvestrant with NT-157 resulted in additive inhibition beyond that of NT-157 alone (Fig. 6c, d, teal). Overall, the striking effect of inhibition of IRS1 via NT-157, alone or in combination with lasofoxifene or fulvestrant, may offer a treatment avenue for ET-resistant breast cancers.

## Discussion

Prior research on the constitutively active ERα Y537S mutation has understandably focused on ERα function, vastly advancing our knowledge of the mutation’s contribution to ET resistance^7,16,43–46^. However, the effect of ERα Y537S on the complex relationship known as ERα/PR crosstalk has previously not been thoroughly investigated. In this project, we aimed to determine the effects of the ERα Y537S mutation on ERα/PR crosstalk and resulting transcriptional activity, and to elucidate how this unique interaction contributes to ET resistance in ERα-positive breast cancer.

A comparison of transcriptomes between MCF7 and T47D cell variants supports previous studies highlighting the two cell lines’ vastly different expression profiles^47–49^. However, both MCF7 and T47D cells expressing homozygous ERα Y537S differentially expressed hundreds of genes when each was compared to ERα WT. Notably, far fewer genes are differentially expressed when comparing ERα Y537S-het cell variants to ERα WT cell variants (Fig. 2). This highlights the importance of including heterozygous and homozygous models when studying a mutation such as ERα Y537S, which is clinically observed as mosaic expression within a patient’s cancer.

**Fig. 7:**
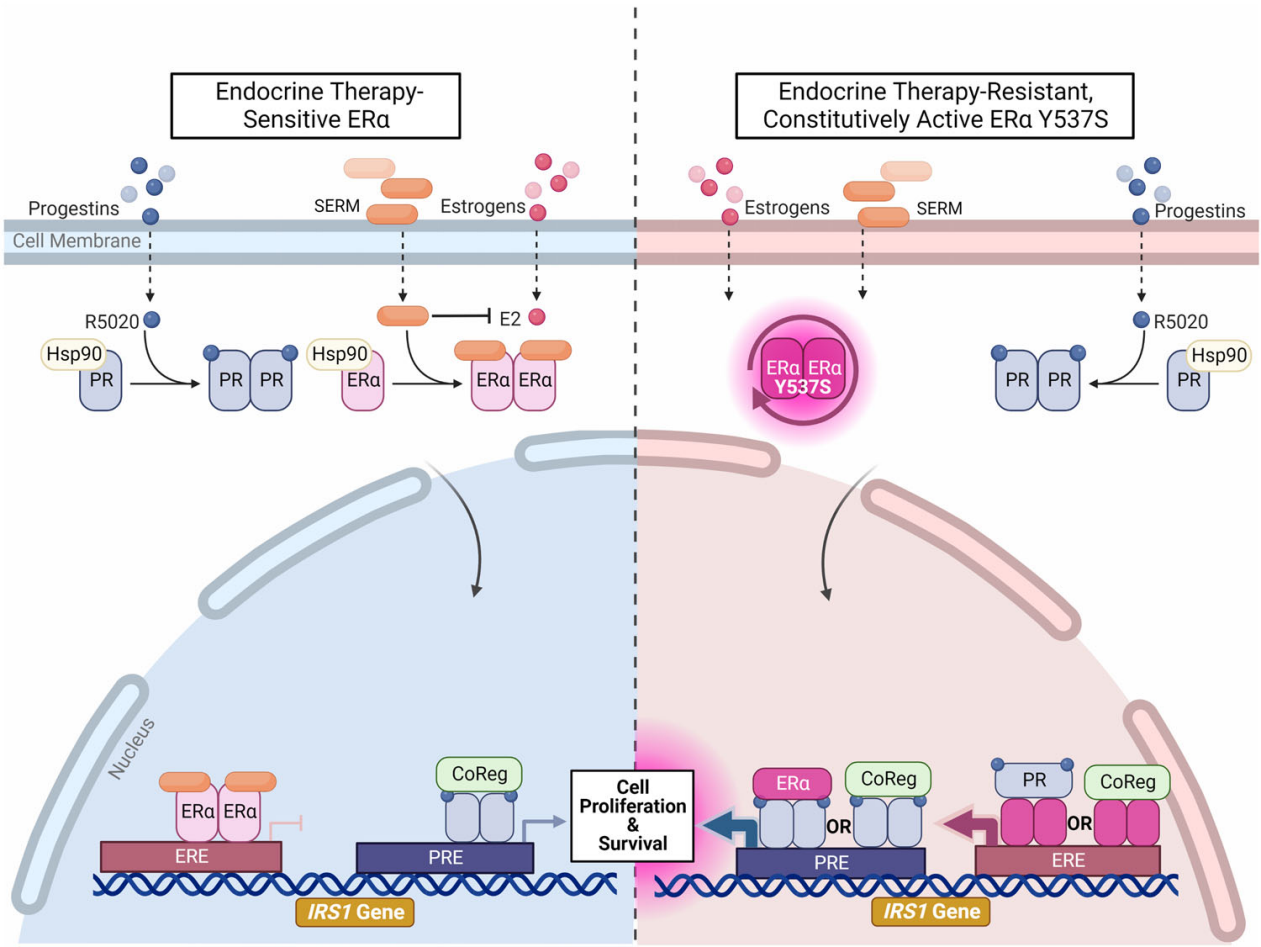
Proposed mechanism for IRS1-dependent cell proliferation in the context of the ERα Y537S mutation. **Left panel:** In ET sensitive (ERα WT) cells, selective estrogen receptor modulators (SERMs) competitively bind to ERα, blocking E2. SERM-bound ERα is still able to dimerize and bind to chromatin sites, but the antagonistic functions of SERMs prevent recruitment of co-activators required to drive transcription of target genes, including *IRS1*. Some transcription of *IRS1* occurs through PR-dependent transcription. **Right panel:** In ET-resistant (ERα Y537S) cells, ERα is constitutively active and has reduced affinity for SERM binding. *IRS1* transcription is high due to activity at both EREs and PREs, both by independent ERα and PR transcription factor activity as well as by the two receptors physically interacting as coregulators (CoReg). This overdrive of *IRS1* expression contributes to a reliance on expression of this signaling pathway component for continued cell proliferation and survival in ET-resistant cells. However, PR stimulation in the context ERα Y537S of may result in a partial reduction in IRS1 expression. Graphic created with Biorender.com.

Given the imperfect cell line model systems described above, we then compared these findings to publicly available patient data and identified 4 gene expression changes aligned with potential ERα-PR shared regulatory binding sites (Fig. 3a)^24,25,50^. Of these, IRS1 proved most notable; ERα and PR chromatin occupancy at *IRS1* shared ERα/PR binding sites increased significantly in the context of ERα Y537S-hom, highlighting that the ERα Y537S mutation not only alters the transcription factor activity of ERα but also that of PR (Fig. 4). Interestingly, both ERα Y537S and PR chromatin occupancy is present at a site with only a PRE half site and no ERE, indicating the presence of ERα-PR regulatory complexes in which ERα Y537S may act as a co-regulator for PR (Fig. 4d)^9,51,52^. Here, we propose a mechanism by which ERα Y537S results in constitutive activity of ERα, even in the presence of SERMs, leading to increased ERα-PR regulatory complexes driving increased IRS1 expression, through which cell proliferation and survival is enforced (Fig. 7).

To further confirm the role of IRS1 in maintaining cell proliferation in the context of ERα Y537S, we assessed the small molecule IRS1 inhibitor NT-157 in MCF7 and T47D ERα cell variant drug screens. NT-157 effectively reduced cell proliferation in MCF7 and T47D cells expressing ERα WT or ERα Y537S (Fig. 6). As mentioned previously, NT-157 is a degrader of both IRS1 and IRS2, which likely contributes to the reduced proliferation observed in MCF7 and T47D ERα WT cells, despite relatively low levels of IRS1 expression in the ERα WT context compared to ERα Y537S-expressing cells (Figs. 5 and 6c, d). It’s possible that NT-157-induced degradation of IRS2 is sufficient to reduce cell proliferation in ERα WT cells, though further investigation is required to fully understand the mechanism of action of NT-157 in this context.

Co-targeting ERα via SERM or SERD treatment and IRS1 via NT-157 had an additive antiproliferative effect on cells expressing homozygous ERα Y537S, indicating a potential treatment avenue for restoring ET sensitivity to resistant breast cancers expressing ERα Y537S. Combination SERM/SERD and NT-157 treatments did not have a similar additive effect on proliferation of ERα WT or ERα Y537S-het cells. The explanation for the difference in compound sensitivity between heterozygous and homozygous ERα Y537S cells is three-fold:

1. The ERα Y537S-het and -hom cell lines were derived separately (see Materials & Methods).
2. Heterozygous and homozygous ERα Y537S phenotypes are characteristically unique (as described throughout this manuscript).
3. Single NT-157 treatment has a consequentially anti-proliferative effect on ERα Y537S-het cells, which seemingly cannot be improved upon.

Overall, these findings highlight a treatment sensitivity that is particularly strong in the context of the ERα Y537S mutation, which supports our proposed mechanism by which IRS1 upregulation drives cell proliferation in the context of the ERα Y537S mutation in response to increased ERα/PR crosstalk. Importantly, the antiproliferative effect of IRS1 inhibition by NT-157 is further enhanced by combined treatment with the novel SERM lasofoxifene or the SERD fulvestrant, highlighting that ET sensitivity is restored by co-targeting this pathway in resistant ERα Y537S cells (Fig. 6a, b, teal). These findings indicate a potential therapeutic avenue through which treatment sensitivity may be restored in ET-resistant breast cancers.

## Methods

### Cell lines and growth conditions

MCF7 and T47D cells (originally obtained from the American Type Culture Collection, ATCC) were previously edited using adeno-associated virus recombinant viral vectors to express the heterozygous *ESR1* mutation known as ERα Y537S (ERα Y537S-het). Homozygous ERα Y537S mutant cell lines were generated using CRISPR-Cas9 editing (ERα Y537S-hom). MCF7 parent cells (MCF7 ERα WT) and MCF7 ERα Y537S-het were generated and gifted by Ben Ho Park, originally at Johns Hopkins University and now at Vanderbilt University^16^. MCF7 ERα Y537S-hom cells were generated and gifted by Sarat Chandarlapaty at Memorial Sloan Kettering Cancer Center^53^. T47D parent cells (T47D ERα WT) and T47D ERα Y537S-het cells were generated and gifted by Steffi Oesterreich at the University of Pittsburgh^16^. T47D ERα Y537S-hom were generated by David Shapiro at the University of Illinois at Urbana-Champaign originally and were gifted from Carol Lange at the University of Minnesota^54^.

MCF7 cell variants were maintained in phenol red-free Dulbecco’s Modified Eagle Medium (DMEM) containing 5% fetal bovine serum (FBS), 1% Pen/Strep, and 1% L-Glutamine. T47D ERα WT and ERα Y537S-het cell lines were maintained in phenol red-free Roswell Park Memorial Institute 1640 (RPMI) media containing 10% FBS and 1% Pen/Strep. T47D Y537S-hom cells were maintained in phenol red-free Modified Eagle Medium (MEM) containing 10% charcoal-stripped serum (CSS), 1% Pen/Strep, and 0.2 ug/uL puromycin for continuous selection. MCF7 cell variants were cultured in DMEM containing 10% CSS, and T47D cell variants were cultured in RPMI containing 10% CSS for 48 hours prior to experimentation.

All cell lines were validated for ERα receptor status (WT, Y537S-heterozygous, or Y537S-homozygous) through next generation sequencing (NGS) completed by the University of Illinois at Chicago Genome Research Core. Cells were tested for mycoplasma after thawing fresh cells and prior to beginning experimentation. Testing was completed using the MycoAlert Mycoplasma Detection Kit (Lonza Bioscience #LT07-318).

### Compounds and antibodies

Promegestone (R5020, Perkin Elmer #NLP004005MG) was used for all assays in MCF7 and T47D cells. NT-157 (Selleck Chemical #S8228), 4-hydroxytamoxifen (4OHT, Sigma #94873), lasofoxifene (Laso, Sermonix Pharmaceuticals), and fulvestrant (Ful, Selleck Chemical #S1191) were used for confluence-based drug screen assays. Vehicle (ethanol) was used as a control for all experiments.

1:10 D8Q2J rabbit monoclonal antibody (Cell Signaling #8757) was used for the detection of PR isoforms PR-A and PR-B in proximity ligation assays (PLA). 1:10 F10 mouse monoclonal antibody (Santa Cruz Biotechnology #sc-8002) was used for the detection of ERα in PLA. F10 and anti-IRS1 mouse monoclonal antibody (Santa Cruz Biotechnology #sc-8038) were used for immunoblot detection of ERα and IRS1, respectively, both at 1:10 using the Bio-Techne ProteinSimple WES platform. 1:100 AC-15 mouse monoclonal antibody (Santa Cruz Biotechnology #sc-69879) was used for the detection of β-actin as a loading control in immunoblot detection. 4 ug/uL KD68 rat monoclonal antibody (variable stock concentration, originally generated by Greene et al.^55^ and produced and purified by the University of Chicago Flow Cytometry Core) was used for immunoblot detection of PR. 5 ug KD68 was used for chromatin immunoprecipitation (ChIP) to immunoprecipitate chromatin to which PR-A or PR-B was bound. The 5 ug ERα C-terminal antibody from Epicypher (#13-2012) was used for ERα immunoprecipitation in ChIP. 5 ug normal rabbit IgG and normal rat IgG (Santa Cruz Biotechnology #sc-2027 and #sc-2026, respectively) were used as negative control antibodies for Epicypher ERα C-terminal and KD68, respectively.

### Proximity ligation assay (PLA)

After culturing MCF7 and T47D cells in hormone-starved conditions (charcoal-stripped media) for 48 h, 5000 cells/well were plated into each well of an 8-well glass bottom chamber slide. Cells were then treated with vehicle or 10 nM R5020 for PR stimulation for 24 h. Cells were fixed using 37% formaldehyde, followed by permeabilization with 100% methanol. Proximity ligation was performed according to the Millipore Sigma Duolink^®^ PLA Fluorescence Protocol using the Duolink^®^ Anti-rabbit PLUS probe (#DUO92002, to detect PR through a 1:10 dilution of D8Q2J antibody), Duolink^®^ Anti-mouse MINUS probe (#DUO92004, to detect ERα through a 1:10 dilution of F10 antibody), Duolink^®^ Red Fluorescence Detection Reagents (#DUO92008), Duolink^®^ Wash Buffers (#DUO82049), and Invitrogen SlowFade™ Gold antifade mounting reagent (#S36940). Image acquisition was completed by the University of Chicago Integrated Light Microscopy Core with a Leica SP8 3D STED laser scanning confocal microscope (Leica Microsystems, Inc., Buffalo Grove, IL).

### RNA extraction and sequencing (RNA-seq)

MCF7 and T47D cell variants were plated at 2e5 cells/well of a 6-well plate in hormone-deprived conditions (charcoal-stripped media). After 48 h, cells were treated with vehicle or 10 nM R5020 for PR stimulation and collected via trypsinization after 2 h of treatment. RNA was extracted using the Qiagen RNeasy Plus kit (#74104) according to the manufacturer’s protocol. RNA concentrations were quantified by Nanodrop nucleic acid measurement.

Quantitative reverse transcription polymerase chain reaction (RT-qPCR) was used to quantify RNA expression at known ERα target genes and to ensure high-quality RNA for library preparation and sequencing. cDNA was synthesized from 1 ug RNA using 5X Quanta Bio qScript Mastermix (#95048) according to the Quanta Bio qScript protocol. Applied Biosystems™ TaqMan™ Fast Advanced Master Mix (#4444557) and Human Beta-2-Microglobulin endogenous control (B2M, #4326319E) were used for RT-qPCR using a Roche Step-One Real-Time PCR machine. IDT primers were used for the detection of *SGK1* and *IRS1* (Hs.PT.58.19153459.gs and Hs.PT.58.39283803, respectively). Reactions were run in triplicate, with 3 biological replicates per sample.

RNA library preparation for sequencing was completed using the KAPA mRNA HyperPrep Kit (#KR1352) according to the manufacturer’s protocol. Sequencing was completed on the Illumina NovaSeq 6000 by the University of Chicago Functional Genomics core (RRID: SCR_019196).

### RNA-seq analysis

RNA-seq data were uploaded to the Galaxy platform and analyzed using the public server at usegalaxy.org^56^. Sequencing files were mapped to the hg19 human reference genome using Bowtie2 and read counts per gene were generated from the aligned sequences using HTSeq-Count. DESeq2 was used to determine differentially expressed genes between each cell variant and between each treatment. Raw files, HTSeq-Counts, and DESeq2 differentially expressed genes are publicly available through NCBI Gene Expression Omnibus (GEO accession #: GSE243454).

Analyzed MCF7 and T47D RNA-seq data were compared to de-identified patient tumor RNA-seq data obtained from the publicly available MET500 and Personal Oncogenomics 570 (POG570) datasets^24,25^. Specific dataset IDs can be found in Supplementary Table 1. DESeq2 was used to compare differential gene expression between patient tumors harboring ERα Y537S mutations (4 from MET500 and 6 from POG570) and those with ERα WT (31 from MET500 and 32 from POG570).

### Chromatin immunoprecipitation (ChIP) and analysis by qPCR (ChIP-qPCR)

After culturing MCF7 and T47D cells in hormone-deprived conditions (charcoal-stripped media) for 48 h and treating with vehicle or 10 nM R5020 for 1 h, ~10e6 cells were harvested in ice-cold PBS. Cells were crosslinked in 1% formaldehyde in PBS. Crosslinking was quenched by the addition of glycine at a final concentration of 125 mM. Crosslinked cell pellets were snap frozen and stored at −80 °C.

For each ChIP experimental replicate, ~20e6 crosslinked cells (from 2 crosslinked aliquots) were lysed in lysis buffer with PICS III using sonication (high, 30 seconds on/off, for 5 intervals of 10 min). 5% of lysate was reserved for input control and snap frozen to store at −80 °C. Lysates were diluted to 1 ug/uL protein based on Nanodrop A280 concentrations and divided into 1 mL aliquots. 5 ug of the appropriate antibodies (KD68 for PR ChIP, Epicypher ERα C-terminal for ERα ChIP, rat IgG for PR negative control, and rabbit IgG for ERα negative control) were added to the appropriate lysate aliquots and rotated at 4 °C overnight. Protein-chromatin was isolated and eluted using protein G beads. Eluted ChIP samples were incubated with RNAse A and Proteinase K to reverse the crosslinked protein-chromatin. Input samples and ChIP DNA was purified using a Qiagen QIAquick PCR Purification Kit, and purified DNA samples were eluted in 30 uL nuclease-free water.

Input and ChIP purified DNA was quantified using IDT primers specific for probable regions of shared chromatin binding by ERα and PR, as identified by Khushi et al. and consistent with candidate genes identified from RNA-seq and siRNA knockdown experiments^50^. Primer sequences are available in Supplementary Table 2. Quantabio PerfeCta^®^ SYBR^®^ Green FastMix Reaction Mix with ROX™ was used for qPCR reactions using a Roche Step-One Real-Time PCR machine. Reactions were run in triplicate, with 3 biological replicates per sample. qPCR Ct results were averaged and normalized to the endogenous control R18S (ΔCt_mean_). Input ΔCt_mean_ values were adjusted to consider the percent of the sample taken for input (5%), calculated as ΔCt_mean(input)_ - log_2_(20). ΔΔCt_mean_ for each ChIP condition was calculated as the difference between the corresponding adjusted ΔCt_mean(input)_ and the ΔCt_mean(ChIP)_. Percent input was then calculated as 100(2^ΔΔCt^).

### Immunoblotting

Cells were lysed using M-PER™ Mammalian Protein Extraction Reagent (Thermo Scientific #78501) containing cOmplete™ EDTA-free Protease Inhibitor Cocktail (Roche #04693159001). Protein concentrations were quantified using the A280 Nanodrop program. For all immunodetection other than that of PR, lysates were prepared to a final concentration of 2 ug/uL and run for immunoblot detection using the Bio-Techne ProteinSimple detection reagents and 12–230 kDa Separation Module (Bio-Techne #SM-W001 and #DM-002). For PR immunoblotting, lysates were prepared with SDS-containing sample buffer such that 100ug of protein would be loaded per well of a 4–20% polyacrylamide gel (Bio-Rad #4568096) for electrophoresis, followed by membrane transfer. Images of blots shown in figures are representative of biological triplicate experiments and are derived from one blot (no splicing or duplication of lanes).

### siRNA knockdown

Dharmacon™ ON-TARGETplus non-targeting pool (#D-001810-10-05), *IRS1* SMARTpool (#L-003015-00-0005), and *ESR1* SMARTpool (#L-003401-00-0005) were used for siRNA knockdown. MCF7 and T47D cell variants were treated and transfected using Lipofectamine™ RNAiMAX (#13778150) after 48 h of hormone starvation in stripped media. Proliferation was quantified using the Incucyte S3 platform. siRNA screens were carried out at the University of Chicago Cell Screening Center (CSC, RRID: SCR_017914).

### Drug screening

NT-157, an IRS1 inhibitor, was prepared at a stock concentration of 100 mM in ethanol. MCF7 and T47D cell variants were hormone starved in charcoal-stripped media for 48 ho followed by treatment with 5 uM NT-157, alone or in combination with a) 100 nM 4OHT, b) 100 nM lasofoxifene (laso), or c) 1 uM fulvestrant (ful). Proliferation was measured over 5 days using the Incucyte S3 platform. Compound screens were carried out at the University of Chicago Cell Screening Center (CSC, RRID: SCR_017914).

### Statistical analysis

Biological triplicates were completed for each experiment (*n* = 3). Unless otherwise noted, data were analyzed by ordinary two-way ANOVA (α = 0.05) with Tukey’s multiple comparisons tests to compare between treatments within each cell line, as well as between cell lines for each treatment. For all analyses: *p < 0.05, **p < 0.005, ***p < 0.0005, or ****p < 0.0001. For graphs, datapoints indicate the mean value of 3 experimental replicates and error bars represent standard error (SE).

### Supplementary information

Supplementary Material

Related Manuscript File

## Data Availability

The datasets used during the current study are largely available in the supplementary files, and RNA-Seq data is publicly available through NCBI Gene Expression Omnibus (GEO accession #: GSE243454). Any data not included are available from the corresponding author upon reasonable request. Data used to identify potential overlapping chromatin binding sites of ERα and PR are available in Khushi, M., C.L. Clarke, and J.D. Graham, *Bioinformatic analysis of cis-regulatory interactions between progesterone and estrogen receptors in breast cancer*. PeerJ, 2014. 10.7717/peerj.654.

## Code availability

The workflow created and used for RNA-Seq analysis on the publicly accessible Galaxy platform is available at https://usegalaxy.org/u/rhuggins27/w/copy-of-rna-seq-data-processing.
