# Supplementary material for: ERα/PR crosstalk is altered in the context of the ERα Y537S mutation and contributes to endocrine therapy-resistant tumor proliferation
Source: NPJ Breast Cancer. 2023 Nov 30;9:96. doi: 10.1038/s41523-023-00601-7 (PMC10689488; doi:10.1038/s41523-023-00601-7)
Supplement: Supplementary file 1 — Supplementary Material [file 41523_2023_601_MOESM1_ESM.pdf]

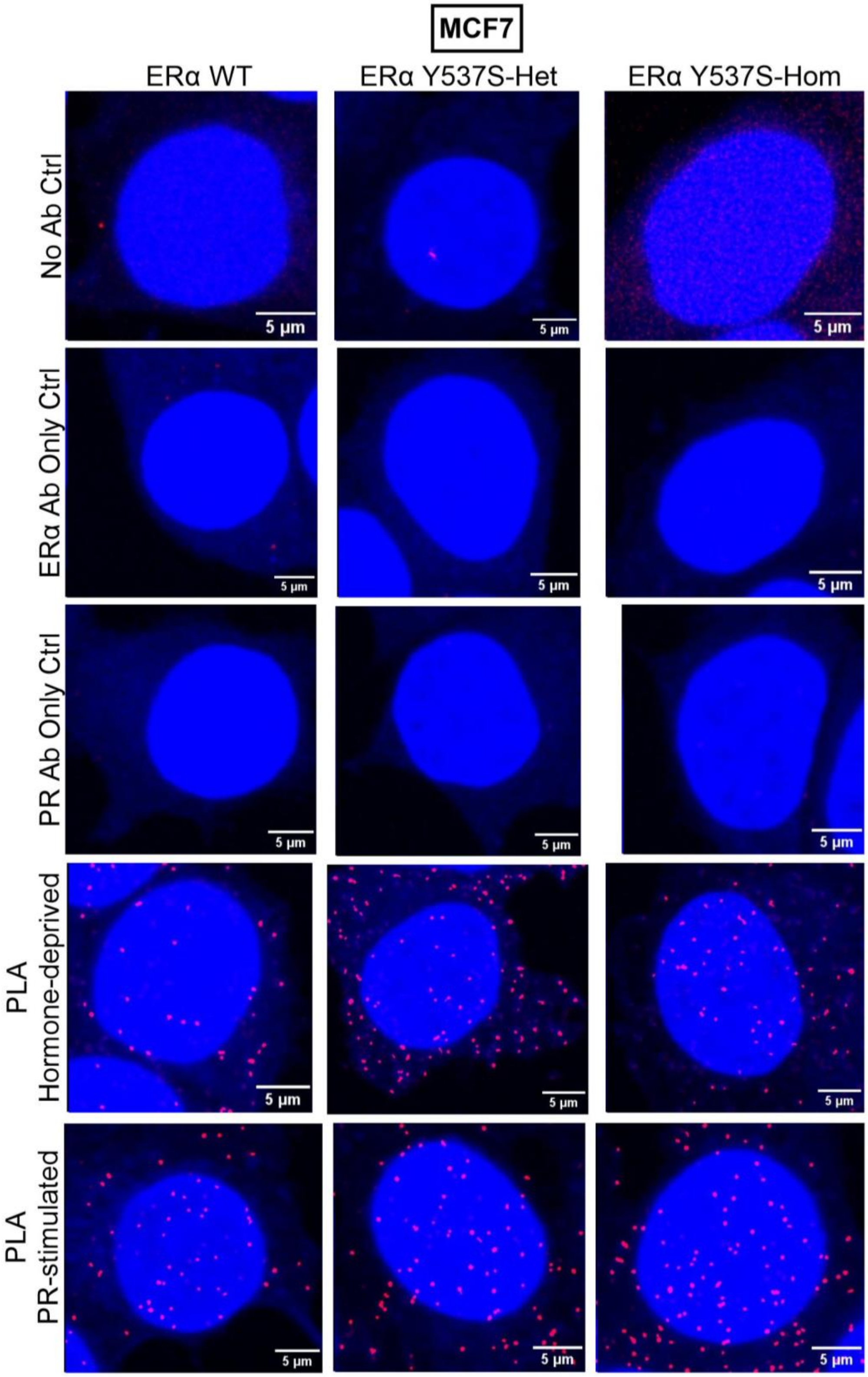

Supplementary Figure 1 Representative confocal images of PLA (red puncta) and DAPI (blue nuclei)-stained cells after hormone-deprived (vehicle) or PR-stimulated (10nM R5020) treatment in MCF7 cells. Negative controls include no antibody control (first row) and single antibody controls for ERα and PR (rows two and three, respectively).

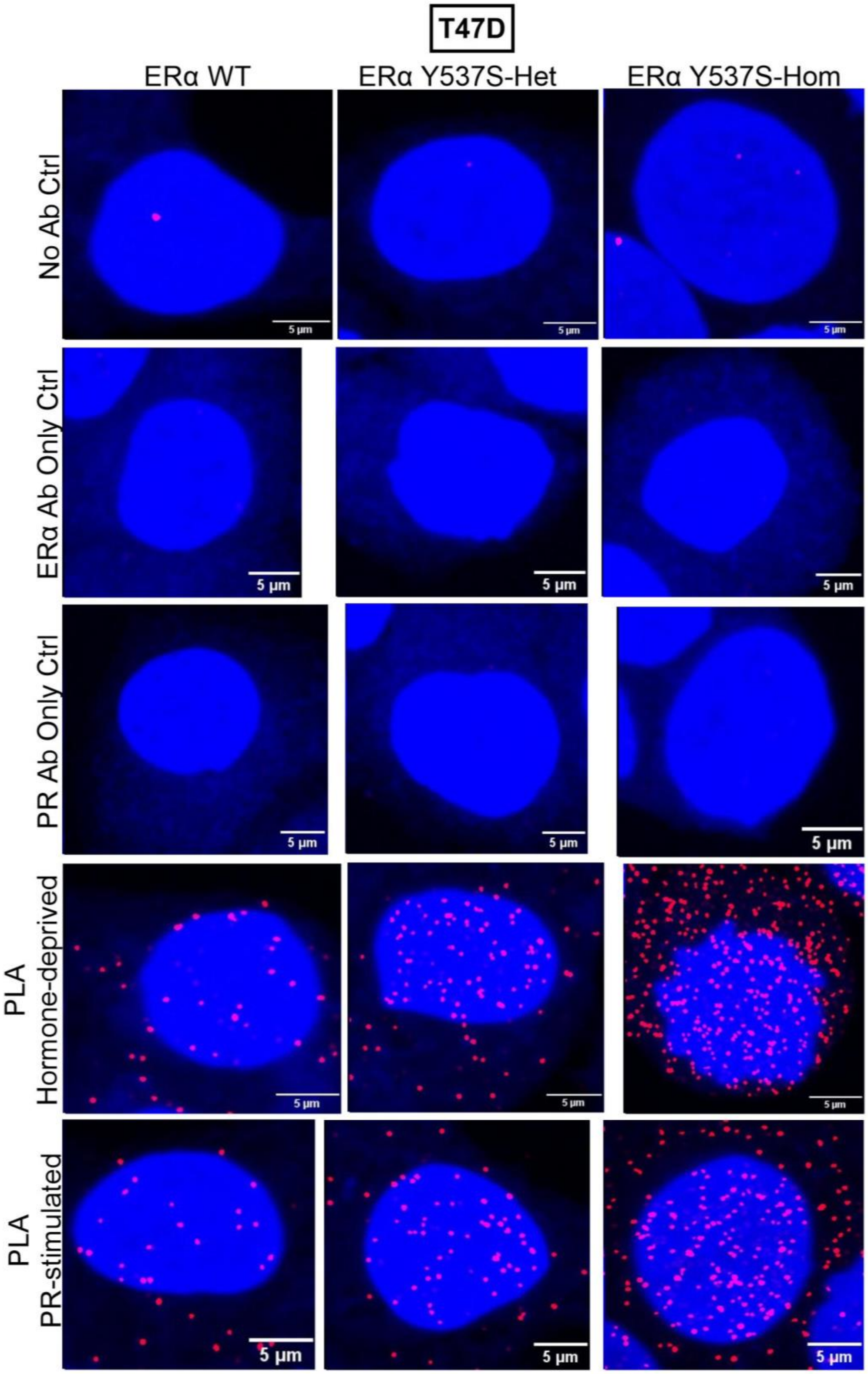

Supplementary Figure 2 Representative confocal images of PLA (red puncta) and DAPI (blue nuclei)-stained cells after hormone-deprived (vehicle) or PR-stimulated (10nM R5020) treatment in T47D cells. Negative controls include no antibody control (first row) and single antibody controls for ERα and PR (rows two and three, respectively).

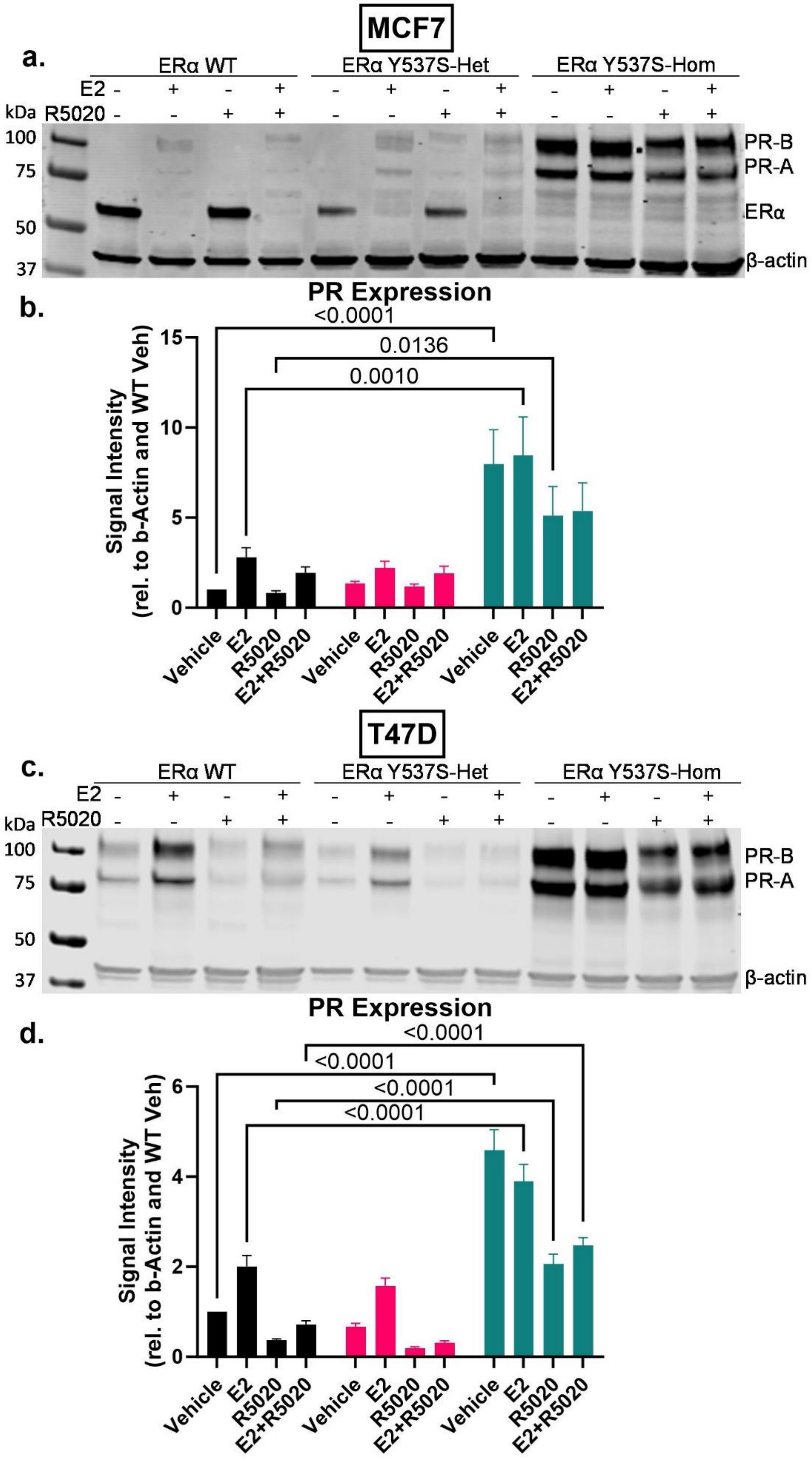

Supplementary Figure 3 Protein expression of PR in **a,b)** MCF7 and **c,d)** T47D ERα cell variants. PR protein expression is normalized to β-actin loading control and relative to ERα WT Vehicle. Data represents 3

replicates with error bars indicating standard error of the mean (SEM). P-values indicate significant difference in protein expression relative to ERα WT.

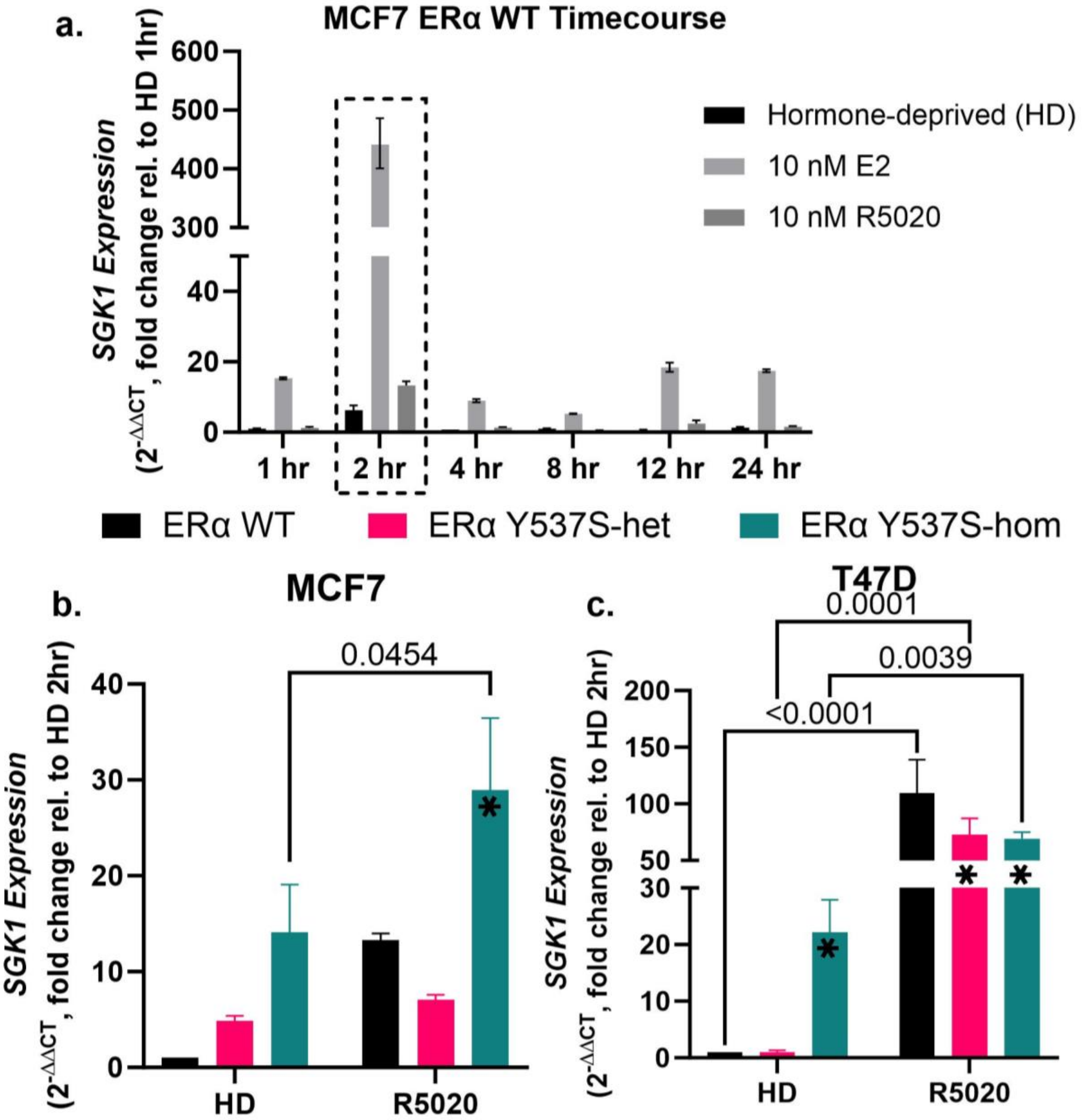

Supplementary Figure 4 Experimental rationale and sample validation for RNA-seq. **a)** Timecourse of MCF7 ERα WT cells treated with vehicle (hormone-deprived), 10 nM E2 (ERα agonism), or 10 nM R5020 (PR agonism) for 1, 2, 4, 8, 12, or 24 hours. Figure shows RT-qPCR quantification of SGK1 expression (a known ERα and PR target gene) at each timepoint. SGK1 expression at 2 hours is emphasized in dashed lines to highlight the selection of this timepoint for further experiments. Quantification of SGK1 expression as quality control of samples prepared for RNA-seq is shown for **b)** MCF7 and **c)** T47D cells expressing ERα WT, ERα Y537S-het, or ERα Y537S-hom. Statistical significance determined by two-way ANOVA with Tukey's multiple comparisons test. Data represents 3 replicates with error bars indicating standard error of the mean (SEM). P-

values shown comparing treatment effect. Asterisks within bars represent statistically significant difference in expression in ERα Y537S-het or -hom, when compared to ERα WT (* $p < 0.05$).

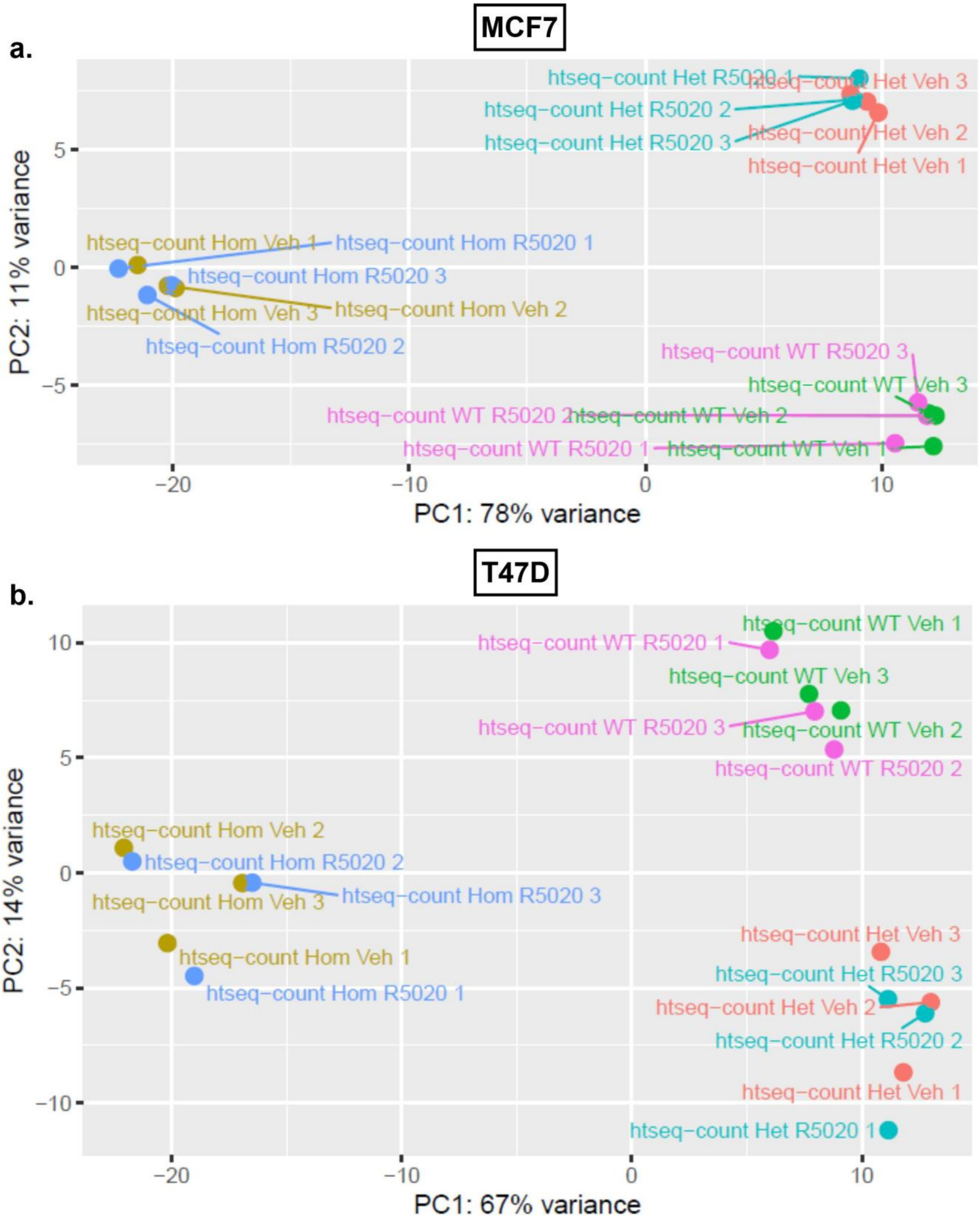

Supplementary Figure 5 PCA plots of RNA-seq replicates of **a)** MCF7 and **b)** T47D ERα WT, ERα Y537S-het, and ERα Y537S-hom cells treated with vehicle (hormone-deprived) or R5020 (PR-stimulated).

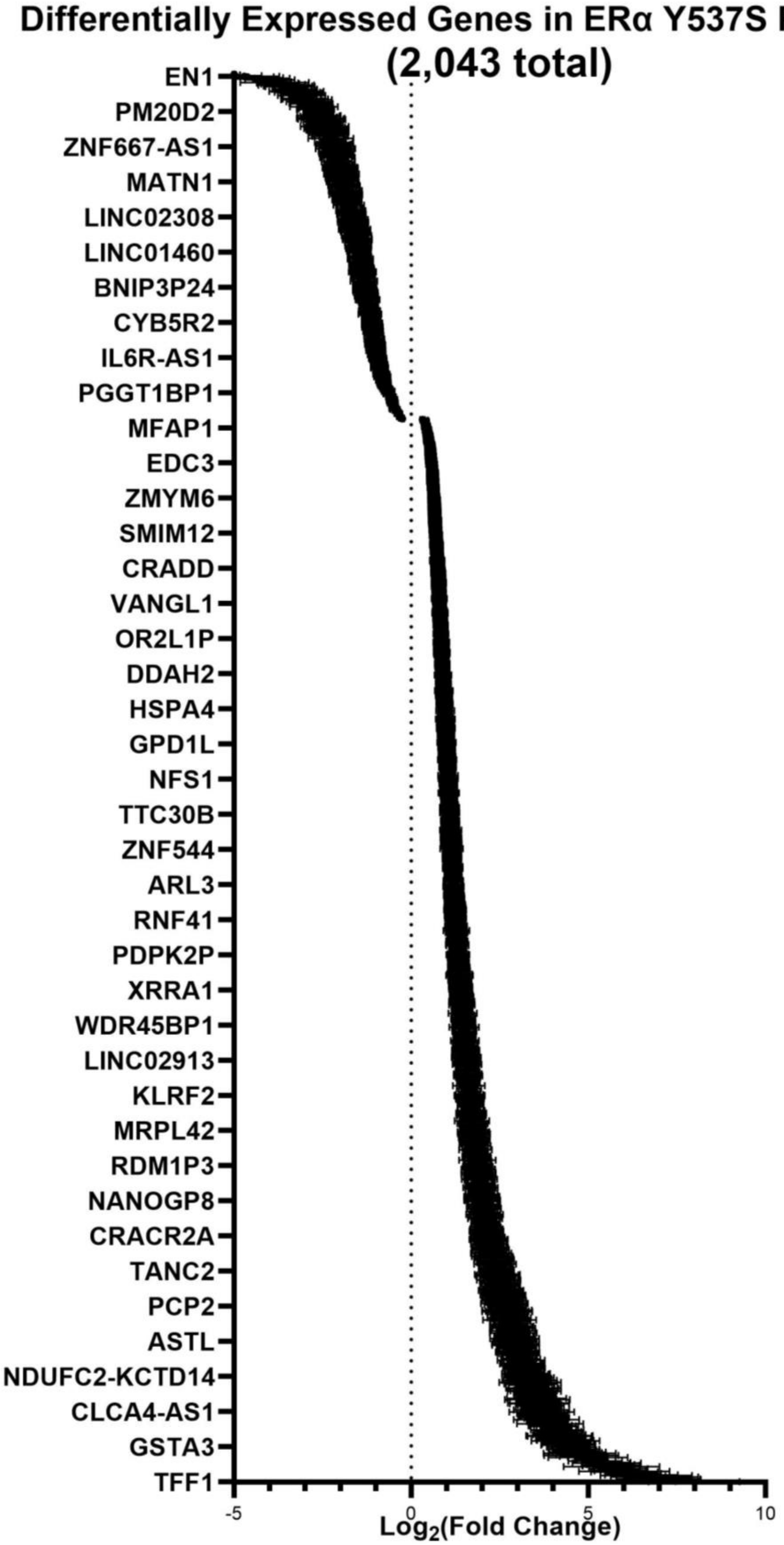

Supplementary Figure 6 Plot of log2(fold change) for differentially expressed transcripts (|log2(FC)| > 1, p-adj < 0.05) in patient tumors expressing ERα Y537S relative to ERα WT. Error bars indicate standard error of the mean (SEM).

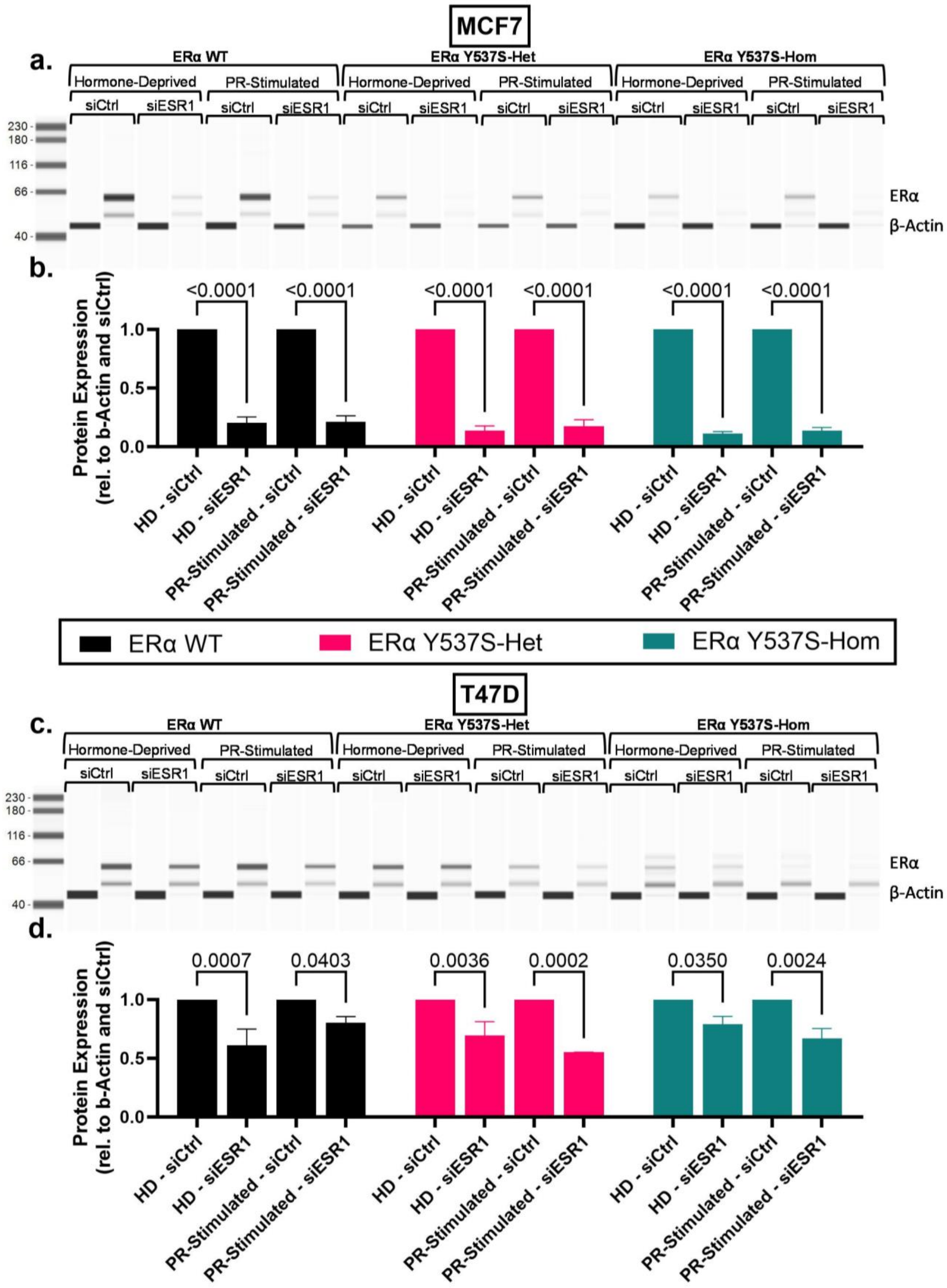

Supplementary Figure 7 Confirmation of ERα knockdown in a,b) MCF7 and c,d) T47D cell variants. **a,d.** Representative lane images from ProteinSimple WES quantification detecting ERα and β-actin loading control. **b,d.** Quantification of relative ERα expression in siCtrl and siESR1 samples, with normalization to β-actin

loading control and to paired siCtrl, in order to quantify the extent of ERα knockdown. Statistical significance was determined by two-way ANOVA with Tukey's multiple comparisons test. Data represents 3 replicates with error bars indicating standard error of the mean (SEM). P-values are indicated.

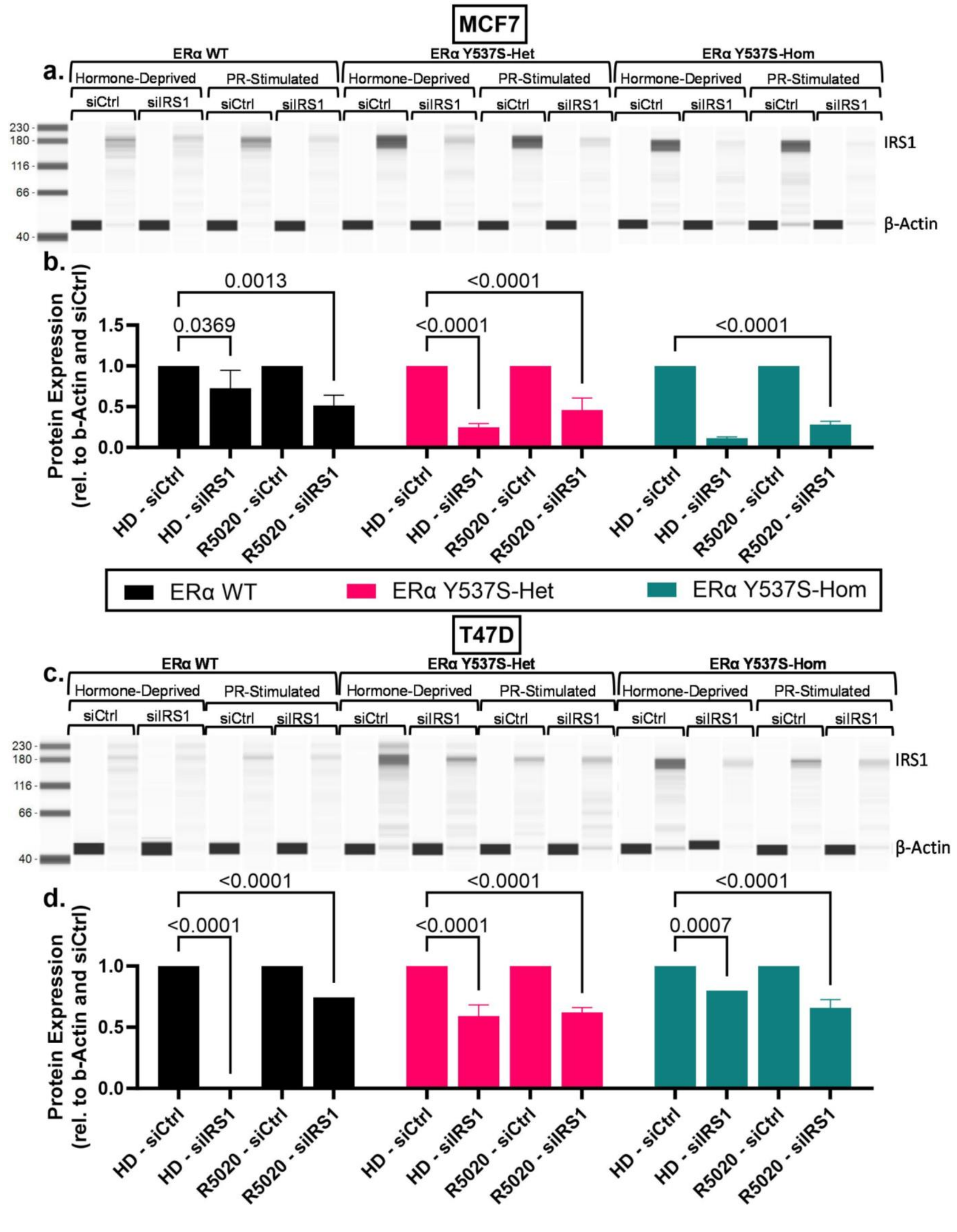

Supplementary Figure 8 Confirmation of IRS1 knockdown in **a,b)** MCF7 and **c,d)** T47D cell variants. **a,d.** Representative lane images from ProteinSimple WES quantification detecting IRS1 and β-actin loading control.

b,d. Quantification of relative IRS1 expression in siCtrl and siIRS1 samples, with normalization to β-actin loading control and to paired siCtrl, in order to quantify the extent of IRS1 knockdown. Statistical significance was determined by two-way ANOVA with Tukey's multiple comparisons test. Data represents 3 replicates with error bars indicating standard error of the mean (SEM). P-values are indicated.

Supplementary Table 1 IDs for publicly available, de-identified patient tumor datasets used in RNAseq analysis.

Patient Tumor RNAseq Dataset IDs

MET500 ERα WT	MET500 ERα Y537S	POG570 ERα WT	POG570 ERα Y537S
MO_1051-capt-SI_5093-D0VCEACXX	MO_1129-capt-SI_6222-D1RWDACXX	27216_P00991	18625_P00041
MO_1051-poly-SI_5091-D0VCEACXX	MO_1185-capt-SI_6794-H77P5ADXX	27219_P01009	19512_P00060
MO_1090-capt-SI_5612-D18NCACXX	MO_1305-capt-SI_7919-C4CRJACXX	27328_P01031	26054_P00903
MO_1107-capt-SI_5841-C19M0ACXX	MO_1355-capt-SI_8457-C4L7VACXX	27503_P01044	27329_P01026
MO_1107-poly-SI_5773-C19M0ACXX		28325_P01202	27765_P01093
MO_1126-capt-SI_6287-D1RTCACXX		30248_P01421	33154_P01932
MO_1159-capt-SI_6477-C1M1KACXX		30487_P01486	
MO_1159-poly-SI_6459-C1N0NACXX		30902_P01592	
MO_1213-capt-SI_7016-C26CMACXX		31042_P01615	
MO_1213-poly-SI_7008-C26CMACXX		31043_P01614	
MO_1237-capt-SI_7190-C245WACXX		31185_P01639	
MO_1237-poly-SI_7185-C245WACXX		31190_P01643	
MO_1239-capt-SI_7209-C245WACXX		32274_P01772	
MO_1239-poly-SI_7202-C245WACXX		32571_P01850	
MO_1247-capt-SI_7265-C25YAACXX		36621_P02129	
MO_1247-poly-SI_7235-C25YAACXX		37312_P02235	
MO_1288-capt-SI_7733-C32VAACXX		37365_P02247	
MO_1288-poly-SI_7729-C32VAACXX		38250_P02390	
MO_1289-capt-SI_7734-C32VAACXX		25483_P00631	
MO_1289-poly-SI_7730-C32VAACXX		25662_P00719	
MO_1292-capt-SI_7736-C32VAACXX		14231_A10982	
MO_1292-poly-SI_7732-C32VAACXX		15122_P00038	
MO_1298-capt-SI_7847-C3Y81ACXX		15227_T00056	
MO_1298-poly-SI_7820-C3Y81ACXX		20115_P00085	
MO_1324-capt-SI_8129-C4E6CACXX		21347_P00125	
MO_1324-poly-SI_8123-C4E6CACXX		21720_P02357	
MO_1335-capt-SI_8245-C471RANXX		22499_P00168	
MO_1335-poly-SI_8255-C471RANXX		22597_P00199	
MO_1359-capt-SI_8460-C4L7VACXX		23736_P00305	
MO_1359-poly-SI_8433-C4L7VACXX		25962_P00850	
MO_1364-capt-SI_8599-HAABDADXX		25984_P00893	
MO_1364-poly-SI_8568-HAABDADXX		27034_P00971	
MO_1411-capt-SI_9312-C5N2AANXX			
MO_1411-poly-SI_9307-HAKK6ADXX			
MO_1424-capt-SI_9381-C5N1GANXX			
MO_1424-poly-SI_9376-C5N1GANXX			
MO_1427-capt-SI_9477-C5N19ANXX			
MO_1427-poly-SI_9471-C5N19ANXX			
MO_1439-capt-SI_9741-C5N0KANXX			
MO_1439-poly-SI_9737-C5N0KANXX			
MO_1454-capt-SI_9940-C6EJUANXX			
MO_1454-poly-SI_9919-C6EJUANXX			
MO_1495-capt-SI_11221-C6UTYANXX			
MO_1495-poly-SI_11204-C6UYMANXX			
MO_1515-capt-SI_11438-HV7JNADXX			
MO_1515-poly-SI_11431-HVGHMADXX			
MO_1521-capt-SI_11539-C7GBMANXX			
MO_1521-poly-SI_11530-C7GBMANXX			
MO_1528-capt-SI_11541-C7GBMANXX			
MO_1528-poly-SI_11532-C7GBMANXX			
MO_1534-capt-SI_11904-C7F4VANXX			
MO_1536-capt-SI_11944-C7G8DANXX			

MO_1551-capt-SI_12338-C7FN8ANXX			
TP_2025-capt-SI_6023-D1EBEACXX			
TP_2025-poly-SI_6011-D1EBEACXX			
TP_2141-capt-SI_12056-H53C5ADXX			

Supplementary Table 2 Primer sequences used for ChIP-qPCR analysis of selected genomic regions.

ChIP-qPCR Primers

Primer Name	Primer Sequence (5' - 3')
**R18S FWD**	GAGTGTTCAAAGCAGGTCCAA
**R18S REV**	CCTCTAGCGGTGCAATACAAA
**IRS1 ChIP 3 FWD**	ACATCCAAGAACTCTAGCAACAA
**IRS1 ChIP 3 REV**	GCTAGGTCATTGTCACCTCAAA
**IRS1 TSS FWD**	CTGGAAGGAACAGAGGGACG
**IRS1 TSS REV**	GGACGTGAGACACTTCCTGG
**IRS1 Protein Coding FWD**	AGCTGTAGGAGAGCCTGGTA
**IRS1 Protein Coding REV**	CAACATCAACAAGCGGGCTG

Supplementary Table 3 Transcript IDs, average read counts, log2(FC), adjusted P-values, and gene information for all genes differentially expressed in cell lines expressing ERα Y537S, relative to ERα WT, filtered by Khushi et al. (2014) ERα-PR overlapping regulatory binding sites.

RNA-seq Differentially Expressed Genes - Averages of triplicate data, log2(Fold Change), p-adj < 0.05 - Filtered for ERα-PR Shared Genomic Binding Sites

MCF7 ERα Y537S-Het vs. ERα WT

Gene name	Transcript ID	Avg WT Veh	Avg WT R5020	Avg Het Veh	Avg Het R5020	log2(FC)	StdErr	P-adj	Chr	Start	End	Gene stable ID
**ACOT4**	ENST00000326303.4	1.3358	5.2223	0.5125	0.7354	-1.8518	0.4284	0.000459859	chr14	73591705	73596496	ENSG00000177465
**ACSS3**	ENST00000616449.1	4.0604	4.0431	0.0000	0.0000	-2.5152	0.4652	3.34769E-06	chr12	80936413	80940246	ENSG00000111058
**ADAMTS19**	ENST00000274487.9	8.1205	10.0361	3.6480	3.5423	-1.1233	0.3176	0.008082773	chr5	129460297	129738683	ENSG00000145808
**AREG**	ENST00000395748.8	1.3535	0.7494	10.8752	13.6347	2.8246	0.3692	2.56494E-12	chr4	74445135	74455005	ENSG00000109321
**ARMCX6**	ENST00000494624.1	0.0000	0.3271	1.4989	2.7069	1.9777	0.5148	0.002911515	chrX	101616861	101617914	ENSG00000198960
**ARRB1**	ENST00000420843.7	1.6176	3.9064	0.4708	1.8270	-1.4043	0.4613	0.033594398	chr11	75260121	75351661	ENSG00000137486
**BLK**	ENST00000526097.1	0.5275	0.3346	21.8657	18.0347	3.6048	0.3406	1.72377E-23	chr8	11560332	11564599	ENSG00000136573
**CBR3**	ENST00000290354.6	14.4483	14.6319	5.2526	6.5831	-1.3830	0.2170	1.40333E-08	chr21	36135078	36146562	ENSG00000159231
**CEBPD**	ENST00000408965.4	11.1955	16.5844	4.5320	3.5538	-1.0255	0.3204	0.022153286	chr8	47736912	47738164	ENSG00000221869
**CGN**	ENST00000271636.12	4.7970	5.0169	1.5181	1.6931	-1.3022	0.3883	0.014286958	chr1	151511386	151538692	ENSG00000143375
**CLK3**	ENST00000564353.5	6.2398	11.5363	2.9642	3.2641	-1.1698	0.3100	0.00372328	chr15	74598499	74608727	ENSG00000179335
**CNTROB**	ENST00000574430.5	0.0000	0.0000	0.7174	0.3118	-1.6510	0.5037	0.018027191	chr17	7937203	7943599	ENSG00000170037
**COL2A1**	ENST00000465743.2	2.9880	2.9176	0.4932	0.0000	-1.5375	0.5121	0.03721522	chr12	48002621	48004362	ENSG00000139219
**E2F6**	ENST00000471343.5	0.0000	0.0000	155.3029	163.0902	7.9396	0.4165	2.18669E-77	chr2	11446196	11451696	ENSG00000169016
**EGR3**	ENST00000317216.3	1.0540	0.0000	7.1503	10.0040	1.5111	0.4248	0.007553961	chr8	22687658	22693480	ENSG00000179388
**EHD4**	ENST00000569223.1	9.2649	9.7980	4.2724	1.0916	-1.4816	0.3917	0.00361305	chr15	41941603	41972552	ENSG00000103966
**FNDC3B**	ENST00000494000.1	1.8987	0.6692	4.3878	5.0459	1.5305	0.4467	0.011432283	chr3	172329073	172335814	ENSG00000075420
**FOXK2**	ENST00000335255.10	1.0897	0.0000	4.2388	8.5883	1.3249	0.4416	0.037389279	chr17	82519731	82604602	ENSG00000141568
**GIGYF2**	ENST00000463554.5	12.6764	11.2104	1.0169	0.0000	-2.7257	0.4608	2.12968E-07	chr2	232697349	232771424	ENSG00000204120
**GPC5**	ENST00000377067.9	2.7606	2.0002	0.9752	1.3033	-1.3988	0.4817	0.04746807	chr13	91398620	92867237	ENSG00000179399
**GRIK3**	ENST00000373091.8	0.0000	0.6542	3.9922	2.7069	2.1311	0.4592	0.000119401	chr1	36795526	37034515	ENSG00000163873
**KCNH7**	ENST00000477019.1	1.5815	0.9813	2.7592	5.7033	1.5306	0.3938	0.002498764	chr2	162501984	162528360	ENSG00000184611
**ONECUT1**	ENST00000305901.7	1.0720	1.0038	6.1109	3.1640	1.1117	0.3587	0.029006302	chr15	52755052	52790336	ENSG00000169856
**RPL31**	ENST00000264258.8	10.5844	9.2879	5.8675	3.5201	-1.0756	0.2884	0.004324937	chr2	101002288	101007267	ENSG00000071082
**THBS1**	ENST00000260356.6	1.8817	4.5531	11.2244	19.7947	1.3747	0.3105	0.000297762	chr15	39581078	39599466	ENSG00000137801
**TMEM45B**	ENST00000281441.8	13.7458	7.4857	5.5793	2.7962	-1.2012	0.2770	0.000437465	chr11	129815847	129860003	ENSG00000151715

MCF7 ERα Y537S-Hom vs. ERα WT

Gene name	Transcript ID	Avg WT Veh	Avg WT R5020	Avg Hom Veh	Avg Hom R5020	log2(FC)	StdErr	P-adj	Chr	Start	End	Gene stable ID
**ACOT2**	ENST00000238651.9	0.0000	0.0000	2.3982	2.1306	2.4085	0.5232	3.07421E-05	chr14	73569058	73575653	ENSG00000119673
**ACOT2**	ENST00000538782.2	0.0000	0.0000	5.7451	6.5481	3.9774	0.4910	1.08073E-14	chr14	73567619	73575653	ENSG00000119673
**ACOT6**	ENST00000381139.1	1.5809	0.6542	0.6496	0.0000	-2.3079	0.4848	1.49471E-05	chr14	73616843	73619888	ENSG00000205669
**AKAP9**	ENST00000394534.6	3.5149	5.5645	1.5100	0.0000	-1.1939	0.4475	0.027195958	chr7	92070137	92109061	ENSG00000127914
**AREG**	ENST00000395748.8	1.3535	0.7494	41.6130	22.1357	4.1552	0.3608	4.64013E-29	chr4	74445135	74455005	ENSG00000109321
**ARID1B**	ENST00000346085.10	0.8266	0.3346	2.1617	2.6780	1.0992	0.4341	0.038104251	chr6	156776019	157210779	ENSG00000049618
**ARMCX1**	ENST00000372829.8	0.0000	0.0000	3.1020	3.4661	2.4702	0.5233	1.79979E-05	chrX	101550546	101554700	ENSG00000126947
**ARMCX6**	ENST00000494624.1	0.0000	0.3271	1.0106	1.2834	1.3510	0.5184	0.031718644	chrX	101616861	101617914	ENSG00000198960
**ARRDC4**	ENST00000268042.7	3.7790	5.8991	3.1723	1.0358	-1.2177	0.4258	0.016275243	chr15	97960702	97973833	ENSG00000140450
**ASAP1**	ENST00000520625.1	3.3058	1.7383	12.4721	11.0399	2.2251	0.3376	5.75828E-10	chr8	130360688	130401915	ENSG00000153317
**ATXN7**	ENST00000487717.5	1.3541	7.0696	14.2814	10.2901	1.6560	0.2884	9.57801E-08	chr3	63911928	64000207	ENSG00000163635
**AVIL**	ENST00000546952.1	34.8384	30.7438	12.8471	15.7229	-1.2610	0.1831	8.27566E-11	chr12	57797375	57800653	ENSG00000135407
**AVIL**	ENST00000551248.1	3.4632	7.4857	1.8007	0.7429	-1.7664	0.4287	0.000236331	chr12	57797777	57801417	ENSG00000135407
**BAIAP2**	ENST00000321300.10	9.3873	8.2991	4.3470	1.0111	-1.6026	0.3225	5.54751E-06	chr17	81035161	81117432	ENSG00000175866
**BCAS1**	ENST00000448484.5	1.3181	0.0000	3.3108	4.2159	1.8787	0.4840	0.000596812	chr20	53936776	54028700	ENSG00000064787
**BCAS4**	ENST00000371608.6	1.0897	1.4037	2.9679	2.7377	1.2700	0.4679	0.023969677	chr20	50794993	50882676	ENSG00000124243
**BCL10**	ENST00000648566.1	0.0000	1.0766	0.3429	2.0785	1.8197	0.5208	0.00238466	chr1	85265775	85276632	ENSG00000142867

**BCL2L11**	ENST00000432179.1	1.3715	0.7494	4.7185	5.5233	1.5184	0.4290	0.002042586	chr2	111119377	111124112	ENSG00000153094
**BMP7**	ENST00000395863.8	0.5272	0.9888	5.1914	3.3894	1.9090	0.4437	0.00011284	chr20	57168752	57266641	ENSG00000101144
**BNIP3**	ENST00000540159.3	3.1814	6.2112	17.1821	13.4532	1.2021	0.2622	3.33588E-05	chr10	131968227	131981923	ENSG00000176171
**BRWD1**	ENST00000333229.6	4.5699	7.2652	0.0000	1.5654	-1.6931	0.3997	0.000148591	chr21	39184175	39313819	ENSG00000185658
**C19orf33**	ENST00000588605.5	0.0000	1.0766	5.7952	7.4029	1.3629	0.4277	0.006437045	chr19	38304163	38305006	ENSG00000167644
**C2orf49**	ENST00000258457.7	1.6349	0.6617	0.3951	0.4953	-1.3505	0.4856	0.02015496	chr2	105337539	105349211	ENSG00000135974
**CACNG4**	ENST00000262138.4	0.0000	0.0000	39.5959	32.0573	5.1783	0.4233	1.07113E-32	chr17	66964706	67033398	ENSG00000075461
**CALM2**	ENST00000668667.1	1.0900	0.0000	2.8454	2.4969	1.2143	0.4978	0.047101729	chr2	47160204	47175539	ENSG00000143933
**CAPN7**	ENST00000463417.5	2.1984	2.8148	3.5312	1.0358	-1.0863	0.4137	0.030240093	chr3	15245886	15252302	ENSG00000131375
**CCNI**	ENST00000515790.1	2.3915	5.3740	0.0000	0.0000	-2.9969	0.4947	1.5396E-08	chr4	77066164	77075620	ENSG00000118816
**CDH13**	ENST00000567109.6	105.6746	105.3866	361.9670	343.3517	2.2143	0.1336	1.28478E-59	chr16	82626968	83800640	ENSG00000140945
**CDK6**	ENST00000265734.8	0.0000	0.0000	1.3876	2.2142	1.8748	0.5107	0.001293828	chr7	92604920	92833917	ENSG00000105810
**CEBPB**	ENST00000303004.5	12.4307	11.4560	49.0640	41.7747	1.4843	0.1958	5.89946E-13	chr20	50190829	50192668	ENSG00000172216
**CGN**	ENST00000502442.1	0.0000	0.3346	5.2681	4.3270	2.1083	0.4780	7.15456E-05	chr1	151510935	151519029	ENSG00000143375
**CHDH**	ENST00000315251.11	105.9708	103.0949	25.7876	18.8508	-2.2015	0.1703	1.80071E-36	chr3	53812334	53846419	ENSG00000016391
**CHI3L2**	ENST00000445067.6	0.8086	1.3234	4.5779	2.8626	1.1065	0.4565	0.04875356	chr1	111200770	111243439	ENSG00000064886
**CHKA**	ENST00000265689.9	6.6977	10.7718	0.6858	0.4953	-3.2454	0.3755	1.24946E-16	chr11	68052858	68121388	ENSG00000110721
**CKAP4**	ENST00000378026.5	6.6416	8.5622	11.1026	12.0681	1.0307	0.2595	0.000421681	chr12	106237880	106248020	ENSG00000136026
**CLDN9**	ENST00000445369.3	0.0000	0.0000	0.9925	2.6602	1.6548	0.5227	0.006836928	chr16	3012922	3014505	ENSG00000213937
**CMTM6**	ENST00000205636.4	7.4699	4.4729	1.3173	1.7856	-1.3859	0.3333	0.000203012	chr3	32481311	32502852	ENSG00000091317
**CMTM7**	ENST00000487007.1	676.8133	641.1126	261.5378	295.4236	-1.1892	0.0648	5.33891E-73	chr3	32449514	32483067	ENSG00000153551
**CMYA5**	ENST00000446378.3	0.0000	0.6542	2.5568	3.8043	1.6563	0.5267	0.007288952	chr5	79689835	79800222	ENSG00000164309
**CNTROB**	ENST00000571632.5	0.0000	0.6542	0.0000	0.2929	-1.4794	0.4996	0.012371218	chr17	7934528	7949578	ENSG00000170037
**CNTROB**	ENST00000573862.1	0.7906	0.6617	5.7632	4.0692	-1.2492	0.4466	0.019275112	chr17	7945330	7947722	ENSG00000170037
**CNTROB**	ENST00000574430.5	0.0000	0.0000	0.7199	1.5517	-1.3856	0.4998	0.020568296	chr17	7937203	7943599	ENSG00000170037
**CNTROB**	ENST00000576922.5	0.2638	1.0766	1.6783	1.8377	-1.2708	0.4645	0.02266576	chr17	7937202	7947685	ENSG00000170037
**COL18A1**	ENST00000651438.1	0.5629	0.3346	2.2139	2.3920	1.4333	0.5070	0.017790243	chr21	45405164	45513720	ENSG00000182871
**DDIT4**	ENST00000307365.4	1.3181	0.6692	2.1075	4.0417	1.3285	0.4525	0.013227091	chr10	72273923	72276036	ENSG00000168209
**DLX1**	ENST00000409492.1	0.0000	0.0000	0.6496	4.0204	1.8849	0.5264	0.001774735	chr2	172085556	172087138	ENSG00000144355
**DLX1**	ENST00000550686.1	0.0000	0.3271	0.6677	2.9571	1.6066	0.5270	0.009657051	chr2	172087366	172088373	ENSG00000144355
**DSP**	ENST00000379802.8	12.7454	18.9254	32.8733	36.2086	1.0029	0.2574	0.000563596	chr6	7541670	7586714	ENSG00000096696
**EBNA1BP2**	ENST00000461557.2	1.3538	2.6619	6.5874	6.6600	1.8470	0.3695	4.83399E-06	chr1	43171609	43270936	ENSG00000117395
**ECI1**	ENST00000301729.9	0.7906	3.1645	4.7526	6.7896	1.7721	0.3899	3.97752E-05	chr16	2239401	2251587	ENSG00000167969
**EGR3**	ENST00000317216.3	1.0540	0.0000	8.8471	5.4190	1.0811	0.4265	0.037876847	chr8	22687658	22693480	ENSG00000179388
**EHD4**	ENST00000220325.9	8.6476	14.5790	2.8294	1.7993	-1.0328	0.3500	0.012701351	chr15	41895932	41972557	ENSG00000103966
**EHD4**	ENST00000569223.1	9.2649	9.7980	0.3429	1.5201	-2.4775	0.4205	4.06934E-08	chr15	41941603	41972552	ENSG00000103966
**EN2**	ENST00000297375.4	5.7137	3.0617	1.7485	2.2211	-1.1287	0.4023	0.018851802	chr7	155458128	155464831	ENSG00000164778
**EPHA4**	ENST00000409938.5	0.0000	0.0000	2.2299	2.6465	2.4460	0.5176	1.76255E-05	chr2	221425900	221574202	ENSG00000116106
**EPHA4**	ENST00000434266.1	0.0000	0.6542	6.0072	5.1955	2.9105	0.4748	1.00738E-08	chr2	221564278	221574158	ENSG00000116106
**ERRFI1**	ENST00000474874.5	2.1077	2.0879	0.0000	0.0000	-1.4584	0.5256	0.020459986	chr1	8004403	8026283	ENSG00000116285
**ESR1**	ENST00000338799.9	0.2814	0.0000	2.0191	0.8334	1.4028	0.5276	0.027853021	chr6	151804430	152099053	ENSG00000091831
**FNDC3B**	ENST00000494000.1	1.8987	0.6692	0.0000	0.0000	-1.5966	0.5051	0.00693284	chr3	172329073	172335814	ENSG00000075420
**GIGYF2**	ENST00000463554.5	12.6764	11.2104	2.5206	0.5858	-2.1693	0.4449	8.71092E-06	chr2	232697349	232771424	ENSG00000204120
**GPC5**	ENST00000377067.9	2.7606	2.0002	0.0000	0.0000	-2.8082	0.5046	2.5625E-07	chr13	91398620	92867237	ENSG00000179399
**GRIK3**	ENST00000373091.8	0.0000	0.6542	8.9792	6.2628	3.1151	0.4481	5.27765E-11	chr1	36795526	37034515	ENSG00000163873
**HDAC11**	ENST00000433119.5	0.5629	1.0841	5.4906	2.9256	1.3236	0.4836	0.02259171	chr3	13479723	13505334	ENSG00000163517
**HSPB1**	ENST00000447574.1	0.0000	0.0000	2.0392	2.3330	1.6408	0.5262	0.007877338	chr7	76302687	76304260	ENSG00000106211
**IGFBP5**	ENST00000233813.5	85.1076	119.3331	15.2770	9.7847	-2.8903	0.2565	7.59561E-28	chr2	216672104	216695549	ENSG00000115461
**ITIH6**	ENST00000218436.7	0.0000	0.0000	9.5404	7.9373	3.7328	0.4958	8.77459E-13	chrX	54748917	54798255	ENSG00000102313
**KCNAB2**	ENST00000378111.6	1.3535	0.6542	0.0000	0.0000	-1.4467	0.5245	0.021392093	chr1	5991319	6090446	ENSG00000069424
**KCNS2**	ENST00000287042.5	0.2634	0.0000	1.5281	2.6533	1.5113	0.5250	0.015520753	chr8	98426957	98432853	ENSG00000156486
**KLHL11**	ENST00000319121.4	1.1077	2.7346	0.7902	0.5858	-1.4743	0.4899	0.010801448	chr17	41848517	41865423	ENSG00000178502
**LETM1**	ENST00000302787.3	49.9933	64.0040	10.4912	8.6652	-2.1877	0.1816	9.90391E-32	chr4	1811478	1856156	ENSG00000168924
**LGALS2**	ENST00000215886.6	10.4460	11.7681	2.1255	1.5763	-2.7335	0.3637	9.57688E-13	chr22	37570247	37580087	ENSG00000100079
**LRRC34**	ENST00000522596.6	16.1366	16.6634	8.7045	8.1005	-1.1014	0.2114	1.67233E-06	chr3	169793472	169812690	ENSG00000171757
**LRRC4**	ENST00000249363.4	0.5452	0.0000	2.3640	1.6668	1.4512	0.5109	0.017157181	chr7	128027070	128031437	ENSG00000128594
**MAN1A2**	ENST00000356554.7	10.5837	7.7326	26.3882	25.5893	1.3878	0.2493	2.5424E-07	chr1	117367448	117528872	ENSG00000198162

**MAP3K1**	ENST00000399503.4	1.6169	0.3271	3.3545	5.3636	1.5486	0.4242	0.001389286	chr5	56815548	56896152	ENSG00000095015
**MARCKS**	ENST00000612661.2	21.9410	17.5270	110.8259	104.1928	2.4142	0.1772	2.03809E-40	chr6	113857344	113863475	ENSG00000277443
**MED13L**	ENST00000548743.2	1.6709	1.6356	0.0000	0.0000	-1.5336	0.5279	0.014396804	chr12	115991679	116276759	ENSG00000123066
**MED13L**	ENST00000650375.1	1.3535	2.5027	0.0000	0.0000	-2.2721	0.5136	6.7456E-05	chr12	116237498	116276295	ENSG00000123066
**NDUFB9**	ENST00000517830.1	4.3238	9.1927	48.1089	51.4191	2.9970	0.2136	7.3485E-43	chr8	124549773	124552318	ENSG00000147684
**NDUFB9**	ENST00000522532.6	4.7637	3.1495	34.2236	20.8423	2.6115	0.2575	1.1787E-22	chr8	124539139	124580648	ENSG00000147684
**NUAK1**	ENST00000261402.6	0.5452	3.8839	5.7110	5.4609	1.1540	0.4247	0.023822259	chr12	106063339	106140033	ENSG00000074590
**ONECUT1**	ENST00000305901.7	1.0720	1.0038	13.9184	6.1126	1.6186	0.3502	2.82584E-05	chr15	52755052	52790336	ENSG00000169856
**PGR**	ENST00000325455.10	0.0000	0.0000	8.7663	9.6975	2.1964	0.4296	2.74075E-06	chr11	101029623	101129813	ENSG00000082175
**PIK3R1**	ENST00000521381.6	0.2634	0.6617	2.7752	2.9187	1.1899	0.4849	0.045611304	chr5	68215755	68301821	ENSG00000145675
**PIM3**	ENST00000360612.5	2.1614	2.4075	8.3295	4.1913	1.0162	0.3283	0.008426218	chr22	49960771	49964072	ENSG00000198355
**PLEKHH2**	ENST00000405000.6	0.0000	0.0000	9.3807	9.2681	3.8199	0.4969	2.71709E-13	chr2	43637307	43765792	ENSG00000152527
**PPFIA1**	ENST00000389547.7	2.3908	2.4000	0.6677	2.4166	-1.1052	0.4064	0.023687141	chr11	70270716	70378580	ENSG00000131626
**PPFIA1**	ENST00000530548.5	17.2797	20.5698	1.8188	6.9254	-1.7726	0.2426	4.39528E-12	chr11	70364341	70384394	ENSG00000131626
**PPP1R3B**	ENST00000310455.4	3.2712	1.8335	1.8529	1.0879	-1.3698	0.5050	0.024102787	chr8	9136254	9150658	ENSG00000173281
**PREX1**	ENST00000371941.4	18.1117	21.4947	8.6022	5.8581	-1.0134	0.2760	0.001293828	chr20	48624251	48827999	ENSG00000124126
**RBPMS**	ENST00000520916.1	0.5629	1.6506	3.8805	1.7993	1.1796	0.4725	0.041409126	chr8	30564049	30570994	ENSG00000157110
**SHANK2**	ENST00000445654.2	0.5268	0.0000	7.9686	3.3022	2.4513	0.4899	4.71696E-06	chr11	70566386	70659910	ENSG00000162105
**SHC3**	ENST00000375835.9	6.8931	4.1458	0.3429	0.2545	-2.5879	0.4707	3.68395E-07	chr9	89005770	89178818	ENSG00000148082
**SLFNL1**	ENST00000359345.5	3.1808	0.9888	1.3173	1.0948	-1.8763	0.4332	0.000100214	chr1	41015596	41023237	ENSG00000171790
**SNTB1**	ENST00000395601.7	2.1257	2.0729	0.7380	1.3287	-1.3325	0.4239	0.00731857	chr8	120535744	120813273	ENSG00000172164
**SNTB1**	ENST00000520717.1	2.1804	2.7496	0.3951	0.8019	-1.3546	0.4715	0.015732672	chr8	120811758	120812852	ENSG00000172164
**TAX1BP1**	ENST00000460059.1	4.7803	6.9756	30.1684	30.9073	2.2621	0.2688	8.36936E-16	chr7	27828699	27833151	ENSG00000106052
**TAX1BP1**	ENST00000488564.2	4.2705	1.7308	13.5947	11.0605	1.9255	0.3371	1.12819E-07	chr7	27827800	27844564	ENSG00000106052
**TBC1D16**	ENST00000310924.7	0.2634	1.9927	4.5779	5.3458	1.3333	0.3992	0.003984595	chr17	79932342	80035872	ENSG00000167291
**TBC1D30**	ENST00000434563.3	0.0000	0.0000	2.6708	0.7429	1.3348	0.5113	0.031353852	chr12	64759520	64779318	ENSG00000111490
**TCEA3**	ENST00000450454.7	3.7073	0.9888	0.0000	0.0000	-2.4448	0.4950	6.41317E-06	chr1	23380908	23424748	ENSG00000204219
**THBS1**	ENST00000260356.6	1.8817	4.5531	64.9251	54.5947	3.0747	0.3012	6.1117E-23	chr15	39581078	39599466	ENSG00000137801
**THBS1**	ENST00000466755.1	0.0000	0.0000	0.6677	1.8446	1.8091	0.5185	0.002423013	chr15	39587451	39588472	ENSG00000137801
**THBS1**	ENST00000490247.1	0.0000	0.0000	3.0136	2.0017	1.6651	0.5277	0.007047706	chr15	39588847	39589843	ENSG00000137801
**THBS1**	ENST00000560894.1	0.2638	0.0000	3.7878	7.0991	2.9364	0.4955	3.34614E-08	chr15	39590927	39591618	ENSG00000137801
**THSD4**	ENST00000355327.7	0.5449	0.0000	2.4162	2.3330	1.8990	0.5038	0.000907496	chr15	71096951	71783383	ENSG00000187720
**TLE1**	ENST00000376499.8	4.0254	4.0506	10.5936	10.7923	1.5702	0.3207	7.90401E-06	chr9	81583682	81689547	ENSG00000196781
**TMEM45B**	ENST00000281441.8	13.7458	7.4857	3.5834	2.9640	-1.1070	0.2710	0.000271947	chr11	129815847	129860003	ENSG00000151715
**TNFRSF21**	ENST00000296861.2	0.5629	0.9813	8.0047	4.8752	2.1105	0.4760	6.46641E-05	chr6	47231531	47309905	ENSG00000146072
**TSHZ2**	ENST00000371497.10	4.0614	6.0445	20.1107	31.4548	2.3513	0.3158	1.61043E-12	chr20	52972357	53495330	ENSG00000182463
**TSPAN14**	ENST00000265450.5	4.0974	2.8298	0.3248	0.0000	-2.0836	0.5025	0.000212664	chr10	80515466	80519635	ENSG00000108219
**TTC39A**	ENST00000262676.9	11.7091	14.0879	37.4730	41.5785	1.1673	0.1665	3.504E-11	chr1	51297217	51331346	ENSG00000085831
**USP3**	ENST00000561381.5	1.0904	1.9777	5.8591	5.4226	1.6301	0.4413	0.00119288	chr15	63587969	63591003	ENSG00000140455
**VMP1**	ENST00000592790.1	0.0000	0.3271	0.7902	2.3014	1.8473	0.5080	0.00145838	chr17	59837930	59842255	ENSG00000062716
**ZNF710**	ENST00000268154.9	66.3913	88.0217	30.0920	37.0969	-1.1435	0.1220	1.88226E-19	chr15	90001323	90082191	ENSG00000140548

MCF7 ERα Y537S-Hom vs. ERα Y537S-Het

Gene name	Transcript ID	Avg Het Veh	Avg Het R5020	Avg Hom Veh	Avg Hom R5020	log2(FC)	StdErr	P-adj	Chr	Start	End	Gene stable ID
**ACOT2**	ENST00000238651.9	0.0000	0.0000	2.3982	2.1306	2.4285	0.5232	8.20684E-05	chr14	73569058	73575653	ENSG00000119673
**ACOT2**	ENST00000538782.2	0.0000	0.0000	5.7451	6.5481	3.9982	0.4905	2.31649E-14	chr14	73567619	73575653	ENSG00000119673
**ACOT4**	ENST00000326303.4	0.5125	0.7354	1.3876	3.8709	1.3977	0.4330	0.017956738	chr14	73591705	73596496	ENSG00000177465
**ACOT6**	ENST00000381139.1	3.2717	2.6287	0.6496	0.0000	-2.1014	0.4864	0.000329143	chr14	73616843	73619888	ENSG00000205669
**ACOX1**	ENST00000293217.10	4.5544	1.8048	0.7380	1.2656	-1.4211	0.4383	0.017128979	chr17	75941506	75979166	ENSG00000161533
**ACSF3**	ENST00000393145.5	3.0667	3.3084	10.5574	11.3636	1.3161	0.3622	0.004694778	chr16	89147566	89154586	ENSG00000176715
**ACSS3**	ENST00000616449.1	0.0000	0.0000	3.4609	5.8237	2.9181	0.4581	7.56254E-09	chr12	80936413	80940246	ENSG00000111058
**ACTR6**	ENST00000553038.5	6.8316	7.1404	1.1330	0.9974	-1.4334	0.3640	0.001528672	chr12	100199864	100241865	ENSG00000075089
**ADAMTS19**	ENST00000274487.9	3.6480	3.5423	8.4159	5.9417	1.0783	0.3154	0.009817352	chr5	129460297	129738683	ENSG00000145808
**ADAP2**	ENST00000583688.1	4.8122	7.0518	2.7410	2.0050	-1.1149	0.3707	0.034792162	chr17	30906343	30921760	ENSG00000184060
**AKAP9**	ENST00000394534.6	2.6375	5.9604	1.5100	0.0000	-1.4235	0.4436	0.019004674	chr7	92070137	92109061	ENSG00000127914
**ANKRD11**	ENST00000644045.1	0.2770	0.7354	3.2947	2.9846	1.5884	0.4967	0.019705351	chr16	89301323	89490319	ENSG00000167522

**AREG**	ENST00000395748.8	10.8752	13.6347	41.6130	22.1357	1.3306	0.2702	2.21092E-05	chr4	74445135	74455005	ENSG00000109321
**ARHGEF17**	ENST00000263674.4	2.0530	1.9493	6.1583	4.6934	1.2895	0.4198	0.028813858	chr11	73308275	73369388	ENSG00000110237
**ARMCX1**	ENST00000372829.8	0.0000	0.0000	3.1020	3.4661	2.4902	0.5233	4.80897E-05	chrX	101550546	101554700	ENSG00000126947
**ASAP1**	ENST00000520625.1	1.0361	0.9914	12.4721	11.0399	2.6874	0.3551	2.09572E-12	chr8	130360688	130401915	ENSG00000153317
**ATXN7**	ENST00000487717.5	3.4014	5.7370	14.2814	10.2901	1.7491	0.2892	5.34826E-08	chr3	63911928	64000207	ENSG00000163635
**BAIAP2**	ENST00000321300.10	15.6266	20.6089	4.3470	1.0111	-2.4758	0.3134	1.6807E-13	chr17	81035161	81117432	ENSG00000175866
**BBIP1**	ENST00000422050.1	8.2552	4.3778	3.4971	0.7882	-1.4956	0.3669	0.000895747	chr10	110906478	110919167	ENSG00000214413
**BBIP1**	ENST00000605265.1	38.1835	39.9037	12.3220	9.7742	-1.5779	0.2051	8.22132E-13	chr10	110900233	110904945	ENSG00000214413
**BCL2L11**	ENST00000432179.1	3.8754	1.4150	4.7185	5.5233	1.2438	0.4198	0.039588555	chr2	111119377	111124112	ENSG00000153094
**BLK**	ENST00000526097.1	21.8657	18.0347	1.1150	0.2545	-4.4665	0.3826	2.55761E-29	chr8	11560332	11564599	ENSG00000136573
**BMP7**	ENST00000395863.8	0.7703	0.6796	5.1914	3.3894	2.1229	0.4471	5.03705E-05	chr20	57168752	57266641	ENSG00000101144
**BNIP3**	ENST00000540159.3	6.8844	6.4389	17.1821	13.4532	1.2529	0.2618	4.24082E-05	chr10	131968227	131981923	ENSG00000176171
**BRI3**	ENST00000539286.5	84.6725	89.2720	42.6951	46.6181	-1.0451	0.0946	3.00817E-26	chr7	98281666	98292963	ENSG00000164713
**BRWD1**	ENST00000333229.6	4.7786	4.7010	0.0000	1.5654	-1.5405	0.4012	0.002221352	chr21	39184175	39313819	ENSG00000185658
**C19orf33**	ENST00000588605.5	0.5541	0.7797	5.7952	7.4029	2.1368	0.4453	4.00314E-05	chr19	38304163	38305006	ENSG00000167644
**CACNG4**	ENST00000262138.4	0.0000	0.0000	39.5959	32.0573	5.6928	0.4414	8.91273E-36	chr17	66964706	67033398	ENSG00000075461
**CALM2**	ENST00000668667.1	0.2354	0.9355	2.8454	2.4969	1.5418	0.5011	0.02845024	chr2	47160204	47175539	ENSG00000143933
**CBR3**	ENST00000290354.6	5.2526	6.5831	19.6028	17.7027	1.4352	0.2141	8.94334E-10	chr21	36135078	36146562	ENSG00000159231
**CCDC88C**	ENST00000334448.5	0.7062	1.7711	0.0000	0.2929	-1.6196	0.5157	0.023427311	chr14	91273468	91283623	ENSG00000015133
**CCNI**	ENST00000515790.1	3.2300	2.8627	0.0000	0.0000	-2.3639	0.4992	5.36372E-05	chr4	77066164	77075620	ENSG00000118816
**CDH13**	ENST00000567109.6	71.7092	53.5944	361.9670	343.3517	2.6425	0.1364	9.77108E-81	chr16	82626968	83800640	ENSG00000140945
**CDK6**	ENST00000265734.8	0.4820	0.0000	1.3876	2.2142	1.6363	0.5085	0.01850779	chr7	92604920	92833917	ENSG00000105810
**CEBPB**	ENST00000303004.5	14.7650	23.1586	49.0640	41.7747	1.3254	0.1927	2.78921E-10	chr20	50190829	50192668	ENSG00000172216
**CEBPD**	ENST00000408965.4	4.5320	3.5538	9.7086	8.4662	1.0975	0.3168	0.008418812	chr8	47736912	47738164	ENSG00000221869
**CGN**	ENST00000271636.12	1.5181	1.6931	8.9068	3.6055	1.5191	0.3822	0.001328792	chr1	151511386	151538692	ENSG00000143375
**CGN**	ENST00000502442.1	0.7479	1.4708	5.2681	4.3270	1.7071	0.4659	0.004222911	chr1	151510935	151519029	ENSG00000143375
**CHDH**	ENST00000315251.11	74.1569	86.9756	25.7876	18.8508	-1.7395	0.1721	5.06516E-22	chr3	53812334	53846419	ENSG00000016391
**CHKA**	ENST00000265689.9	10.8430	11.5076	0.6858	0.4953	-3.2733	0.3745	1.81442E-16	chr11	68052858	68121388	ENSG00000110721
**CHPT1**	ENST00000546873.1	3.5518	1.8048	0.3429	0.5474	-1.8549	0.4874	0.002513776	chr12	101726322	101744140	ENSG00000111666
**CLDN9**	ENST00000445369.3	0.0000	0.0000	0.9925	2.6602	1.6708	0.5227	0.019775166	chr16	3012922	3014505	ENSG00000213937
**CMTM6**	ENST00000205636.4	5.7314	7.3521	1.3173	1.7856	-1.4497	0.3312	0.000260307	chr3	32481311	32502852	ENSG00000091317
**CMTM7**	ENST00000487007.1	623.7505	601.7619	261.5378	295.4236	-1.0775	0.0650	5.17063E-59	chr3	32449514	32483067	ENSG00000153551
**CMYA5**	ENST00000446378.3	0.0000	0.0000	2.5568	3.8043	1.7969	0.5266	0.010013878	chr5	79689835	79800222	ENSG00000164309
**COL18A1**	ENST00000473212.1	5.1228	4.1438	2.0733	1.8830	-1.2666	0.3266	0.001916152	chr21	45506018	45512567	ENSG00000182871
**COL18A1**	ENST00000651438.1	0.0000	0.9355	2.2139	2.3920	1.8689	0.5110	0.004318343	chr21	45405164	45513720	ENSG00000182871
**DLX1**	ENST00000409492.1	0.0000	0.0000	0.6496	4.0204	1.9019	0.5264	0.005044779	chr2	172085556	172087138	ENSG00000144355
**E2F6**	ENST00000471343.5	155.3029	163.0902	0.0000	0.0000	-8.0530	0.4159	1.32124E-80	chr2	11446196	11451696	ENSG00000169016
**E2F6**	ENST00000498701.1	3.2443	0.7576	0.0000	0.0000	-1.8111	0.5270	0.009225521	chr2	11457010	11466158	ENSG00000169016
**ECI1**	ENST00000301729.9	1.7150	3.1524	4.7526	6.7896	1.2936	0.3728	0.008262349	chr16	2239401	2251587	ENSG00000167969
**EPHA4**	ENST00000409938.5	0.2354	0.0000	2.2299	2.6465	2.2158	0.5173	0.000385052	chr2	221425900	221574202	ENSG00000116106
**EPHA4**	ENST00000434266.1	0.0000	0.3677	6.0072	5.1955	3.1998	0.4786	9.9303E-10	chr2	221564278	221574158	ENSG00000116106
**FBXO2**	ENST00000465901.1	0.0000	0.0000	1.7646	4.1913	1.5402	0.5003	0.028294466	chr1	11650546	11655785	ENSG00000116661
**FNDC3B**	ENST00000494000.1	4.3878	5.0459	0.0000	0.0000	-3.1272	0.4855	4.80027E-09	chr3	172329073	172335814	ENSG00000075420
**FOXK2**	ENST00000335255.10	4.2388	8.5883	1.0106	1.2725	-2.1877	0.4573	4.29157E-05	chr17	82519731	82604602	ENSG00000141568
**IGFBP5**	ENST00000233813.5	88.3421	122.9572	15.2770	9.7847	-2.9172	0.2566	8.18841E-28	chr2	216672104	216695549	ENSG00000115461
**ITGB1**	ENST00000439974.3	0.7703	0.7797	3.5131	4.5747	1.5020	0.4446	0.011197844	chr10	32958485	32960503	ENSG00000150093
**ITIH6**	ENST00000218436.7	0.0000	0.7354	9.5404	7.9373	3.4753	0.4923	8.0841E-11	chrX	54748917	54798255	ENSG00000102313
**KCNS2**	ENST00000287042.5	0.7591	0.0000	1.5281	2.6533	1.5388	0.5248	0.043021174	chr8	98426957	98432853	ENSG00000156486
**LETM1**	ENST00000302787.3	49.9237	59.0399	10.4912	8.6652	-2.1806	0.1816	5.07104E-31	chr4	1811478	1856156	ENSG00000168924
**LGALS2**	ENST00000215886.6	12.7612	13.0112	2.1255	1.5763	-2.3330	0.3668	7.96498E-09	chr22	37570247	37580087	ENSG00000100079
**LRRC34**	ENST00000522596.6	18.0734	21.2536	8.7045	8.1005	-1.2601	0.2083	5.31338E-08	chr3	169793472	169812690	ENSG00000171757
**MAN1A2**	ENST00000356554.7	7.1327	10.1030	26.3882	25.5893	1.2019	0.2449	2.39557E-05	chr1	117367448	117528872	ENSG00000198162
**MAP3K1**	ENST00000399503.4	1.2411	0.3677	3.3545	5.3636	2.5653	0.4539	5.12681E-07	chr5	56815548	56896152	ENSG00000095015
**MARCKS**	ENST00000612661.2	21.6481	21.1103	110.8259	104.1928	2.1553	0.1730	2.19729E-33	chr6	113857344	113863475	ENSG00000277443
**NDUFB9**	ENST00000517830.1	6.3048	8.2994	48.1089	51.4191	2.5570	0.1946	3.92762E-37	chr8	124549773	124552318	ENSG00000147684
**NDUFB9**	ENST00000522532.6	2.3493	6.0267	34.2236	20.8423	2.7220	0.2586	7.46978E-24	chr8	124539139	124580648	ENSG00000147684
**NUAK1**	ENST00000261402.6	0.5236	1.4371	5.7110	5.4609	1.6046	0.4350	0.003869205	chr12	106063339	106140033	ENSG00000074590

**PGR**	ENST00000325455.10	0.9945	0.3118	8.7663	9.6975	2.0168	0.4219	4.34832E-05	chr11	101029623	101129813	ENSG00000082175
**PIK3R1**	ENST00000521381.6	0.7062	0.0000	2.7752	2.9187	1.5567	0.4900	0.02091931	chr5	68215755	68301821	ENSG00000145675
**PLEKHH2**	ENST00000405000.6	0.0000	0.0000	9.3807	9.2681	3.8385	0.4962	5.92212E-13	chr2	43637307	43765792	ENSG00000152527
**PPFIA1**	ENST00000389547.7	5.1805	3.9658	0.6677	2.4166	-1.5115	0.3992	0.002704367	chr11	70270716	70378580	ENSG00000131626
**PPFIA1**	ENST00000530548.5	16.8914	19.8501	1.8188	6.9254	-1.8820	0.2410	3.36834E-13	chr11	70364341	70384394	ENSG00000131626
**PPFIA1**	ENST00000648755.1	6.0036	1.7490	0.3951	0.5405	-1.3788	0.4456	0.027014209	chr11	70271921	70385312	ENSG00000131626
**PREX1**	ENST00000371941.4	15.4136	25.2872	8.6022	5.8581	-1.4431	0.2725	3.40387E-06	chr20	48624251	48827999	ENSG00000124126
**RNF11**	ENST00000242719.4	14.1451	17.0221	2.5387	4.6138	-1.5991	0.2682	8.86393E-08	chr1	51236272	51273447	ENSG00000123091
**RPL31**	ENST00000264258.8	5.8675	3.5201	9.1156	14.2838	1.4937	0.2799	2.77843E-06	chr2	101002288	101007267	ENSG00000071082
**SHANK2**	ENST00000445654.2	0.2770	0.0000	7.9686	3.3022	2.7112	0.4924	1.13254E-06	chr11	70566386	70659910	ENSG00000162105
**SHC3**	ENST00000375835.9	1.4989	1.9269	0.3429	0.2545	-1.5162	0.4828	0.023434999	chr9	89005770	89178818	ENSG00000148082
**SIAH2**	ENST00000312960.4	10.0554	16.3199	7.1327	4.7948	-1.4225	0.2945	3.45819E-05	chr3	150741124	150763169	ENSG00000181788
**SLFNL1**	ENST00000359345.5	6.5272	4.2334	1.3173	1.0948	-2.2690	0.4277	3.24679E-06	chr1	41015596	41023237	ENSG00000171790
**SNTB1**	ENST00000395601.7	6.7211	3.8320	0.7380	1.3287	-1.8251	0.4151	0.000239979	chr8	120535744	120813273	ENSG00000172164
**SNTB1**	ENST00000520717.1	5.2141	3.6992	0.3951	0.8019	-1.7748	0.4660	0.002492098	chr8	120811758	120812852	ENSG00000172164
**TAX1BP1**	ENST00000460059.1	8.4218	4.9792	30.1684	30.9073	2.1426	0.2636	2.76657E-14	chr7	27828699	27833151	ENSG00000106052
**TAX1BP1**	ENST00000488564.2	3.0027	5.0574	13.5947	11.0605	1.7325	0.3282	3.74043E-06	chr7	27827800	27844564	ENSG00000106052
**TCEA3**	ENST00000450454.7	2.2884	1.1474	0.0000	0.0000	-2.1150	0.4979	0.000446749	chr1	23380908	23424748	ENSG00000204219
**THBS1**	ENST00000260356.6	11.2244	19.7947	64.9251	54.5947	1.6999	0.2805	4.95257E-08	chr15	39581078	39599466	ENSG00000137801
**THBS1**	ENST00000560894.1	0.9752	0.9914	3.7878	7.0991	1.6228	0.4635	0.007432667	chr15	39590927	39591618	ENSG00000137801
**TLE1**	ENST00000376499.8	4.5978	6.7282	10.5936	10.7923	1.1334	0.3073	0.003872368	chr9	81583682	81689547	ENSG00000196781
**TNFRSF21**	ENST00000296861.2	0.7286	2.2612	8.0047	4.8752	1.9916	0.4707	0.000479015	chr6	47231531	47309905	ENSG00000146072
**TOB1**	ENST00000268957.3	12.0102	16.1181	30.2401	26.0160	1.1660	0.2030	3.03127E-07	chr17	50862979	50867978	ENSG00000141232
**TSHZ2**	ENST00000371497.10	6.9148	2.2388	20.1107	31.4548	2.0895	0.3092	6.20984E-10	chr20	52972357	53495330	ENSG00000182463
**TSPAN14**	ENST00000265450.5	4.4022	4.1440	0.3248	0.0000	-2.7837	0.4970	6.78783E-07	chr10	80515466	80519635	ENSG00000108219
**TTC39A**	ENST00000262676.9	22.3234	17.5786	37.4730	41.5785	1.0131	0.1627	1.8214E-08	chr1	51297217	51331346	ENSG00000085831
**USP3**	ENST00000561381.5	1.7647	2.5509	5.8591	5.4226	1.5219	0.4368	0.00787916	chr15	63587969	63591003	ENSG00000140455
**VMP1**	ENST00000592790.1	0.5541	0.0000	0.7902	2.3014	1.6201	0.5026	0.018211786	chr17	59837930	59842255	ENSG00000062716
**ZBED5**	ENST00000526020.5	2.0946	2.6067	9.9314	4.2090	1.3411	0.3839	0.007646643	chr11	10854618	10858796	ENSG00000236287

T47D ERα Y537S-Het vs. ERα WT

Gene name	Transcript ID	Avg WT Veh	Avg WT R5020	Avg Het Veh	Avg Het R5020	log2(FC)	StdErr	P-adj	Chr	Start	End	Gene stable ID
**ACSM3**	ENST00000614721.1	5.7280	2.9838	8.2014	13.4260	1.0771	0.2826	0.001834631	chr16	20674530	20682176	ENSG00000005187
**ADAMTS8**	ENST00000257359.7	0.0000	0.0000	1.1294	1.8637	1.9521	0.5457	0.003977175	chr11	130404922	130428609	ENSG00000134917
**ANKRD11**	ENST00000568100.2	0.2635	0.0000	0.8103	1.8416	1.5756	0.5563	0.032954379	chr16	89285165	89288977	ENSG00000167522
**ANKRD11**	ENST00000645212.1	1.1737	1.1678	6.1985	6.1529	1.3041	0.3718	0.004939228	chr16	89284263	89290656	ENSG00000167522
**ANKRD28**	ENST00000462657.1	3.7151	3.6070	7.5520	9.2631	1.1768	0.2892	0.000723375	chr3	15689839	15696231	ENSG00000206560
**B3GALNT1**	ENST00000651422.1	2.3742	1.0633	5.7621	4.9498	1.4485	0.4279	0.007224262	chr3	161085238	161092179	ENSG00000169255
**C14orf180**	ENST00000410013.1	2.7102	0.0000	14.5225	14.7937	2.5457	0.3475	1.89E-11	chr14	104581140	104589808	ENSG00000184601
**CBFA2T2**	ENST00000359606.3	0.5995	0.0000	1.5331	1.4755	1.4943	0.5561	0.04687095	chr20	33600335	33650036	ENSG00000078699
**CCDC40**	ENST00000374877.7	15.1775	11.7821	6.0524	10.3561	-1.0632	0.2575	0.00058277	chr17	80036631	80090819	ENSG00000141519
**CCDC40**	ENST00000572253.5	1.3898	1.5306	5.8161	6.8244	1.6936	0.3977	0.000356027	chr17	80081442	80097934	ENSG00000141519
**CDHR3**	ENST00000487084.1	0.9118	3.9469	1.7852	9.2445	1.4435	0.4060	0.004231757	chr7	105876795	105910598	ENSG00000128536
**CEBPA**	ENST00000498907.3	4.4360	5.0841	7.0218	7.1743	1.0037	0.3530	0.032070424	chr19	33299933	33302534	ENSG00000245848
**CRB2**	ENST00000460253.1	0.9371	1.7910	4.0774	3.7161	1.6589	0.4507	0.002841373	chr9	123368778	123380324	ENSG00000148204
**EHMT1**	ENST00000637318.1	1.6280	1.4533	0.0000	0.0000	-2.2019	0.5290	0.000516453	chr9	137742956	137747964	ENSG00000181090
**EHMT1**	ENST00000638071.1	6.6178	8.1193	0.8177	0.0000	-3.2924	0.4284	1.48E-12	chr9	137716926	137777946	ENSG00000181090
**ENAH**	ENST00000366844.7	0.2635	0.6755	3.8588	4.9719	1.8399	0.5243	0.004916006	chr1	225486834	225653142	ENSG00000154380
**ENPP6**	ENST00000296741.7	0.0000	0.0000	1.3424	1.0434	1.6146	0.5707	0.033216963	chr4	184088705	184217873	ENSG00000164303
**GRIK3**	ENST00000373091.8	6.6667	4.4643	13.5856	18.4068	1.3109	0.3729	0.004830356	chr1	36795526	37034515	ENSG00000163873
**GTF2E2**	ENST00000522833.5	3.7860	2.1015	11.2098	10.2953	1.6364	0.3492	6.07E-05	chr8	30584502	30612365	ENSG00000197265
**GUCY1A2**	ENST00000526355.7	0.8377	0.0000	15.4686	20.2980	3.6845	0.3913	9.84E-19	chr11	106674018	107018476	ENSG00000152402
**KRT37**	ENST00000225550.4	0.0000	0.4150	0.0000	3.5678	1.5382	0.5635	0.04243087	chr17	41420546	41424585	ENSG00000108417
**MCF2L2**	ENST00000461074.1	2.3726	2.8793	3.3007	9.5831	1.4804	0.3947	0.002269137	chr3	183372018	183400539	ENSG00000053524
**NANOS1**	ENST00000425699.3	0.3124	0.0000	1.5740	1.8141	1.5923	0.5107	0.015530965	chr10	119029713	119033730	ENSG00000188613
**NDUFB9**	ENST00000524241.2	239.4155	373.2338	128.3229	158.9637	-1.0267	0.0941	3.78E-25	chr8	124539161	124550162	ENSG00000147684

**NDUFB9**	ENST00000678753.1	259.4425	364.0375	117.2213	111.6440	-1.2910	0.1388	2.76E-18	chr8	124547002	124554406	ENSG00000147684
**NFIA**	ENST00000603233.2	7.1399	12.5314	0.8438	0.3386	-3.1057	0.3891	1.59E-13	chr1	61088148	61133028	ENSG00000162599
**PKDREJ**	ENST00000253255.7	0.3124	0.0000	1.5740	1.4425	1.8071	0.5500	0.009697843	chr22	46255662	46263343	ENSG00000130943
**PPFIA1**	ENST00000648755.1	2.9027	1.5055	6.1445	2.8850	1.5640	0.4025	0.001428076	chr11	70271921	70385312	ENSG00000131626
**PRAM1**	ENST00000423345.5	0.9118	0.0000	3.2076	2.5189	1.6294	0.5063	0.011766791	chr19	8490055	8502640	ENSG00000133246
**SIPA1L3**	ENST00000222345.11	3.5494	2.2833	9.2702	7.7361	1.0985	0.3658	0.021061072	chr19	37907207	38208369	ENSG00000105738
**SPATA17**	ENST00000470448.5	1.1248	1.4783	3.6373	7.3479	1.2962	0.4574	0.032840365	chr1	217631323	217774424	ENSG00000162814
**TAF8**	ENST00000372978.7	0.0000	1.6601	1.6131	3.4050	1.4695	0.5331	0.039802496	chr6	42050512	42057789	ENSG00000137413
**TBC1D7**	ENST00000421203.6	0.8866	0.0000	3.9713	0.7377	1.6548	0.5320	0.015802918	chr6	13274848	13328544	ENSG00000145979
**UBE3A**	ENST00000631247.1	1.1737	1.7137	7.2720	7.1522	1.7444	0.3618	3.29E-05	chr15	25354406	25357575	ENSG00000114062
**WNT7B**	ENST00000339464.9	0.0000	0.0000	4.0849	5.4810	2.4225	0.4498	2.24E-06	chr22	45920365	45977162	ENSG00000188064

T47D ERα Y537S-Hom vs. ERα WT

Gene name	Transcript ID	Avg WT Veh	Avg WT R5020	Avg Hom Veh	Avg Hom R5020	log2(FC)	StdErr	P-adj	Chr	Start	End	Gene stable ID
**ADAMTS8**	ENST00000257359.7	0.0000	0.0000	1.8746	2.1936	1.4455	0.5517	0.039502644	chr11	130404922	130428609	ENSG00000134917
**ANGPT1**	ENST00000521950.1	18.2031	10.2001	3.3361	2.7624	-2.1494	0.3032	2.93E-11	chr8	107283996	107321993	ENSG00000154188
**BCAR3**	ENST00000479503.2	1.4151	0.8301	3.1426	3.8999	1.4455	0.4922	0.017232335	chr1	93592305	93648822	ENSG00000137936
**BCAR3**	ENST00000490377.1	0.2871	1.0633	2.0643	3.1719	1.3600	0.5056	0.033133302	chr1	93589082	93590378	ENSG00000137936
**C17orf67**	ENST00000397861.7	0.0000	0.0000	2.3500	1.6249	2.0381	0.5682	0.002286793	chr17	56791912	56833920	ENSG00000214226
**CCDC40**	ENST00000374876.4	2.2275	4.4894	9.2211	8.3971	1.4608	0.2883	4.68E-06	chr17	80036657	80066770	ENSG00000141519
**CCDC40**	ENST00000374877.7	15.1775	11.7821	4.6340	4.5276	-1.5885	0.2734	9.17E-08	chr17	80036631	80090819	ENSG00000141519
**CD44**	ENST00000442151.6	0.2635	0.0000	4.4443	1.3095	1.7296	0.5596	0.011120042	chr11	35139305	35223320	ENSG00000026508
**CEBPA**	ENST00000498907.3	4.4360	5.0841	1.5628	0.0000	-1.7685	0.4436	0.000525835	chr19	33299933	33302534	ENSG00000245848
**CHDH**	ENST00000315251.11	160.2692	173.4154	31.7675	29.7705	-2.5437	0.1288	3.74E-84	chr3	53812334	53846419	ENSG00000016391
**CLDN9**	ENST00000445369.3	2.8049	4.3326	16.7197	20.7062	2.1297	0.2848	1.79E-12	chr16	3012922	3014505	ENSG00000213937
**CMTM6**	ENST00000205636.4	1.5113	10.6903	14.7813	29.6964	1.4510	0.3021	1.65E-05	chr3	32481311	32502852	ENSG00000091317
**CNOT4**	ENST00000428680.6	1.7984	1.8661	1.8746	4.7062	1.2061	0.4473	0.032564991	chr7	135386150	135510076	ENSG00000080802
**COL27A1**	ENST00000485397.1	12.7844	9.9375	4.9496	5.4597	-1.0775	0.2759	0.000717599	chr9	114240219	114246121	ENSG00000196739
**CPVL**	ENST00000265394.10	3.0241	1.9184	0.6236	0.6313	-1.3879	0.5057	0.028984931	chr7	28995234	29146537	ENSG00000106066
**EHMT1**	ENST00000495657.5	0.6247	0.8301	2.1864	4.2596	2.1118	0.5377	0.000658705	chr9	137738606	137754221	ENSG00000181090
**FBXO22**	ENST00000308275.8	15.5608	16.2974	5.5018	6.5188	-1.1987	0.2382	5.55E-06	chr15	75903877	75942511	ENSG00000167196
**FNDC3B**	ENST00000478016.1	22.4231	28.9268	53.0452	52.0013	1.3100	0.1781	4.42E-12	chr3	172126972	172227263	ENSG00000075420
**GTF2E2**	ENST00000522833.5	3.7860	2.1015	0.0000	2.6190	-1.4743	0.4304	0.003910737	chr8	30584502	30612365	ENSG00000197265
**HHIP**	ENST00000296575.8	5.1995	3.9197	0.0000	0.0000	-2.7373	0.4690	7.91E-08	chr4	144646155	144745271	ENSG00000164161
**JPH2**	ENST00000372980.4	0.5269	0.3628	26.6382	23.4807	4.8240	0.4162	3.33E-29	chr20	44106589	44187188	ENSG00000149596
**MTA1**	ENST00000435036.6	0.6247	1.8160	10.1304	10.3343	2.8188	0.4015	4.67E-11	chr14	105419819	105470727	ENSG00000182979
**MTA1**	ENST00000469140.2	0.2871	2.0752	4.3015	5.0502	1.5427	0.4168	0.001523764	chr14	105463552	105467192	ENSG00000182979
**NANOS1**	ENST00000425699.3	0.3124	0.0000	4.0311	2.6499	2.0752	0.5044	0.000320685	chr10	119029713	119033730	ENSG00000188613
**PAN3**	ENST00000483842.1	2.6392	3.8640	17.6720	17.6758	1.4395	0.2886	6.84E-06	chr13	28239556	28256539	ENSG00000152520
**PBX1**	ENST00000465089.2	12.8269	17.3814	5.7161	5.9033	-1.2310	0.2597	2.20E-05	chr1	164812727	164850357	ENSG00000185630
**PEX14**	ENST00000472851.1	31.2572	35.6187	94.0661	108.4857	1.5556	0.1103	4.31E-43	chr1	10472287	10536572	ENSG00000142655
**PPFIA1**	ENST00000525530.1	6.0624	4.1767	2.2371	0.6155	-1.4045	0.3756	0.001329875	chr11	70329892	70332046	ENSG00000131626
**PXDC1**	ENST00000380283.5	2.0398	2.4915	1.1796	0.3312	-1.1351	0.4380	0.042364824	chr6	3722618	3751713	ENSG00000168994
**RARA**	ENST00000394081.7	24.9832	20.9862	10.5305	7.1964	-1.1104	0.1976	2.63E-07	chr17	40342362	40356795	ENSG00000131759
**RARA**	ENST00000394086.7	183.8127	168.0701	79.3431	65.6777	-1.1395	0.1176	1.55E-20	chr17	40341387	40356795	ENSG00000131759
**RPL31**	ENST00000409028.8	3.3049	5.9418	9.0522	12.2903	1.0957	0.3007	0.00187148	chr2	101002282	101019728	ENSG00000071082
**SSBP3**	ENST00000448572.1	11.7147	15.5675	1.5590	1.5313	-1.9572	0.3668	1.20E-06	chr1	54412707	54413466	ENSG00000157216
**ST3GAL3**	ENST00000479383.6	1.3898	2.6211	5.6032	5.1316	1.2182	0.4467	0.030291045	chr1	43707557	43838261	ENSG00000126091
**UMPS**	ENST00000232607.7	107.7374	105.8296	30.2730	41.9805	-1.9808	0.1550	1.97E-35	chr3	124730451	124749273	ENSG00000114491
**UMPS**	ENST00000479719.5	5.5593	4.5123	0.8171	1.5155	-1.5053	0.3915	0.000894575	chr3	124730451	124744757	ENSG00000114491
**WNT7B**	ENST00000339464.9	0.0000	0.0000	12.8598	13.2680	3.9892	0.4362	2.25E-18	chr22	45920365	45977162	ENSG00000188064
**XRCC4**	ENST00000509268.1	0.3124	0.0000	3.6518	7.0992	3.0465	0.5452	3.13E-07	chr5	83104907	83259339	ENSG00000152422
**ZNF703**	ENST00000331569.6	0.0000	0.6755	1.2004	1.5939	1.3924	0.5455	0.046523544	chr8	37695781	37700019	ENSG00000183779

T47D ERα Y537S-Hom vs. ERα Y537S-Het

Gene name	Transcript ID	Avg Het Veh	Avg Het R5020	Avg Hom Veh	Avg Hom R5020	log2(FC)	StdErr	P-adj	Chr	Start	End	Gene stable ID

**ANKH**	ENST00000284268.8	58.3598	64.4799	35.6481	21.1540	-1.1273	0.1777	2.70E-09	chr5	14704799	14871778	ENSG00000154122
**ANKRD28**	ENST00000462657.1	7.5520	9.2631	3.0543	0.2844	-1.7393	0.3102	2.08E-07	chr3	15689839	15696231	ENSG00000206560
**ANKRD28**	ENST00000463533.1	72.8475	89.4134	33.5484	39.4187	-1.0862	0.1117	7.64E-21	chr3	15679300	15686453	ENSG00000206560
**C14orf180**	ENST00000410013.1	14.5225	14.7937	4.6133	4.2469	-1.8016	0.3166	1.33E-07	chr14	104581140	104589808	ENSG00000184601
**C17orf67**	ENST00000397861.7	0.0000	0.0000	2.3500	1.6249	2.1019	0.5682	0.001227236	chr17	56791912	56833920	ENSG00000214226
**CBWD2**	ENST00000259199.9	6.6952	4.1757	8.2650	13.0796	1.3286	0.3115	0.000138245	chr2	113437690	113496204	ENSG00000136682
**CBWD2**	ENST00000468417.1	91.0547	75.8907	256.9170	202.4770	1.3972	0.1092	1.27E-35	chr2	113481517	113496121	ENSG00000136682
**CBX4**	ENST00000269397.9	2.1043	0.7982	0.0000	0.2844	-1.5237	0.5696	0.027956679	chr17	79833155	79839440	ENSG00000141582
**CD44**	ENST00000442151.6	0.0000	0.3991	4.4443	1.3095	1.9253	0.5598	0.002991026	chr11	35139305	35223320	ENSG00000026508
**CDKL3**	ENST00000265334.9	6.4208	6.5574	19.3062	18.9379	1.3939	0.2408	7.55E-08	chr5	134298423	134367171	ENSG00000006837
**CENPF**	ENST00000366955.8	28.0376	56.2476	6.0279	11.6158	-1.9441	0.2213	3.91E-17	chr1	214603194	214664571	ENSG00000117724
**CHDH**	ENST00000315251.11	202.0626	228.1092	31.7675	29.7705	-2.9349	0.1276	2.43E-114	chr3	53812334	53846419	ENSG00000016391
**CLDN9**	ENST00000445369.3	2.3098	4.4155	16.7197	20.7062	2.5153	0.2954	3.86E-16	chr16	3012922	3014505	ENSG00000213937
**CMTM6**	ENST00000205636.4	1.6615	18.6889	14.7813	29.6964	1.2369	0.2965	0.000202259	chr3	32481311	32502852	ENSG00000091317
**CMYA5**	ENST00000446378.3	5.9259	9.1205	0.3118	1.2779	-1.6245	0.3985	0.000294924	chr5	79689835	79800222	ENSG00000164309
**COG7**	ENST00000307149.10	0.5582	0.7982	3.6479	2.2720	1.4240	0.4748	0.011628086	chr16	23388492	23453189	ENSG00000168434
**CPB2**	ENST00000181383.10	13.2165	10.4363	6.3189	7.1004	-1.0396	0.2461	0.000163744	chr13	46053185	46105033	ENSG00000080618
**CPVL**	ENST00000265394.10	3.1871	1.4700	0.6236	0.6313	-1.3137	0.5048	0.03362076	chr7	28995234	29146537	ENSG00000106066
**CRB2**	ENST00000460253.1	4.0774	3.7161	1.1796	0.9783	-1.8112	0.4532	0.000404656	chr9	123368778	123380324	ENSG00000148204
**CSNK1G2**	ENST00000255641.13	1.9061	1.1368	3.1725	5.7593	1.3072	0.4077	0.006296519	chr19	1941171	1981338	ENSG00000133275
**EDN2**	ENST00000372587.5	0.5451	0.3662	3.3622	1.4219	1.4419	0.5316	0.025326856	chr1	41478774	41484683	ENSG00000127129
**ENAH**	ENST00000366844.7	3.8588	4.9719	0.3118	1.7063	-1.4177	0.5142	0.022554937	chr1	225486834	225653142	ENSG00000154380
**ENPP6**	ENST00000296741.7	1.3424	1.0434	0.0000	0.0000	-1.6557	0.5707	0.015238981	chr4	184088705	184217873	ENSG00000164303
**FBXO22**	ENST00000308275.8	18.7757	23.4740	5.5018	6.5188	-1.4434	0.2338	7.77E-09	chr15	75903877	75942511	ENSG00000167196
**HHIP**	ENST00000296575.8	12.9205	9.8799	0.0000	0.0000	-3.6334	0.4598	5.39E-14	chr4	144646155	144745271	ENSG00000164161
**HSPA12B**	ENST00000254963.7	0.0000	0.7048	3.9597	2.6032	1.9428	0.5091	0.00080548	chr20	3732684	3753111	ENSG00000132622
**IRX4**	ENST00000231357.7	0.5712	1.0764	2.9529	1.2627	1.4490	0.4833	0.011668172	chr5	1877412	1882925	ENSG00000113430
**JPH2**	ENST00000372980.4	0.8307	0.3386	26.6382	23.4807	5.0287	0.4216	4.68E-31	chr20	44106589	44187188	ENSG00000149596
**KCNH8**	ENST00000328405.7	0.7898	1.3546	0.0000	0.0000	-1.5243	0.5691	0.027724845	chr3	19148509	19535642	ENSG00000183960
**LIPM**	ENST00000404743.9	0.5377	3.3171	0.4339	0.3470	-1.6704	0.5258	0.006880733	chr10	88802729	88820546	ENSG00000173239
**MTA1**	ENST00000435036.6	2.4587	1.4755	10.1304	10.3343	2.5173	0.3791	4.12E-10	chr14	105419819	105470727	ENSG00000182979
**PBX1**	ENST00000465089.2	32.4966	22.5538	5.7161	5.9033	-1.9930	0.2500	3.09E-14	chr1	164812727	164850357	ENSG00000185630
**PKDREJ**	ENST00000253255.7	1.5740	1.4425	0.3832	0.0000	-1.3623	0.5465	0.043620439	chr22	46255662	46263343	ENSG00000130943
**PRAM1**	ENST00000423345.5	3.2076	2.5189	0.3832	0.6313	-1.4064	0.4960	0.018249835	chr19	8490055	8502640	ENSG00000133246
**RARA**	ENST00000394081.7	33.0649	24.9956	10.5305	7.1964	-1.4419	0.1923	1.12E-12	chr17	40342362	40356795	ENSG00000131759
**RARA**	ENST00000394086.7	233.6319	229.0950	79.3431	65.6777	-1.5670	0.1162	1.66E-39	chr17	40341387	40356795	ENSG00000131759
**SIPA1L3**	ENST00000222345.11	9.2702	7.7361	2.7386	5.6530	-1.0332	0.3624	0.0175038	chr19	37907207	38208369	ENSG00000105738
**SSBP3**	ENST00000448572.1	7.4627	3.5019	1.5590	1.5313	-1.2151	0.3748	0.005638408	chr1	54412707	54413466	ENSG00000157216
**TAF8**	ENST00000372978.7	1.6131	3.4050	0.6950	0.9935	-1.3763	0.5298	0.033991429	chr6	42050512	42057789	ENSG00000137413
**TBC1D7**	ENST00000421203.6	3.9713	0.7377	0.0000	1.2311	-1.7276	0.5318	0.00553582	chr6	13274848	13328544	ENSG00000145979
**UMPS**	ENST00000232607.7	64.1845	67.0131	30.2730	41.9805	-1.1111	0.1575	2.57E-11	chr3	124730451	124749273	ENSG00000114491
**WNT7B**	ENST00000339464.9	4.0849	5.4810	12.8598	13.2680	1.5667	0.3172	6.74E-06	chr22	45920365	45977162	ENSG00000188064

Supplementary Table 4 Genes differentially expressed among patient datasets with ERα Y537S, relative to ERα WT.

Patient Tumor RNA-seq Differentially Expressed Genes

Gene Name	Log2(FC)	SEM	Gene Name	Log2(FC)	SEM	Gene Name	Log2(FC)	SEM
TFF1	7.987	1.28	UBA52	1.45	0.2371	BBIP1	0.711	0.1741
ZNF716	7.46	0.3621	LZTFL1	1.446	0.1893	ZNF230	0.7091	0.1427
MMP26	7.3263	0.3401	CCDC189	1.442	0.2639	CCP110	0.709	0.1732
PIGY	7.303	0.8773	PLS1	1.442	0.2435	HNRNPK	0.708	0.1547
TSPAN19	6.9817	1.183	TP53I11	1.442	0.2122	PDE12	0.708	0.1589
CFAP126	6.966	0.8708	ZNF493	1.441	0.2857	MIER1	0.706	0.1401
IL20	6.959	0.9588	TRIM6-TRIM34	1.4408	0.3008	POM121C	0.706	0.1457
GPR139	6.87	1.231	BTBD8	1.4369	0.2583	USP7	0.705	0.1174
LINC01980	6.641	0.5398	ZNF418	1.4361	0.2661	HARBI1	0.703	0.1492
CST9	6.636	1.292	KCNK6	1.435	0.2574	RBM43	0.703	0.1681
NPY1R	6.5023	1.03	BCAR3-AS1	1.4335	0.3601	RNF25	0.703	0.1561
SERF1A	6.415	0.4126	EIF5A2P1	1.432	0.2309	PHF8	0.7	0.1792
RSC1A1	6.268	0.7893	SAMD7	1.432	0.3509	RPL4	0.7	0.1702
ZNF729	6.0572	0.4025	MBOAT1	1.429	0.2273	MTCO2P21	0.699	0.1374
PGR	6.018	0.9404	GAMT	1.427	0.3175	SMIM12	0.695	0.1502
MYL1	6.017	1.278	LGALS9	1.427	0.3593	XPO6	0.694	0.1495
LINC01016	5.905	0.7281	ZNF385A	1.427	0.3099	LDB1	0.693	0.1483
MRLN	5.901	0.5489	OR7E12P	1.426	0.2505	CEP97	0.69	0.1109
LGALS7	5.8738	0.6007	MAGIX	1.425	0.3139	WDR24	0.69	0.1622
LRRC24	5.85	0.8273	BLVRA	1.424	0.2585	RARA-AS1	0.6889	0.1764
WFDC6	5.825	0.4629	RAD51D	1.424	0.1635	RPL30P4	0.688	0.1105
APELA	5.695	0.5662	ZNF442	1.4239	0.2245	FAM53B	0.687	0.1522
FAM25A	5.6568	1.355	SEMA3F	1.422	0.3384	SPRING1P2	0.686	0.165
PRLH	5.656562	0.807	FAM120AOS	1.421	0.204	STX3	0.686	0.1569
AGR3	5.643	0.8148	N4BP2L2	1.42	0.32	REPIN1	0.685	0.1501
GFRA1	5.6283	0.6735	RPL27A	1.418	0.3183	TM2D3	0.684	0.1228
XAGE3	5.536	0.6245	ZNF655	1.415	0.1997	RSBN1L	0.681	0.1141
CGB5	5.518	0.9655	PTK2	1.414	0.2847	ETFBKMT	0.6786	0.1706
AMIGO3	5.5062	0.6911	RASEF	1.41	0.2813	POLR2M	0.677	0.1531
DYDC1	5.3088	0.4636	TMC4	1.41	0.2309	ZNF136	0.677	0.1699
KRTAP4-1	5.193	0.759	LCMT2	1.404	0.1719	ZNF79	0.677	0.1595
PAGE2	5.095	0.4152	PGGT1BP2	1.402	0.08674	DNAJB6P1	0.6766	0.1702
GRM7	5.072	0.4005	XRRA1	1.402	0.3146	ZNF567	0.676	0.155
PLPPR3	5.0316	0.4183	SLC9A3R1	1.4	0.3288	RN7SL737P	0.67326	0.1795
CGB8	5.019	0.3924	CCND1	1.399	0.3095	PTPN9	0.673	0.1532
TCL1B	4.9921	1.123	NME6	1.399	0.1548	RUFY2	0.673	0.1583
C10orf82	4.985	0.5681	C4orf36	1.397	0.1956	RIC8A	0.672	0.1126
OR10H1	4.812	0.6131	RAD17P1	1.3962	0.333	SETD4	0.672	0.1308
MAGEA9B	4.805	1.048	INO80E	1.395	0.1916	SRP72	0.672	0.137
TFF3	4.8	0.7006	NUTM2D	1.3943	0.2674	HELQ	0.667	0.1531
PRAMEF10	4.789	0.3611	DGAT1	1.394	0.2298	IRAK4	0.666	0.1641
PAGE2B	4.758	1.02	SLC22A5	1.394	0.1929	UBR1	0.665	0.166
RPL41	4.756	0.6321	LRRC69	1.39345	0.3089	ZNF213	0.665	0.1652
SCUBE2	4.703	0.7963	SUGT1	1.379	0.2511	CT70	0.663	0.1217
MEIKIN	4.661	0.6659	UBQLN3	1.379	0.2961	DUXAP1	0.663	0.1496
ZACN	4.612	0.5829	EME2	1.378	0.2276	HSP90B3P	0.663	0.1749
SERPINA5	4.609	0.6195	RAB37	1.3742	0.3311	LRIG2	0.662	0.1547
GSTT2	4.596	0.7144	RN7SL657P	1.3728	0.2747	BSDC1	0.661	0.1555
ELOVL2-AS1	4.57	0.2949	ZNF525	1.37	0.2716	CTDSP2	0.66	0.1522
MPPED2-AS1	4.561	0.4627	GNB1L	1.3694	0.2414	PTER	0.659	0.1641
ZNF286A	4.509	0.6813	TFDP1P1	1.368	0.1585	ATF7	0.658	0.1256
GSTA3	4.50161	0.5498	PSMG4	1.367	0.2726	TMEM231P1	0.658	0.1722
KLF2P2	4.489	0.2494	ZNF772	1.3661	0.2692	PDIA3	0.657	0.1438
GREB1	4.4386	0.7683	RD3	1.366	0.3798	SNX2	0.656	0.1711
COX7B2	4.435	0.586	XBP1	1.366	0.2973	PPT2	0.655	0.1536
GRIK3	4.432	0.7398	H2AC15	1.3654	0.3567	TRAPPC11	0.655	0.09732
MAPT	4.41	0.4663	GGCTP1	1.364	0.214	ENPP7P14	0.654	0.1643
ZNF679	4.383	0.6562	RN7SL316P	1.363	0.03245	PPIAP32	0.653	0.1523
AGR2	4.369	0.6905	SMIM15-AS1	1.363	0.2865	ZNF148	0.653	0.1228
NKAIN1	4.3569	0.9649	MRTFB	1.362	0.1932	ZNF569	0.6522	0.1205
VCX	4.356	0.7989	OR1L6	1.358	0.3034	ZNF235	0.65133	0.1355
TPRG1	4.3087	0.7684	ZNF415	1.352	0.2101	PHF10P1	0.651	0.1542
TMEM26	4.285	0.4633	STK36	1.35	0.2548	FBXL6	0.649	0.1701
OTOS	4.257	0.6692	CCDC85C	1.348	0.2505	ZNF443	0.649	0.1556
MAGEB17	4.2381	0.6138	RBBP4	1.347	0.1462	LCOR	0.647	0.1062
FRMPD2	4.2072	0.6846	IQCD	1.3469	0.311	ZMYM6	0.647	0.1435
ASIC2	4.1933	0.9055	ZNF487	1.3456	0.2147	PJA2	0.643	0.1167
LHX1	4.1894	0.9423	TSPAN15	1.344	0.2662	FAM133FP	0.642	0.1629

PRR35	4.184	0.744	DALRD3	1.34	0.1767	CYFIP1	0.638	0.1527
DOK7	4.16	0.3778	TXNP4	1.34	0.1679	RPS4XP20	0.637	0.1437
PRTN3	4.1555	0.6755	CNOT7	1.339	0.2727	SCAMP4	0.636	0.1242
LINC02568	4.1492	0.567	ISYNA1	1.338	0.2737	HELZ	0.635	0.1525
AQP12A	4.14	0.3887	WDR90	1.338	0.2989	THAP9	0.6342	0.1498
UGT1A8	4.137	0.6685	USP6NL-AS1	1.337	0.173	FANCF	0.634	0.1374
OR6B3	4.125	0.5833	EIF3C	1.333	0.2359	TMEM161B	0.633	0.1486
POTEI	4.124	0.747	SCDP1	1.33	0.274	CNN2P6	0.632	0.119
PGA5	4.1	0.3883	CTC1	1.329	0.2741	IFRD2	0.631	0.1552
ZNF99	4.096	0.4183	PPP2R2DP1	1.328	0.2483	ADH5	0.63	0.1478
PABPC1L2A	4.085	0.4368	TJP3	1.327	0.2754	DPP3	0.628	0.1487
AWAT1	4.065	0.3571	LILRA2	1.3265	0.2983	TMEM9B	0.626	0.1444
HSFX2	4.043	0.6747	MAPK1IP1L	1.324	0.259	DHX32	0.625	0.1645
CHAD	3.984	0.6698	SLC43A2	1.324	0.2393	NANOGP3	0.625	0.06051
DSCR8	3.948	0.5901	LRRC14	1.322	0.2026	ZNF398	0.625	0.08804
BECN2	3.935	0.4606	PDPK2P	1.321	0.3222	CHST14	0.622	0.1184
MUC5B	3.922	0.9378	BMPR1AP1	1.32	0.2936	HMGN1P24	0.619	0.1453
CA12	3.869	0.5311	PBX1	1.32	0.2066	MFSD8	0.619	0.109
NEK10	3.862	0.4427	RSL1D1	1.317	0.1883	PYM1	0.619	0.1212
CT62	3.8585	0.5693	PHLDB3	1.316	0.2173	OTUD7B	0.618	0.1496
STMND1	3.849	0.5839	ARMH2	1.315	0.1861	RPS14P4	0.613	0.1396
HBG1	3.84	0.5247	CRTAP	1.315	0.2083	H3P2	0.612	0.1696
APOA2	3.822	0.9237	ZNF320	1.315	0.1845	NOLC1	0.611	0.1156
SPPL2C	3.814	0.4836	CERS6	1.314	0.2468	POM121	0.611	0.1435
C1orf167	3.7506	0.5538	CHMP4A	1.313	0.2253	RNF34	0.611	0.0989
TBC1D3L	3.74132	0.7181	MREG	1.313	0.2142	STX18	0.611	0.1267
MAGEB6	3.734	0.5119	HNRNPA1P53	1.311	0.1605	RETREG3	0.609	0.1306
IGFL3	3.7329	0.632	SNRNP35	1.311	0.169	TOMM20L-DT	0.608	0.1554
LGALS9B	3.731	0.6565	PSENEN	1.31	0.3143	USP51	0.60719	0.1539
CLSTN2	3.723483	0.6654	EMC10	1.305	0.2052	ATP5MC2P4	0.606	0.1328
MAG	3.7147	0.5241	LRRC37A2	1.303	0.3203	HNRNPH2	0.606	0.1087
RIMBP3B	3.703	0.7137	NSA2	1.3	0.2371	OR52P1P	0.606	0.1565
ZNF705A	3.701	0.6181	FAR1	1.297	0.1416	GPATCH4	0.605	0.1579
DEFA3	3.689	0.918	CLDN25	1.296	0.2655	DNAAF5	0.604	0.1132
CLCA4-AS1	3.686	0.176	OR52H1	1.294	0.2964	LINC02685	0.604	0.1595
SPZ1	3.685	0.5609	GLIPR1L2	1.29394	0.3033	ATE1	0.602	0.1273
NBPF6	3.678	0.785	RN7SL15P	1.2931	0.2347	LINC00691	0.601	0.0954
OR2T33	3.663	0.4039	H2BC12	1.293	0.1631	ZNF570	0.5982	0.1413
HERC2P7	3.6535	0.5049	NBPF22P	1.293	0.1308	ELP1	0.597	0.1363
SPRR2F	3.646	0.4843	RPL15	1.292	0.2367	NDUFV3	0.597	0.1306
SULT1A3	3.645	0.5495	ZNF239	1.292	0.2685	PDXDC1	0.595	0.1519
GOLGA8S	3.644	0.2591	HEXA	1.29	0.2173	FNTB	0.593	0.1251
THBS1-AS1	3.6348	0.6778	LRRC8E	1.29	0.2164	ZNF81	0.5906	0.09518
DEGS2	3.63	0.6128	ADAD1P2	1.288	0.08492	C19orf47	0.583	0.1306
MYT1L	3.626	0.6101	ZNF701	1.288	0.1741	METTL2B	0.583	0.1388
PSG3	3.621	0.8322	JADE2	1.287	0.2687	RNF207-AS1	0.582	0.1404
CLPSL2	3.5962	0.8674	MAPKAPK2	1.286	0.2214	SPTLC2	0.582	0.1572
UGT1A9	3.587	0.6208	ZNF793	1.2813	0.3133	AFF4	0.58	0.1253
GTF2H2B	3.5845	0.5932	RPL12P13	1.28	0.3028	EDC3	0.576	0.1188
AZU1	3.5827	0.5967	ZNF322	1.279	0.2186	ZNF584	0.575	0.1276
CADM2	3.5814	0.3984	GAST	1.276	0.3658	TOPBP1	0.569	0.1344
UBD	3.563	0.9172	NUDT16L1	1.276	0.2473	NUDT14	0.568	0.1567
VCX3B	3.553	0.5489	TXNP6	1.274	0.2021	ZNF564	0.568	0.1079
WFDC10A	3.542	0.8373	ZSCAN12P1	1.2739	0.2719	ZNF668	0.564	0.1226
ARMC3	3.54	0.5158	ZNF652	1.272	0.258	PIGO	0.561	0.1
PCDHA4	3.5387	0.5231	DNASE1	1.268	0.2702	PPP2R2D	0.56	0.148
SSTR5-AS1	3.533	0.546	THTPA	1.268	0.1948	CSTF3	0.558	0.117
MARCHF10	3.5162	0.5235	GPR27	1.266	0.3108	GGNBP2	0.554	0.1532
NXNL2	3.508	0.5696	ZNF169	1.2657	0.2562	ZNF768	0.554	0.1478
ADAMTS15	3.495	0.5805	TMEM192	1.265	0.2137	TAOK1	0.553	0.0948
SMIM22	3.492	0.5939	NARF-AS2	1.2606	0.2781	ZNF17	0.55	0.1238
SPRR2A	3.487	0.4824	FAM241A	1.259	0.2558	CGGBP1	0.548	0.1082
SERF1B	3.476	0.5546	MTND5P8	1.259	0.07259	PCGF3	0.547	0.1303
OR13H1	3.474	0.7529	GMPPB	1.257	0.2306	LINC02816	0.546	0.1531
TMEM191B	3.466	0.6586	CUX1	1.256	0.2273	OTUD4	0.543	0.1174
SMR3B	3.464	0.5606	CPSF2	1.254	0.1539	ZNF420	0.543	0.142
SLX1B	3.461	0.5148	RNF41	1.254	0.1919	ZNF607	0.543	0.1335
B3GNT6	3.459	0.7032	EMP2	1.253	0.2569	ZSCAN2	0.543	0.1361
UGT2A2	3.454	0.7776	TM7SF2	1.253	0.2981	LDHAL6EP	0.541	0.1227
HTR1E	3.435	0.3625	RBM12	1.252	0.1391	FAM53A	0.5384	0.1424
PPP1R42	3.4237	0.4624	JMJD8	1.247	0.2419	AP1G1	0.532	0.1346
DNALI1	3.419	0.3574	PRPF40A	1.247	0.1904	TYW5	0.532	0.1244
CCL4L2	3.396	0.6457	KRT18	1.246	0.3039	ING2	0.531	0.1364
ANKRD63	3.385	0.4009	WDR25	1.245	0.1622	CLPX	0.527	0.1275
NAT1	3.378	0.7518	CERCAM	1.244	0.2393	KIFBP	0.526	0.1251
FANCD2OS	3.371	0.3755	NUDT6	1.244	0.2733	TADA2B	0.525	0.1408

GP2	3.358	0.8731	OTOP2	1.243	0.2565	DENND6A	0.521	0.1069
ESR1	3.345	0.5678	INTU	1.2427	0.1894	SPNS1	0.519	0.1129
SLC35D3	3.345	0.3395	RPL21P134	1.242	0.1786	EIF2AK3	0.508	0.1274
PRSS57	3.341	0.7712	SHROOM3	1.242	0.2177	CDKN2AIP	0.507	0.1083
TBC1D28	3.339	0.5739	CMBL	1.24	0.3136	SEMA6A-AS2	0.495	0.1349
SULT1C2	3.3283	0.6333	ZNF606	1.24	0.205	EEF1E1P1	0.494	0.1155
COX6C	3.322	0.6212	RAB17	1.239	0.2726	XRCC6P1	0.49	0.1398
SYTL5	3.322	0.7036	KRT18P11	1.238	0.2323	EWSR1	0.489	0.1088
GPR21	3.307	0.5464	FOXD4L5	1.237	0.299	RBM15	0.488	0.1185
NDUFC2-KCTD14	3.291	0.5553	STXBP4	1.2348	0.1763	SRSF2	0.487	0.1295
RGS21	3.274	0.4135	DDX10	1.234	0.2144	NAP1L4	0.484	0.08059
LINC02747	3.271	0.6181	IFI27L1	1.234	0.2914	NDUFA3P1	0.484	0.1318
LHFPL5	3.2505	0.6206	AP1S2	1.233	0.2843	SETD3	0.484	0.1135
ASMT	3.245	0.6788	RAD21	1.233	0.2744	SLC9A8	0.473	0.1209
KRT8P18	3.239	0.5175	ERAP1	1.226	0.2059	DNAJB12P1	0.466	0.1124
FRG2B	3.238	0.5272	EIF1AXP2	1.225	0.1825	ZNF180	0.462	0.1143
EML5	3.2323	0.4573	KLHDC2	1.225	0.1902	PRPF6	0.457	0.1187
AMZ1	3.227	0.43	ZNF808	1.225	0.2342	KRT18P19	0.455	0.09125
C16orf92	3.215	0.3807	ZNF568	1.2248	0.1784	RRM1	0.454	0.1125
KRTAP17-1	3.206	0.446	SBK1	1.224	0.2673	TRIM32	0.452	0.1102
CCDC182	3.201	0.5431	SLC9A3R2	1.222	0.2611	ANKRD26P4	0.449	0.1256
SPDEF	3.187	0.5918	PRRT1	1.22	0.2862	SF3A3	0.447	0.11
CTAGE15	3.179	0.5786	HSP90AA4P	1.216	0.09321	TRAF6	0.447	0.08543
C14orf178	3.178	0.4808	THOP1	1.216	0.2504	MFAP1	0.427	0.0983
CFAP99	3.178	0.5322	ORAI2	1.214	0.2186	LYSMD3	0.423	0.111
SETSIP	3.157	0.5311	MTND5P12	1.209	0.2893	LINC00677	0.422	0.1165
RPS29	3.141	0.5293	TBL3	1.209	0.1503	SLC35A4	0.412	0.11
CITED1	3.133	0.6311	LBHD1	1.2082	0.2131	TBC1D10B	0.412	0.1045
OR2T8	3.133	0.5836	PGGT1B	1.208	0.1751	CCDC43	0.408	0.1009
SMIM21	3.12	0.2062	TAS2R46	1.208	0.2745	PAK2	0.406	0.1019
CTRB2	3.119	0.3962	MYOF	1.207	0.2346	ZNF2	0.405	0.07726
DPRX	3.116	0.4047	ZNF226	1.207	0.1846	Metazoa_SRP	0.4013	0.1039
BACH1-AS1	3.1066	0.6677	ULK4	1.2019	0.2274	PARG	0.397	0.1047
SMG1P4	3.101	0.2068	BPTF	1.201	0.2528	SACM1L	0.386	0.1027
LY6G6E	3.098	0.5441	CSNK1A1L	1.201	0.2149	ZBTB14	0.386	0.08778
EVL	3.07	0.4782	STRBP	1.201	0.1522	ZKSCAN5	0.321	0.06393
TMEM211	3.067	0.3753	BCL9L	1.199	0.2712	CBX3P6	-0.243	0.04325
RAG2	3.062	0.3298	ROGDI	1.198	0.2276	SUPT20HL1	-0.276	0.06827
MAGEB3	3.056	0.3037	CISH	1.196	0.2941	WBP11P1	-0.286	0.06148
UMOD	3.046	0.5047	ABCG1	1.195	0.2399	CICP26	-0.333	0.06506
STARD10	3.038	0.3377	DUSP28	1.194	0.2156	KLF18	-0.333	0.07274
FOXA1	3.02	0.4854	ARL3	1.193	0.2395	LINC00477	-0.333	0.07274
SGCZ	3.016	0.541	WDR73	1.193	0.1409	LINC02042	-0.333	0.07274
MIR2052HG	3.0129	0.5857	GTF2IP9	1.1925	0.1366	TPMTP1	-0.334	0.08685
GTF2H2C	3.0042	0.6129	ENPP1	1.192	0.2444	IQCB1	-0.372	0.09571
KLF2P3	3.0002	0.4006	AMDHD2	1.191	0.2528	NDUFB9P2	-0.374	0.09698
C5AR2	3	0.5024	CTAGE6	1.19	0.2772	RAD21P1	-0.375	0.06521
KIR3DL1	2.995	0.392	SUPT3H	1.1893	0.2649	UBE2D3P3	-0.401	0.04929
MGAT4D	2.992	0.2504	TBC1D22A-AS1	1.188	0.1729	TPT1P6	-0.417	0.1042
C11orf98	2.986	0.5165	PCGF2	1.187	0.2158	PINX1-DT	-0.443	0.08281
GPR148	2.976	0.5619	PIGUP1	1.187	0.1232	PTTG4P	-0.458	0.1258
PP2672	2.976	0.3775	TTC30A	1.187	0.186	DESI1	-0.484	0.1267
CRLF2	2.971	0.5366	ZNF250	1.187	0.2402	CEBPG	-0.486	0.1225
KRTAP3-1	2.954	0.4655	ITSN1	1.184	0.1992	RN7SL237P	-0.492	0.08996
C19orf33	2.949	0.6623	LINC01342	1.181	0.2454	RPS26P18	-0.493	0.1203
CYP4F30P	2.948	0.2748	LRBA	1.181	0.2198	NDUFAB1P1	-0.499	0.1211
TBC1D26	2.947	0.6694	PCBP2	1.18	0.1144	EVX2	-0.5	0.06734
NOS1AP	2.9426	0.3375	ULK2	1.18	0.2279	LINC01149	-0.5	0.1054
RNF223	2.9314	0.4769	SYCE2	1.1795	0.2008	SBF1P2	-0.512	0.08414
ADRA2A	2.93	0.6175	IFT140	1.179	0.2286	LINC01526	-0.517	0.1487
ASTL	2.9291	0.4659	SNF8	1.178	0.2169	OR2A3P	-0.528	0.1078
NPIPB9	2.923	0.5314	SLC30A1	1.177	0.2332	RPL36AP45	-0.53	0.1454
USP17L1	2.923	0.3623	ZNF28	1.177	0.1794	LINC02300	-0.534	0.1195
GLYATL3	2.922	0.5798	NRAV	1.176	0.2069	MT-CO3	-0.54	0.147
LINC01087	2.92	0.6906	SF3B3	1.176	0.2777	OR4C49P	-0.543	0.1544
DRD5	2.915	0.5731	TMEM134	1.176	0.2568	DNM1P47	-0.552	0.1228
CCDC175	2.913	0.5262	NAB2	1.175	0.1638	ATP11AUN	-0.556	0.09153
DEFB124	2.897	0.549	PPIAP46	1.172	0.2795	ARIH2P1	-0.558	0.123
GSTA5	2.897	0.4418	ARHGEF28	1.17	0.2159	COX7A2P1	-0.568	0.141
TDH-AS1	2.89	0.3206	ZNF785	1.169	0.2315	PNRC1-DT	-0.584	0.1039
DGAT2L6	2.888	0.4695	KCTD13	1.168	0.1702	SETP12	-0.594	0.1141
C12orf50	2.885	0.399	SPICE1	1.163	0.1924	PDSS1P2	-0.618	0.08199
DYNLRB2	2.881	0.661	OR10D1P	1.162	0.1036	CICP13	-0.628	0.1421
HSFX1	2.88	0.4474	MTCO1P46	1.161	0.2064	KLF13	-0.643	0.143
PABPN1L	2.879	0.4681	GANC	1.16	0.2091	PGGT1BP1	-0.644	0.0979
CCL1	2.875	0.625	ZNF283	1.1591	0.2352	CLTB	-0.649	0.1316

FAM83E	2.8658	0.4421	SHISA5	1.159	0.2229	ERP29P1	-0.649	0.1837
C9orf92	2.864	0.4472	ZNF688	1.159	0.2398	RPS3AP53	-0.672	0.1514
UTF1	2.862	0.3809	ATP8B1	1.157	0.2663	RPL7P19	-0.674	0.1603
FAR2P3	2.8618	0.6389	RPL21P35	1.153	0.2525	BIN2P1	-0.691	0.104
FMN1	2.8615	0.237	TMEM144	1.153	0.2088	RPL17P1	-0.703	0.1643
MUC17	2.858	0.5608	ZBTB42	1.153	0.1656	TBX18-AS1	-0.707	0.1379
RBM20	2.8514	0.6061	AMZ2	1.152	0.2655	LINC01413	-0.731	0.07932
IGFN1	2.828	0.3612	LTBP3	1.152	0.2502	DPPA2P3	-0.737	0.1675
THBS1-IT1	2.826	0.4491	MACIR	1.151	0.1779	KRT18P64	-0.75	0.08909
PRAMEF8	2.816	0.4483	RPS23P1	1.151	0.209	LRP3	-0.754	0.1817
ARL17B	2.81225	0.4857	FAM102A	1.15	0.2559	PSMD7-DT	-0.755	0.08351
TMPRSS11A	2.811	0.2982	URM1	1.15	0.2049	LZTS1-AS1	-0.764	0.1675
KCNG4	2.806	0.3213	ANO7L1	1.1498	0.2593	S1PR2	-0.7644	0.1973
BFSP2	2.7986	0.5748	RPS9	1.148	0.2104	MCPH1-DT	-0.7689	0.1807
ENTPD8	2.787	0.5595	GPR137	1.146	0.2086	RPS10P9	-0.774	0.09992
SFTPA2	2.7789	0.7507	ALG11	1.144	0.1994	LINC00656	-0.78	0.1603
FFAR1	2.774	0.4218	ZNF544	1.144	0.1922	HIGD2AP2	-0.791	0.2072
KRT13	2.7685	0.5238	MCEMP1	1.143	0.3228	LINC02554	-0.798	0.146
NPIPA3	2.767	0.615	TEDC2	1.143	0.1772	RN7SL569P	-0.798	0.1761
FCMR	2.76	0.4917	IQCM	1.139	0.2542	TMEM242-DT	-0.799	0.1831
TMEM229B	2.7566	0.2304	ZNF613	1.138	0.2292	ASTN2-AS1	-0.8	0.1643
LINC01843	2.7559	0.4596	PRPSAP1	1.137	0.1991	AOX3P	-0.811	0.2314
LCN12	2.7448	0.3198	SLC19A2	1.137	0.2679	PDLIM1P1	-0.819	0.1648
KCNK15	2.732	0.5654	ARL6IP4	1.134	0.212	C9orf40	-0.8269	0.1825
RET	2.726	0.4814	CLDN34	1.134	0.2962	NAMPT	-0.829	0.1834
GATA3	2.723	0.3816	EIF1	1.134	0.2256	CDRT15P9	-0.837	0.231
IL19	2.714	0.565	KAT5	1.134	0.1604	OR52T1P	-0.858	0.1755
ANTXRL	2.713	0.7051	ZNF571	1.1314	0.207	TIGD2	-0.861	0.1903
CCDC74A	2.711	0.608	SRRM2-AS1	1.1305	0.2656	ARL4AP2	-0.863	0.1893
COX8C	2.707	0.7008	C1orf210	1.128	0.2888	UGP2	-0.865	0.1757
PIFO	2.7056	0.503	SKAP1-AS2	1.127	0.2642	ENPP7P9	-0.866	0.1478
BAK1P2	2.705	0.5302	NME3	1.126	0.2314	PGBD4P3	-0.876	0.2066
MAGEB16	2.703	0.2978	RARG	1.126	0.1804	TRBV4-2	-0.876	0.1987
ZG16B	2.69	0.4743	RALGPS2-AS1	1.125	0.2935	AKAP17BP	-0.879	0.1283
LINC01489	2.6889	0.4823	TMEM30B	1.125	0.2249	LINC02060	-0.89	0.2436
PCP2	2.674	0.4185	FOXO6	1.123	0.2107	MIR4290HG	-0.893	0.2327
GOLGA8T	2.672	0.4376	SIN3A	1.123	0.113	IL6-AS1	-0.894	0.2249
KRT37	2.665	0.5911	H2AC6	1.122	0.2742	ASLP1	-0.897	0.144
BPIFB4	2.649	0.4868	PRKAG1	1.122	0.2237	LINC00661	-0.897	0.2304
ERICH6B	2.636	0.6616	TOR2A	1.122	0.1503	CLCP2	-0.902	0.1995
C5orf49	2.6351	0.4377	ZBTB41	1.121	0.2476	TRAV25	-0.903	0.2274
KCNMB2	2.628	0.444	ZNF484	1.1182	0.2526	IFNL4	-0.91	0.1766
SLC44A4	2.623	0.5556	ZNF468	1.118	0.1518	RN7SL793P	-0.913	0.1773
TEX36	2.618	0.6243	FAM102B	1.116	0.2401	TRBV4-1	-0.913	0.1967
C8orf34-AS1	2.6164	0.4751	KCNH1-IT1	1.116	0.3021	TRBV3-1	-0.919	0.2276
CCDC57	2.615	0.5184	ABT1P1	1.113	0.1482	NBPF8	-0.9278	0.1986
GLRA4	2.614	0.3625	DIS3L	1.111	0.1866	LINC02254	-0.937	0.2273
DNAAF3	2.6043	0.4508	TRIM8-DT	1.11	0.2213	SACS-AS1	-0.944	0.2333
PRR32	2.599	0.3799	KMT2D	1.109	0.2295	WDR88	-0.946	0.2514
GPR37L1	2.5987	0.5685	MTND5P25	1.109	0.2174	IL6R-AS1	-0.955	0.2173
SRARP	2.5961	0.6009	ZNF431	1.109	0.1732	IMPDH1P7	-0.961	0.1183
MSLNL	2.59	0.637	LINC02571	1.108	0.2525	GRM5-AS1	-0.962	0.1713
ADORA1	2.585	0.4822	SESTD1	1.107	0.222	HNRNPA1P66	-0.97	0.1711
RBM11	2.5799	0.5675	SIVA1	1.106	0.2486	TRAV8-2	-0.974	0.2513
TEX19	2.575	0.4971	ZNF326	1.106	0.2513	VDAC2P3	-0.974	0.1812
MYB	2.566	0.5514	LINC01569	1.1058	0.249	TRAV23DV6	-0.975	0.2037
RHOH	2.5511	0.5662	DGLUCY	1.1	0.2201	PPM1L-DT	-0.976	0.2311
GOLGA6L4	2.544	0.5756	STAT6	1.1	0.2507	LINC02457	-0.977	0.1694
TMPRSS6	2.5385	0.6361	NDUFB4P1	1.099	0.2628	MALT1-AS1	-0.978	0.2163
LONRF2	2.5379	0.4805	FBXO36	1.0984	0.2144	PTX4	-0.996	0.2735
FOXN1	2.5367	0.5188	SHTN1	1.097	0.1818	LINC02401	-1	0.1782
CAPS2	2.5341	0.279	ZNF619	1.0961	0.151	MSX2P1	-1.003	0.1755
RPL17	2.528	0.4118	ZNF681	1.0958	0.2312	FAM215A	-1.004	0.1824
TAS2R1	2.519	0.4228	IMPDH1P4	1.095	0.1621	FAM3D-AS1	-1.006	0.1962
GSTM2	2.513	0.5678	LINC01363	1.095	0.1963	H3Y1	-1.006	0.2706
SLC39A6	2.513	0.4387	RNPS1	1.094	0.1105	IGHV3-69-1	-1.00615	0.2459
RERG	2.512	0.4129	CHID1	1.091	0.2093	RN7SKP78	-1.007	0.2663
IRS1	2.502	0.3042	TTC30B	1.091	0.1789	ZDHHC4P1	-1.007	0.2741
SPTBN4	2.5	0.4933	HIF1AN	1.09	0.1971	TUBAP8	-1.024	0.2843
NFE2	2.499	0.5399	ZNF578	1.09	0.2735	LIX1-AS1	-1.025	0.1208
ADAMTSL5	2.490478	0.3374	ABCB8	1.089	0.1448	TPT1P12	-1.026	0.1848
MKX-AS1	2.4748	0.2612	PTRH1	1.089	0.221	BNIP3P38	-1.028	0.2118
ALOX12P2	2.473	0.5378	ZNF559	1.089	0.2489	LINC02266	-1.031	0.2773
CXCR5	2.4706	0.6175	ZKSCAN2	1.08866	0.2146	RN7SL577P	-1.031	0.1974
GH1	2.47	0.4303	TMA16	1.088	0.2315	HSD11B1-AS1	-1.032	0.1642
ANKRD20A1	2.462	0.4224	SULT1A1	1.086	0.2568	VN1R34P	-1.034	0.2275

CRIP1	2.445	0.5038	ZNF483	1.0842	0.2614	LUARIS	-1.039	0.2815
OR6B2	2.444	0.3479	ERBB3	1.084	0.2675	SUCLA2P1	-1.039	0.1378
CCDC170	2.4436	0.4988	HNRNPA1P71	1.083	0.08888	TRBV15	-1.042	0.1521
PRRT1B	2.442	0.6088	IMMP2L	1.083	0.2276	PPIAP45	-1.043	0.1288
TRIM61	2.441	0.5786	POLD4	1.083	0.2235	LINC02694	-1.052	0.1326
IFNL2	2.432	0.6395	RALGAPA1P1	1.082	0.2355	ABHD17AP5	-1.055	0.2736
THSD4	2.426	0.2642	ZDHHC17	1.082	0.2008	LINC01099	-1.056	0.181
PLCD3	2.424	0.4412	COQ7	1.081	0.2192	LINC00456	-1.062	0.1387
MISP	2.418	0.4681	ARID2	1.078	0.1757	TRAV1-1	-1.065	0.2274
FGD3	2.416	0.403	CA15P1	1.076	0.1356	SUCLA2-AS1	-1.067	0.1909
TANC2	2.414	0.3	WASIR1	1.074	0.1915	PFN1P2	-1.0695	0.2311
DCDC1	2.4107	0.4924	ZNF558	1.074	0.1729	NCF1C	-1.06965	0.2813
ANXA9	2.408	0.3673	MEAF6	1.073	0.1908	RPS12P17	-1.076	0.1671
EFHB	2.396	0.491	AFF1	1.072	0.2143	NACAP8	-1.081	0.1918
RSL24D1P8	2.396	0.2871	SMIM7	1.07	0.222	KRT18P8	-1.082	0.144
GFRAL	2.394	0.4576	ZKSCAN3	1.0693	0.1915	NUDT19-DT	-1.0857	0.2426
FRG2	2.393	0.2223	BTF3P1	1.068	0.1206	LINC01645	-1.09	0.2512
HTR4	2.391	0.3023	ZNF623	1.068	0.2353	PPIAP35	-1.093	0.2976
DELEC1	2.373	0.422	ATF7IP	1.067	0.1778	ZNF37CP	-1.0932	0.09632
SLC25A53	2.3719	0.3398	CCDC24	1.067	0.1916	RN7SL280P	-1.094	0.2298
EPHA10	2.3698	0.5897	DAZAP2	1.066	0.1889	KCNIP1-OT1	-1.099	0.2667
LINC02130	2.3607	0.4556	NT5DC1P2	1.065	0.256	PSG8-AS1	-1.101	0.2911
SULT1A4	2.351	0.4914	DGKK	1.064	0.2895	LINC02446	-1.103	0.2536
C4A	2.35	0.4928	CTBP1	1.063	0.2051	MTND5P32	-1.103	0.2864
MLPH	2.35	0.468	ZNF799	1.0602	0.1514	CYB5R2	-1.106	0.2098
TMEM132C	2.349	0.4697	HDAC2	1.06	0.2456	MTATP6P13	-1.107	0.1555
FOXI2	2.342	0.5617	OR5P1P	1.058	0.2352	FAM230G	-1.116	0.2319
NACAP10	2.337	0.1081	WDFY3	1.058	0.1727	GCNT1P5	-1.118	0.1908
CASKIN1	2.3364	0.5489	ATRIP	1.055	0.1813	RN7SKP56	-1.123	0.1106
C5orf63	2.3341	0.3761	BTF3P7	1.055	0.223	PRR34	-1.1246	0.2712
OR11H4	2.321	0.2425	FBXO22	1.055	0.2277	H3P33	-1.128	0.1628
C12orf42	2.29	0.579	PROSER3	1.055	0.2047	MTCYBP23	-1.143	0.2504
IFITM10	2.2814	0.4631	PCBP2P2	1.0549	0.1854	OR2T32P	-1.145	0.213
DYNAP	2.277	0.4957	PUS1	1.052	0.2205	PSMD10P2	-1.148	0.2227
RIMBP3C	2.274	0.3711	ZNF347	1.052	0.1688	CFAP61-AS1	-1.151	0.1784
C1orf226	2.264	0.3193	ATP6V0C	1.051	0.2552	LINC01985	-1.155	0.22
NACA2	2.261	0.57	C2orf15	1.049	0.21	IGFL2-AS1	-1.16	0.3245
TMEM232	2.25375	0.4418	HOOK1	1.049	0.1917	FRG1GP	-1.162	0.1278
SLC14A2-AS1	2.252	0.2979	LMLN	1.049	0.1429	USP12P2	-1.166	0.2794
ANKS1B	2.2507	0.3079	R3HDM2	1.049	0.1217	ZEB2P1	-1.173	0.1963
ELK2BP	2.244	0.3513	KIAA0895	1.046	0.2161	HMGB1P35	-1.174	0.3014
SPATA17	2.2413	0.5217	MFSD3	1.044	0.2482	LINC00426	-1.177	0.3
ATOX1	2.232	0.3938	NFS1	1.043	0.1911	VDAC1P13	-1.178	0.1487
NKX2-6	2.223	0.465	RMDN2-AS1	1.043	0.2757	LINC02556	-1.18	0.1896
PNPLA5	2.223	0.516	CASP7	1.042	0.1672	MTCO1P39	-1.182	0.1574
ZNF552	2.218	0.2817	MIEF2	1.042	0.1838	CDK6-AS1	-1.184	0.2527
SYNPR	2.217	0.4046	RFX7	1.041	0.1592	LINC01686	-1.195	0.2617
ZNF280A	2.217	0.3961	TCEAL3	1.04	0.2781	ZNF859P	-1.202	0.1894
EIF4A1P7	2.215	0.2852	FXN	1.039	0.1891	AIRN	-1.203	0.2478
SLITRK4	2.207	0.5157	CDK12	1.038	0.1652	WDR82P2	-1.203	0.2549
UBXN10	2.2062	0.3887	CFL1P2	1.038	0.1335	MRPS15P1	-1.206	0.2203
RPL37A	2.2	0.3812	ZNF780A	1.037	0.199	RPL5P8	-1.208	0.2221
LRRC3C	2.196	0.4238	RN7SL559P	1.0367	0.2195	HNRNPA1P34	-1.21	0.1254
BCL2	2.195	0.495	PRR13	1.035	0.2009	IL12A-AS1	-1.221	0.2575
PIGP	2.195	0.4008	THAP6	1.034	0.1285	HSD3BP3	-1.227	0.07525
PLAAT2	2.1892	0.512	RUNDC1	1.033	0.2122	LINC00244	-1.228	0.1602
TTC6	2.1866	0.539	LCMT1	1.032	0.1867	LINC02693	-1.2285	0.2975
H3C10	2.168	0.4412	CLBA1	1.031	0.1898	BAALC-AS2	-1.231	0.341
MAPK15	2.165	0.3522	HOMEZ	1.031	0.2217	LINC01562	-1.233	0.217
ACOX2	2.163	0.4807	ADAL	1.029	0.2383	C2orf88	-1.2347	0.2927
OTUD6A	2.16	0.3541	ZSCAN26	1.029	0.2252	OPA1-AS1	-1.235	0.2896
CRACR2A	2.158	0.4158	SDAD1P2	1.028	0.1175	LPIN1	-1.254	0.2622
LMX1B	2.155	0.3894	CLASP2	1.026	0.1867	ADGRD2	-1.265	0.3343
FOXD3-AS1	2.148	0.4411	RBM12B	1.026	0.1834	SP9	-1.274	0.239
SFXN2	2.14	0.4112	TRIM44	1.025	0.13	DTNB-AS1	-1.276	0.1495
KRT35	2.133	0.4402	ZNF610	1.0235	0.2301	C4orf50	-1.279	0.2667
C15orf40	2.132	0.3321	BRWD1	1.023	0.1871	HSPB2-C11orf52	-1.2804	0.2586
NPIPB7	2.132	0.4714	RALGAPB	1.022	0.1938	TUBBP6	-1.281	0.3434
DSCAML1	2.128	0.4802	ZNF641	1.021	0.1667	NOS2P3	-1.29	0.1854
FAM204A	2.127	0.3042	CSNK1G1	1.02	0.1152	CST13P	-1.292	0.3301
KHDC1L	2.122	0.4329	ZNF775	1.019	0.2608	MYHAS	-1.301	0.3415
MTRNR2L4	2.122	0.4626	RAB11FIP3	1.016	0.2012	AMMECR1-IT1	-1.302	0.2785
ABCA4	2.12	0.3465	HNRNPF	1.015	0.1842	PXDC1	-1.303	0.1811
C9orf116	2.115	0.3009	TRIM34	1.015	0.1723	CHST2	-1.3101	0.3253
CCDC185	2.115	0.4161	C14orf119	1.014	0.2206	BMP6	-1.3117	0.3299
TAS2R39	2.115	0.2132	MAP4K2	1.013	0.2395	BNIP3P24	-1.312	0.3359

RETREG1	2.112	0.3144	EIF4A1P1	1.012	0.2184	NPM1P23	-1.316	0.2375
TSPY26P	2.1115	0.2469	PHAX	1.012	0.1401	BNC2-AS1	-1.319	0.1756
STARD5	2.1096	0.395	NF1	1.011	0.1714	CCNE1	-1.3201	0.2644
CLRN1	2.106	0.3908	TAF9	1.011	0.2328	LSINCT5	-1.322	0.2781
HDAC11	2.106	0.2475	NUP107-DT	1.01	0.1874	HMGN3-AS1	-1.323	0.2073
RPS29P16	2.1017	0.3971	CCDC171	1.0095	0.2086	NUDT8	-1.323	0.3054
PREX1	2.099	0.3994	RPL7P47	1.009	0.2249	RAD51AP2	-1.324	0.2793
TBC1D9	2.092	0.3211	KRT18P22	1.007	0.192	ARHGAP19-SLIT1	-1.326	0.3208
NBPF1	2.091	0.2956	TRIM66	1.0062	0.192	KRT223P	-1.333	0.3803
NPNT	2.088	0.325	AP2B1P1	1.006	0.2326	MARCHF10-DT	-1.333	0.07274
NYAP2	2.087	0.4846	ZNF816	1.006	0.1898	PARP11-AS1	-1.333	0.1841
CCDC103	2.0848	0.3302	GALNT4	1.005	0.1471	RNF145	-1.335	0.2744
SH3D21	2.084	0.4614	VSIG10	1.005	0.2402	YBX1P6	-1.336	0.3214
VDAC1P7	2.083	0.08966	ZNF84	1.004	0.2137	LINC02730	-1.339	0.1145
SCTR-AS1	2.078	0.2989	CRAMP1	1.003	0.1596	JAKMIP1-DT	-1.342	0.281
C5orf47	2.077	0.4007	SAMD4B	1.003	0.2129	CDK6	-1.3537	0.319
PRR15	2.075	0.5211	RN7SL78P	1.0025	0.1642	UBE2V1P13	-1.354	0.1265
SCNN1A	2.074	0.4468	GPD1L	1.001	0.2114	COX5BP7	-1.36	0.3422
SLC29A3	2.067	0.219	BBS1	0.999	0.1819	TRAV12-3	-1.362	0.2456
LRRC37A	2.064	0.3724	CYB561A3	0.998	0.2619	NCKAP5-AS1	-1.363	0.3032
TREML4	2.064	0.2843	STRADA	0.998	0.1828	PHBP7	-1.367	0.1862
BLACE	2.062	0.2993	APEH	0.997	0.1672	SMIM28	-1.374	0.3902
NACA	2.059	0.3215	ATOX1-AS1	0.997	0.2159	PLA2G4A	-1.3742	0.3446
RAPGEFL1	2.058	0.3913	SF1	0.991	0.1439	RPL21P90	-1.376	0.2295
KRTAP5-10	2.055	0.4657	NIPA1	0.99	0.1599	CELF2-AS1	-1.38	0.3367
CCDC169	2.0546	0.481	CBX3P1	0.989	0.1651	AFDN-DT	-1.3824	0.2893
RAPH1	2.048	0.3228	RILPL1	0.989	0.1844	FAM89A	-1.3848	0.3366
TTC8	2.046	0.1825	TADA2A	0.989	0.1796	MIR99AHG	-1.3854	0.2842
SLC24A3-AS1	2.043	0.4392	TPRN	0.988	0.1968	FTH1P22	-1.386	0.3248
RIMKLA	2.0427	0.4683	OTUB2	0.9867	0.2278	IRAIN	-1.386	0.2532
IQCK	2.039	0.2847	GHDC	0.985	0.2357	RN7SL30P	-1.407	0.2045
NAIP	2.023	0.4057	HNRNPCP8	0.985	0.2264	RNASEH1P1	-1.411	0.2638
RAB26	2.021	0.2963	CDS1	0.984	0.2288	LIME1	-1.414	0.337
ZBTB44-DT	2.021	0.2194	EIF4E3	0.984	0.1983	GSPT2	-1.4162	0.333
FRS2	2.016	0.3062	PWWP2A	0.984	0.1466	IGLV10-54	-1.4163	0.2377
MRGPRX3	2.015	0.4403	INO80-AS1	0.983	0.1581	NECTIN3	-1.4199	0.2895
NANOGP8	2.013	0.4984	CHTOP	0.982	0.1422	LIX1L	-1.4235	0.3226
KCNJ11	2.0081	0.5083	LRRC57	0.982	0.1888	NDUFA3P2	-1.426	0.2113
S100G	2.005	0.4548	FAM219B	0.98	0.2337	GK3P	-1.431	0.1686
NRIP1	2.004	0.2623	OR2W2P	0.98	0.2532	RN7SL354P	-1.431	0.2084
AGBL2	2.0031	0.4578	TSPAN13	0.979	0.2604	LINC02086	-1.434	0.3612
ATCAY	2.001	0.4218	BTRC	0.978	0.1787	LINC00402	-1.45	0.2939
HBM	1.995	0.4987	LINC02710	0.978	0.04463	ADAMTS3	-1.453	0.3218
KNCN	1.994	0.461	FAF1	0.976	0.2099	LINC01559	-1.454	0.2875
EVA1B	1.992	0.3592	SRSF10	0.975	0.1715	RRAGD	-1.4555	0.334
MAGEB18	1.99	0.4452	SIGIRR	0.974	0.1965	TRAV26-2	-1.456	0.178
SMAD2	1.987	0.2884	WFS1	0.973	0.2312	RPL31P7	-1.457	0.1539
C12orf60	1.97919	0.2876	ZNF675	0.973	0.217	ZNF75BP	-1.458	0.2022
OR51Q1	1.975	0.3403	CRIP2	0.971	0.2491	MRPS9-AS2	-1.469	0.1604
CHCHD3P1	1.9742	0.3653	ZNF101	0.971	0.1668	PCMTD1P3	-1.474	0.282
ACADSB	1.971	0.317	BZW1P2	0.9709	0.1621	LINC01460	-1.479	0.2509
RPS23	1.97	0.3449	AKIP1	0.97	0.1827	HNRNPA3P2	-1.48	0.3302
IL6ST	1.967	0.3516	ZNF281	0.969	0.2002	LINC02186	-1.48	0.1578
OR51B5	1.967	0.3393	ZSCAN5B	0.969	0.2691	LINC02507	-1.482	0.2903
INPP4B	1.9586	0.4266	ZBTB8A	0.968	0.2192	NATP	-1.483	0.095
F7	1.9556	0.4869	ZNF497	0.9669	0.217	LRRC2-AS1	-1.484	0.354
TAC3	1.954	0.4064	ZNF517	0.966	0.1665	HMGN1P2	-1.487	0.3507
NQO1	1.953	0.3635	SLC38A9	0.965	0.1919	TRBV2	-1.487	0.2894
KRT19	1.952	0.4122	BLOC1S5	0.964	0.1326	TRAV6	-1.49	0.1752
SLC35E3	1.95	0.4129	TMEM234	0.964	0.1607	RPS18P13	-1.493	0.3711
GAL3ST3	1.945	0.3824	TMEM69	0.963	0.1448	ANXA2P1	-1.494	0.2931
KSR2	1.944	0.3348	PDCD6IP	0.962	0.1884	MBNL1-AS1	-1.4946	0.2705
LINC00475	1.9403	0.3929	LCORL	0.9615	0.2049	COL4A2-AS2	-1.497	0.2062
ADCY9	1.94	0.2226	GAPVD1	0.961	0.1422	TRAV21	-1.498	0.3645
HNRNPLP1	1.937	0.1834	TCEA3	0.961	0.198	CD200R1L-AS1	-1.502	0.3723
ERCC6L2	1.936	0.2392	ZNF519	0.96	0.2577	OR7E115P	-1.505	0.202
PLIN5	1.934	0.4311	COPS2	0.958	0.1993	TRBC2	-1.505	0.3571
TOX3	1.933	0.4999	RPS29P5	0.956	0.1631	TRAV8-3	-1.51	0.2843
OR1M1	1.928	0.3755	HSPA4	0.954	0.1715	RNF186	-1.514	0.4134
PPIAP55	1.923	0.3205	RCHY1	0.952	0.1821	LINC02723	-1.516	0.2111
IGHE	1.9174	0.4205	COX11	0.95	0.1457	SP5	-1.523	0.4127
NRBF2P1	1.915	0.4001	HAUS3	0.95	0.1816	TNFSF12-TNFSF13	-1.5237	0.3203
DBNDD2	1.91	0.3609	ERCC4	0.949	0.1879	LINC02433	-1.53	0.2879
ZNF587	1.908	0.203	PTMS	0.945	0.1876	CLMAT3	-1.532	0.2751
ZSCAN4	1.908	0.4832	SDHAF2	0.945	0.2345	IGHV6-1	-1.5449	0.4214
H2BC7	1.9059	0.3484	TMEM116	0.945	0.2196	RNU6ATAC35P	-1.545	0.1594

GABRG3	1.905	0.3978	RPL14P6	0.942	0.201	LINC01854	-1.558	0.3328
EVPLL	1.904	0.483	TLE3	0.942	0.231	EGFR-AS1	-1.561	0.246
LINC01962	1.899	0.2466	ZNF587B	0.942	0.2158	ANKRD33B	-1.565	0.396
CTNNA3	1.897	0.3721	FGF7P8	0.9391	0.212	ODAD2P1	-1.571	0.4035
RPL17P34	1.897	0.1716	AKT1S1	0.939	0.1837	PRDX2P4	-1.575	0.4106
DACH1	1.8953	0.4781	RPL6P9	0.935	0.07661	KCNJ4	-1.585	0.4076
TRIM40	1.895	0.4645	SLC22A18	0.935	0.2167	LURAP1L-AS1	-1.588	0.2579
ZFYVE28	1.8938	0.3957	SPDYE3	0.935	0.1679	FAM230C	-1.594	0.4495
CFB	1.889	0.3943	ARHGEF26	0.934	0.2145	ZNF710-AS1	-1.5975	0.3564
BRF1	1.888	0.2728	ARMCX6	0.934	0.1855	TBCAP2	-1.602	0.2286
CALHM1	1.887	0.4209	ANAPC7	0.931	0.2053	NCF4-AS1	-1.606	0.394
RDM1P3	1.886	0.2214	C1D	0.931	0.2331	TMCC3	-1.6066	0.3604
LRRC73	1.88214	0.3904	MTCO1P4	0.931	0.1371	ROR1-AS1	-1.61	0.2594
CYP2G1P	1.881	0.3572	KLHL11	0.9297	0.1422	TRBV6-1	-1.61	0.3319
KLHDC9	1.876	0.3835	RTKN2	0.9291	0.2365	DIPK1C	-1.625	0.3699
OR2AG2	1.876	0.2814	RPS2P46	0.929	0.1553	LINC01620	-1.63	0.3909
OR10A2	1.875	0.2721	CEP120	0.927	0.1826	AIMP1P1	-1.636	0.2062
BAIAP3	1.8744	0.3959	ZNF546	0.92594	0.2226	IGKV1D-16	-1.63848	0.4356
RN7SKP259	1.874	0.1499	BTBD17	0.924	0.2533	MATR3	-1.643	0.3758
CBWD3	1.87	0.5143	FAM174A	0.924	0.1561	TRAV12-1	-1.6478	0.3264
MS4A10	1.866	0.2468	AP1AR	0.922	0.1402	HLX-AS1	-1.648	0.1911
H2BC15	1.8628	0.2672	ATXN7	0.921	0.1377	FYTTD1P1	-1.652	0.4274
IPO8P1	1.861	0.3937	TOP3BP1	0.92	0.1929	CHST3	-1.6604	0.3936
ECI1	1.854	0.331	EXOC7	0.918	0.2045	NETO1-DT	-1.661	0.3299
C15orf48	1.85	0.3813	SMAD3	0.918	0.2032	OVCH1-AS1	-1.663	0.1505
PSORS1C1	1.8467	0.3993	TET2	0.918	0.1969	LINC02308	-1.665	0.2897
KRT38	1.846	0.3279	RAB40C	0.917	0.1182	TRAV19	-1.676	0.2954
DIABLO	1.843	0.3205	C1GALT1P2	0.916	0.1479	ADORA2BP1	-1.685	0.2892
CDYL2	1.8407	0.3154	NHLRC1	0.916	0.2169	IKZF3	-1.6855	0.3713
IGFBP4	1.84	0.2592	CRYZL1	0.915	0.1737	LINC01208	-1.686	0.4082
ZNF91	1.837	0.2936	GOT2P2	0.912	0.119	TRAC	-1.69	0.3476
NARS1P2	1.831	0.2915	MFSD5	0.912	0.1227	TRAV8-1	-1.69	0.206
CMC1	1.823	0.3473	UROS	0.91	0.2131	PDE9A	-1.6971	0.3898
LINC00298	1.818	0.2184	SMG1P5	0.9087	0.1745	TRAV16	-1.704	0.1731
DMKN	1.816	0.459	POLL	0.908	0.1907	WNT10B	-1.705	0.4194
TCTN1	1.815	0.1983	ZNF782	0.9041	0.1316	HRCT1	-1.7192	0.3832
KRT74	1.812	0.3627	RPRD2	0.903	0.171	SYT10	-1.734	0.3639
ZNF665	1.8111	0.2479	SLMAP	0.902	0.163	HOXD13	-1.745	0.2061
FTH1	1.81	0.294	RN7SKP249	0.901	0.1717	TRBV5-6	-1.748	0.1771
FAUP1	1.8058	0.2685	GDPGP1	0.899	0.154	ZFPM2-AS1	-1.749	0.332
RIPOR3-AS1	1.805	0.4396	NFE2L1-DT	0.8984	0.1552	PPP1R2C	-1.756	0.09211
TGIF1	1.805	0.2778	RPS2P4	0.898	0.2366	MIR4500HG	-1.76	0.2311
CTAGE1	1.797	0.3731	TMUB2	0.898	0.1932	IGLV3-9	-1.7605	0.4328
KIAA0825	1.7945	0.2572	DDAH2	0.896	0.1812	EMILIN3	-1.762	0.3586
RPS27P25	1.7943	0.3799	DDX52	0.894	0.1844	ADGRG2	-1.763	0.3238
OVOL1	1.794	0.3822	MSL1	0.893	0.1842	LINC01114	-1.763	0.3291
L3MBTL1	1.7919	0.3605	ZNF766	0.893	0.1486	TRAV26-1	-1.786	0.2748
MBD5	1.791	0.2296	YWHAQP4	0.891	0.07162	STOX2	-1.7914	0.3861
C17orf50	1.79	0.4195	RNF169	0.89	0.1782	LINC00861	-1.795	0.3797
DNAJC8P1	1.788	0.1138	LARP4	0.888	0.1774	BCAR4	-1.804	0.3089
SLC9A3R1-AS1	1.788	0.4306	ZBTB8OS	0.888	0.189	PLCXD3	-1.814	0.4169
RNF213	1.787	0.3772	FPGT	0.887	0.1811	FGF14-AS2	-1.8153	0.3229
IFNB1	1.784	0.4397	CAPZA2	0.884	0.1986	MGC27382	-1.82	0.3157
SYT12	1.779	0.3238	PRRC1	0.883	0.1325	PGM5-AS1	-1.828	0.3775
CXXC5	1.778	0.1918	TRIM69	0.882	0.2064	TRGV9	-1.835	0.3646
ZNF528	1.776	0.2714	FOXJ3	0.88	0.1119	LINC00707	-1.836	0.3532
LYRM4	1.775	0.3396	NUDT17	0.88	0.2091	SYCE1L	-1.8365	0.3678
ABAT	1.774	0.4555	PACRGL	0.879	0.1853	GABRQ	-1.843	0.4666
KCTD6	1.771	0.2803	RALGAPA1	0.879	0.2089	PDHA1P1	-1.843	0.3402
UICLM	1.771	0.4009	RPS5P3	0.879	0.08626	LINC01625	-1.844	0.4119
FAM47A	1.76	0.4461	SP3	0.878	0.1208	H4C6	-1.847	0.2948
RPS6KL1	1.7553	0.342	ETV5-AS1	0.876	0.1518	LINC02084	-1.848	0.3733
MRPL42	1.753	0.3677	GAPDHP33	0.874	0.1411	EIF2S2P2	-1.85	0.3067
RPS27L	1.752	0.3105	ATXN2L	0.873	0.158	TCHH	-1.855	0.4709
AZI2	1.748	0.2478	ZNF432	0.871	0.1691	LINC01055	-1.862	0.4243
TRAPPC2L	1.745	0.3636	TTC3	0.87	0.16	PSMC1P9	-1.862	0.2256
TMEM120B	1.743	0.3098	ZNF3	0.87	0.1737	HAUS1P1	-1.863	0.2151
RAD51B	1.742	0.2917	TIGD6	0.869	0.1771	SMIM36	-1.863	0.3928
TRAPPC5	1.741	0.3618	ZNF234	0.869	0.1675	TRAV8-6	-1.871	0.2514
LINC01271	1.7405	0.3436	ZNF100	0.868	0.2003	PCDHB6	-1.873	0.4231
H2BC5	1.74	0.2156	BRD2	0.867	0.2058	LINC00857	-1.884	0.4121
OR51J1	1.736	0.3812	ZNF646	0.867	0.1694	NPM1P40	-1.899	0.4438
WFDC10B	1.735	0.4308	ADSL	0.866	0.2181	IL34	-1.9016	0.4212
EEF1B2P2	1.725	0.09747	ASB8	0.866	0.0972	FER1L6-AS2	-1.904	0.4203
ZNF814	1.725	0.3408	CUTALP	0.866	0.2273	KRT17P4	-1.908	0.3071
SLC16A5	1.719	0.3474	RPSAP45	0.865	0.1665	GLYATL1B	-1.911	0.2853

SMN2	1.716266	0.4841	DENND2D	0.864	0.2059	MATN1	-1.934	0.454
RALGPS2	1.716	0.2553	RHOBTB1	0.86	0.1671	LINC00937	-1.942	0.4141
DNAAF11	1.709	0.3408	ABHD16A	0.859	0.1453	NXPH4	-1.9456	0.416
LINC00871	1.709	0.2604	WDR41	0.859	0.2028	MIR3681HG	-1.947	0.2707
CMYA5	1.7054	0.3251	GDAP2	0.858	0.1425	TRBC1	-1.9494	0.458
NAP1L1	1.705	0.2816	ZNF774	0.8576	0.2032	ARL4AP1	-1.953	0.2153
CCDC30	1.70487	0.2594	ERCC6	0.857	0.161	SBSPON	-1.9585	0.4566
FAM187A	1.70166	0.4059	SPINT2	0.857	0.2159	IL22RA2	-1.96	0.4621
PRR36	1.7	0.4107	GNPDA2	0.856	0.2078	SNHG26	-1.9605	0.2565
PMEL	1.69836	0.3832	PAGR1	0.855	0.1946	YBX2P2	-1.962	0.4266
CFAP44	1.6981	0.3399	RAPGEF6	0.855	0.164	LINC01583	-1.966	0.208
TUBBP5	1.6945	0.4227	SERTAD3	0.854	0.1934	FOXC1	-1.9662	0.498
ZNF771	1.694	0.2849	SLC4A8-AS1	0.854	0.1022	MTND2P16	-1.973	0.3957
HSPB1P2	1.6934	0.3782	SMG1P1	0.854	0.2215	TRBV29-1	-1.984	0.3507
EFCAB2	1.69	0.39	DDX23	0.853	0.1322	LINC00460	-1.988	0.4682
IGF1R	1.69	0.3804	SEPHS1P6	0.853	0.1928	LINC02099	-1.99	0.398
SEC23A-AS1	1.687	0.2285	ZNF629	0.849	0.1679	MT1X	-1.991	0.3947
UCP2	1.685	0.273	AGFG1	0.848	0.1802	LINC01819	-1.994	0.4585
ERI2	1.682	0.2036	OR2L1P	0.847	0.1754	LINC01886	-1.996	0.06288
BEST1	1.6814	0.2543	RAMACL	0.847	0.1349	ALKAL2	-1.9971	0.4228
DNAI7	1.6781	0.367	ARIH2	0.846	0.1408	ACE2	-2.006	0.4991
EMID1	1.678	0.3567	E4F1	0.844	0.1479	MIR3142HG	-2.007	0.4741
TMEM63C	1.678	0.3696	MTA1	0.844	0.1855	SETP21	-2.01	0.2218
NCBP2-AS1	1.677	0.263	RPS3AP49	0.844	0.19	TBC1D3D	-2.015	0.4246
SHC1P2	1.676	0.3538	ZNF16	0.844	0.1897	PLA2G4E	-2.029	0.3891
ZNF703	1.675	0.3438	ATF6B	0.842	0.2156	PPIAP10	-2.029	0.3486
AADACP1	1.673	0.3313	ZNF566	0.842	0.1827	LINC01300	-2.034	0.1837
PARD6B	1.672	0.3456	RNF135	0.841	0.1401	LINC00668	-2.047	0.3082
MRPS23	1.67	0.3459	MFRP	0.84	0.1971	LINC02422	-2.05	0.1928
PTP4A2	1.67	0.2049	SCAI	0.8396	0.1829	PRKCQ-AS1	-2.05833	0.4674
SALL2	1.663	0.3021	PBX2	0.839	0.1717	FRG1CP	-2.0686	0.3893
YWHAEP7	1.66	0.3838	IQCC	0.838	0.1233	RN7SL359P	-2.0699	0.2075
PIP5KL1	1.6591	0.3934	VPS52	0.838	0.1719	PARM1-AS1	-2.072	0.2146
AK8	1.659	0.3733	ZNF329	0.838	0.187	TRHDE-AS1	-2.072	0.2732
CELSR1	1.659	0.3723	COG7	0.837	0.1193	TRBV5-1	-2.077	0.304
ACOT11	1.6577	0.3722	NCOA6	0.837	0.1735	TOX	-2.078	0.4935
LINC02298	1.6508	0.4167	ZNF761	0.836	0.1389	LINC01370	-2.0828	0.4228
KLRF2	1.648	0.4297	SVIP	0.835	0.2006	NDUFB4P11	-2.096	0.4727
LCN1	1.647	0.3868	DPY19L2P5	0.833	0.2046	NME1-NME2	-2.104	0.4513
NLRP7	1.647	0.3131	ZNF615	0.832	0.1746	CT75	-2.11	0.4154
MAP6D1	1.645	0.2413	SRC	0.831	0.1884	CCNB1IP1P3	-2.111	0.2822
PPM1B-DT	1.644	0.136	SAP30L	0.829	0.1667	VSTM5	-2.111	0.4875
TMEM184A	1.644	0.3003	GLYR1	0.827	0.1214	NIBAN1	-2.1123	0.3863
ZNF620	1.6432	0.185	ZNF181	0.827	0.1691	SAMMSON	-2.121	0.2113
OR13E1P	1.643	0.1656	ZNF721	0.826	0.191	LINC02635	-2.1269	0.4883
ARIH1	1.641	0.2212	ASXL1	0.824	0.171	LINC02006	-2.127	0.1961
CNTN4-AS1	1.638	0.1293	USP32	0.824	0.1812	LINC02487	-2.137	0.3753
ARMCX4	1.6352	0.3436	NECTIN2	0.823	0.1985	TRBV20-1	-2.1385	0.3508
LINC01490	1.633	0.2023	SYNRG	0.821	0.1544	KRT79	-2.14	0.5575
MRAP	1.633	0.2235	MARVELD2	0.82	0.1961	PLCE1-AS1	-2.14	0.4558
ZNF844	1.629	0.1715	BTD	0.819	0.1568	TRAV4	-2.141	0.2184
VSTM1	1.628	0.4493	CD2BP2	0.817	0.158	ZNF667-AS1	-2.1483	0.4363
CENPP	1.6264	0.2682	PXK	0.817	0.1467	BCL11B	-2.155	0.5287
RPL14	1.625	0.2341	ZNF747	0.817	0.1886	RN7SKP97	-2.16	0.144
MLLT3	1.624	0.2754	CIPC	0.816	0.1581	IGKV2D-29	-2.162	0.4274
KRT8	1.621	0.2505	RN7SL487P	0.816	0.2223	HSPB2	-2.1712	0.4824
PYCARD	1.619	0.3215	RFT1	0.814	0.1608	TSPEAR	-2.172	0.3181
H2AP	1.618	0.2529	PACSIN3	0.813	0.1777	HAPLN3	-2.1821	0.5362
S1PR3	1.614	0.3211	RN7SKP74	0.8121	0.2074	TRBV5-4	-2.184	0.2494
GTF2I	1.613	0.2371	RNF152P1	0.812	0.1345	LINC01694	-2.189	0.3397
BCAM	1.611	0.3046	NAA15	0.81	0.1498	ENPP7P8	-2.207	0.3715
CUEDC1	1.611	0.2702	ZBTB6	0.809	0.1899	RN7SKP245	-2.2505	0.3384
ZNF26	1.6109	0.2386	ZNF845	0.809	0.1826	MACROD2-AS1	-2.257	0.1781
KIAA0040	1.61	0.3434	GPATCH8	0.808	0.1721	LINC02109	-2.267	0.6382
TMED3	1.61	0.3277	RDH13	0.808	0.1754	TRBV6-2	-2.271	0.1708
NKAIN3	1.609	0.403	ZNF433	0.807	0.1768	LINC02043	-2.281	0.1994
TTC34	1.606	0.3504	PKNOX1	0.806	0.1406	RANBP20P	-2.281	0.5042
SNRPD1	1.604	0.3343	COQ4	0.805	0.1767	GSTA7P	-2.283	0.2076
INPP5J	1.602	0.3946	KRT8P24	0.805	0.1714	TRBV19	-2.2982	0.4221
H4-16	1.6	0.3217	VANGL1	0.805	0.1538	PTCHD1	-2.308	0.4922
MARK2P17	1.598	0.05812	ZNF140	0.805	0.1443	LINC02518	-2.319	0.2822
ITPK1	1.597	0.3434	LRRC51	0.803	0.1874	SNORA63	-2.3243	0.532
C12orf76	1.595	0.214	DNAAF4	0.802	0.1819	GLYATL1P4	-2.327	0.5643
PHC3	1.595	0.2649	MADCAM1-AS1	0.8	0.1942	TRAV8-4	-2.359	0.229
TEX26-AS1	1.595	0.3056	AMMECR1	0.798	0.1414	RN7SKP90	-2.3651	0.552
TBL1XR1-AS1	1.594	0.2991	ATG7	0.798	0.1763	LINC01767	-2.3758	0.575

KRT8P17	1.593	0.1086	FAAH	0.798	0.2147	SLCO1B7	-2.39	0.5712
MPI	1.593	0.2252	ING5	0.795	0.1754	MAPK4	-2.405	0.6192
ZNF749	1.59	0.2467	TRAPPC10	0.794	0.1694	TBX10	-2.424	0.4976
SDR42E1	1.5869	0.3228	USP38	0.794	0.1562	LINC02303	-2.4246	0.5596
MXRA7	1.58	0.3213	RBM4	0.793	0.1795	FABP7	-2.444	0.6798
GRAMD1C	1.5775	0.2042	RPL19P16	0.792	0.1588	IFIT6P	-2.444	0.3614
LMNTD2-AS1	1.577	0.409	CLN3	0.791	0.1821	SLC35F3	-2.456	0.4836
FER1L4	1.5755	0.3608	ZNF143	0.791	0.1296	EIF3CL	-2.459	0.4295
LINC01449	1.575	0.3127	LNX1-AS1	0.79	0.1964	SYNM	-2.4636	0.4715
ADGRB1	1.5721	0.3167	ZNF441	0.7891	0.1237	TRAV13-1	-2.47	0.2243
LRRC10	1.568	0.2545	GFM2	0.789	0.1547	HTR3A	-2.473	0.4895
SMIM14	1.564	0.3008	ZFP82	0.789	0.184	PTX3	-2.4752	0.5582
LINC02913	1.56254	0.3208	C9orf64	0.788	0.1276	LINC02833	-2.477	0.4637
CANT1	1.56	0.1925	ZNF764	0.787	0.1164	ECEL1P2	-2.478	0.3727
PCGF5	1.559	0.2896	COMMD3-BMI1	0.786	0.1907	CCL19	-2.4818	0.624
TTC7B	1.559	0.3862	GAPDHP27	0.785	0.1823	LINC01133	-2.552	0.6388
LINC01977	1.5571	0.4118	ZNF792	0.7831	0.1088	LRRC53	-2.557	0.6737
PAAF1	1.555	0.2273	DNAJC21	0.783	0.1835	PPIAP26	-2.575	0.4632
FDXR	1.552	0.3478	RN7SL219P	0.782	0.1994	DGCR6	-2.577	0.4839
PTMAP8	1.552	0.2581	TRIM13	0.782	0.1706	ITPRID1	-2.581	0.5484
H2BC10	1.5515	0.3089	WIPF2	0.782	0.1683	GABRE	-2.597	0.6135
DPRXP2	1.546	0.2386	ZSCAN21	0.782	0.1162	PSMC1P11	-2.612	0.2228
NOP56P1	1.545	0.1288	MIR5689HG	0.781	0.1977	LINC01634	-2.613	0.3705
LINC02417	1.544	0.2026	TNFSF13	0.781	0.2036	ID4	-2.6138	0.4291
GDPD4	1.542	0.3741	ATP6V1E1P3	0.779	0.1571	LBX1-AS1	-2.623	0.3885
LRG1	1.542	0.3905	NAIPP1	0.778	0.1979	GS1-24F4.2	-2.624	0.2784
ZNF382	1.5418	0.2164	ZNF561	0.778	0.1798	PM20D2	-2.624	0.3993
FAM24B	1.5371	0.3832	ITM2BP1	0.777	0.1695	CTD-3080P12.3	-2.645	0.2766
EIF3F	1.536	0.1802	MAT2A	0.776	0.1821	RN7SL396P	-2.6818	0.316
SAMD15	1.5337	0.3109	PLEKHH3	0.775	0.1897	MTND5P1	-2.701	0.5317
FUNDC2	1.533	0.3536	EIF4EP2	0.7743	0.1978	PICSAR	-2.701	0.4733
CFAP97D1	1.532	0.2274	OSBPL10-AS1	0.773	0.1605	TMEM74	-2.709	0.5062
IQSEC3P1	1.528	0.2462	CACYBPP3	0.771	0.135	RGL4	-2.7146	0.421
CCDC153	1.5279	0.3346	FUBP1	0.77	0.1176	SOX11	-2.746	0.6756
LEKR1	1.5267	0.2665	NPM1P5	0.77	0.1073	TUBAP9	-2.75	0.3381
ZNF396	1.5241	0.2712	ANAPC16	0.769	0.1801	LINP1	-2.756	0.443
ZNF765	1.5236	0.2095	SP1	0.769	0.1689	ANKRD20A21P	-2.797	0.2377
FAM86B3P	1.523	0.3512	CFAP36	0.768	0.2029	TRGC2	-2.8074	0.5964
KRT18P1	1.52	0.2352	RBMXL1	0.765	0.1306	NKX2-5	-2.848	0.5921
LRPAP1	1.52	0.2751	ZFPM1	0.764	0.1903	OOEPP2	-2.868	0.2181
FBP1	1.519	0.3964	BCDIN3D	0.763	0.1623	LINC02188	-2.88	0.6388
TESMIN	1.516	0.2562	ATP6V0A2	0.762	0.1881	SLC26A7	-2.918	0.472
GSTM4	1.514	0.2821	COG1	0.762	0.1541	GLYATL1P1	-2.946	0.4352
TTC41P	1.5131	0.299	DDX41	0.758	0.1326	HORMAD1	-2.956	0.7188
ZNF720	1.512	0.2422	CRADD	0.755	0.19	LINC01606	-2.987	0.6424
TMEM121	1.511	0.2845	TBC1D16	0.755	0.171	HOXA10-AS	-3.0168	0.5999
RARA	1.51	0.309	HPSE	0.7541	0.1865	CABP1-DT	-3.036	0.3192
DNAH14	1.509	0.3215	RBM33	0.754	0.1489	IQCJ-SCHIP1	-3.0457	0.2368
RFESD	1.5076	0.2619	FAN1	0.749	0.09628	BPIFA4P	-3.047	0.3854
APBB2	1.506	0.256	PPM1D	0.747	0.1569	BCL11A	-3.0893	0.6711
CTBP2	1.506	0.2545	ZNF48	0.746	0.1238	SNTG2-AS1	-3.104	0.3537
DENND5B	1.504408	0.2573	ZADH2	0.744	0.1902	SFRP1	-3.1187	0.5621
GPR135	1.503	0.3096	PRSS36	0.7425	0.1326	SNORD10	-3.138	0.4861
DCTN5	1.502	0.2937	ZNF621	0.742	0.1691	FDCSP	-3.1382	0.6227
FAM220BP	1.502	0.3412	CHM	0.736	0.1797	LINC00578	-3.2126	0.6432
MTND2P23	1.5	0.1061	TFDP1P3	0.736	0.2058	SOX10	-3.24	0.7652
HAX1P1	1.498	0.1638	ZNF563	0.7357	0.1943	LINC02027	-3.2553	0.4347
C2CD3	1.496	0.2773	PTAR1	0.735	0.1761	SLC66A1L	-3.2557	0.5874
UBE2N	1.496	0.3128	SPTY2D1	0.735	0.1002	CECR7	-3.2864	0.6324
PIWIL3	1.494	0.3365	ZDHHC3	0.735	0.1404	HOXA-AS3	-3.295	0.3819
U2AF1	1.4925	0.4167	ZNF383	0.735	0.1755	PRSS41	-3.3206	0.4064
ZNF233	1.4907	0.1879	ZFP62	0.734	0.129	PVRIG2P	-3.4025	0.4659
RGL2	1.488	0.2639	ZNF160	0.734	0.1669	MOG	-3.417	0.6996
WDR45BP1	1.4869	0.3219	C19orf48	0.732	0.1588	KCNG1	-3.4797	0.6834
RPL12P17	1.485	0.07501	CYB5D1	0.732	0.148	IGHV7-4-1	-3.5266	0.7888
RPL13	1.485	0.3266	VPS39	0.732	0.1625	PART1	-3.5773	0.5802
DDX60L	1.48	0.3004	HMGB3P5	0.731	0.0968	UGT2B24P	-3.692	0.5412
TMEM95	1.48	0.2258	NUDT9	0.729	0.1352	TRGC1	-3.773	0.5822
CYHR1	1.473	0.2538	ZNF480	0.729	0.1204	CALML5	-3.8542	0.9656
KRT18P10	1.472	0.2989	LINC00943	0.727	0.1812	TMEM256-PLSCR3	-3.866	0.4859
H1-12P	1.4715	0.2647	GTF2IP12	0.725	0.1433	IGLV3-27	-3.89	0.3859
SYS1	1.471	0.3421	ZNF71	0.725	0.1537	CHRM3-AS2	-3.937	0.3168
IFITM2	1.469	0.313	IMPDH2	0.723	0.1227	PLAAT1	-3.9834	0.7
HID1	1.468	0.3413	ZNF627	0.722	0.1514	ATP13A5	-4.408	0.5618
PEX2	1.466	0.3164	RN7SL606P	0.721	0.1455	STAC2	-4.41	0.9771
IDH3A	1.464	0.2187	NINJ1	0.719	0.1695	GLYATL2	-4.448	0.8349

YTHDF2P1	1.464	0.245	NRXN2-AS1	0.717	0.1339	GGTLC2	-4.62	0.7368
NOL4L	1.463	0.227	HNRNPA3	0.716	0.1366	EN1	-4.713	0.994
SYTL2	1.455	0.3117	SERINC2	0.716	0.1902	C3orf85	-4.961	0.7396
TM2D1	1.455	0.2254	AP3M1	0.715	0.1084	RNU2-2P	-5.2426	0.4116
TTC39A-AS1	1.4523	0.3977	DDX39B	0.714	0.1624	CFC1	-6.434	1.073
SYNGAP1	1.452	0.262						
