# Supplementary material for: ERα/PR crosstalk is altered in the context of the ERα Y537S mutation and contributes to endocrine therapy-resistant tumor proliferation
Source: NPJ Breast Cancer. 2023 Nov 30;9:96. doi: 10.1038/s41523-023-00601-7 (PMC10689488; doi:10.1038/s41523-023-00601-7)
Supplement: Supplementary file 2 — Related Manuscript File [file 41523_2023_601_MOESM2_ESM.pdf]

nature portfolio

Corresponding author(s): Geoffrey L Greene

Last updated by author(s): Sep 18, 2023

Reporting Summary

Nature Portfolio wishes to improve the reproducibility of the work that we publish. This form provides structure for consistency and transparency in reporting. For further information on Nature Portfolio policies, see our Editorial Policies and the Editorial Policy Checklist.

Statistics

For all statistical analyses, confirm that the following items are present in the figure legend, table legend, main text, or Methods section.

n/a	Confirmed	
☐	☒	The exact sample size (*n*) for each experimental group/condition, given as a discrete number and unit of measurement
☐	☒	A statement on whether measurements were taken from distinct samples or whether the same sample was measured repeatedly
☐	☒	The statistical test(s) used AND whether they are one- or two-sided<br>*Only common tests should be described solely by name; describe more complex techniques in the Methods section.*
☐	☒	A description of all covariates tested
☐	☒	A description of any assumptions or corrections, such as tests of normality and adjustment for multiple comparisons
☐	☒	A full description of the statistical parameters including central tendency (e.g. means) or other basic estimates (e.g. regression coefficient) AND variation (e.g. standard deviation) or associated estimates of uncertainty (e.g. confidence intervals)
☐	☒	For null hypothesis testing, the test statistic (e.g. *F*, *t*, *r*) with confidence intervals, effect sizes, degrees of freedom and *P* value noted<br>*Give P values as exact values whenever suitable.*
☒	☐	For Bayesian analysis, information on the choice of priors and Markov chain Monte Carlo settings
☐	☒	For hierarchical and complex designs, identification of the appropriate level for tests and full reporting of outcomes
☒	☐	Estimates of effect sizes (e.g. Cohen's *d*, Pearson's *r*), indicating how they were calculated

Our web collection on statistics for biologists contains articles on many of the points above.

Software and code

Policy information about availability of computer code

Data collection	No software was used.
Data analysis	RNA-seq data were uploaded to the Galaxy platform and analyzed using the public server at usegalaxy.org. Sequencing files were mapped to the hg19 human reference genome using Bowtie2 and read counts per gene were generated from the aligned sequences using HTSeq-Count. DESeq2 was used to determine differentially expressed genes between each cell variant and between each treatment.

For manuscripts utilizing custom algorithms or software that are central to the research but not yet described in published literature, software must be made available to editors and reviewers. We strongly encourage code deposition in a community repository (e.g. GitHub). See the Nature Portfolio guidelines for submitting code & software for further information.

Data

Policy information about availability of data

All manuscripts must include a data availability statement. This statement should provide the following information, where applicable:

- Accession codes, unique identifiers, or web links for publicly available datasets
- A description of any restrictions on data availability
- For clinical datasets or third party data, please ensure that the statement adheres to our policy

The datasets used during the current study are largely available in the supplemental files, and RNA-Seq data is publicly available through NCBI Gene Expression Omnibus (GEO accession #: GSE243454). Any data not included are available from the corresponding author upon reasonable request. Data used to identify

April 2023

potential overlapping chromatin binding sites of ERα and PR are available in Khushi, M., C.L. Clarke, and J.D. Graham, Bioinformatic analysis of cis-regulatory interactions between progesterone and estrogen receptors in breast cancer. PeerJ, 2014. https://doi.org/10.7717/peerj.654.

Research involving human participants, their data, or biological material

Policy information about studies with human participants or human data. See also policy information about sex, gender (identity/presentation), and sexual orientation and race, ethnicity and racism.

Reporting on sex and gender	The data generated in this study did not involve human participants. Human tumor data from the MET500 and POG570 datasets were used in gene expression analyses. These data only included female patients with breast cancer.
Reporting on race, ethnicity, or other socially relevant groupings	n/a
Population characteristics	Data included in our analyses (from the publicly available MET500 and POG570 datasets) were characterized as either estrogen receptor wild-type or estrogen receptor Y537S.
Recruitment	n/a
Ethics oversight	n/a

Note that full information on the approval of the study protocol must also be provided in the manuscript.

Field-specific reporting

Please select the one below that is the best fit for your research. If you are not sure, read the appropriate sections before making your selection.

☒ Life sciences ☐ Behavioural & social sciences ☐ Ecological, evolutionary & environmental sciences

For a reference copy of the document with all sections, see nature.com/documents/nr-reporting-summary-flat.pdf

Life sciences study design

All studies must disclose on these points even when the disclosure is negative.

Sample size	Three biological replicates (experimental repeats with new samples) were completed for each experiment in this study. Where applicable, such as with plated cell growth experiments, a minimum of three technical replicates (repeated plating with the same samples) were used as well.
Data exclusions	No data were excluded from this study.
Replication	As mentioned above, three biological replicates were completed for each experiment. All replicates are accounted for in the data presented in this manuscript.
Randomization	Randomization was not relevant to our study.
Blinding	Blinding was not relevant to the study because all data were objectively quantifiable and knowledge of samples was critical.

Reporting for specific materials, systems and methods

We require information from authors about some types of materials, experimental systems and methods used in many studies. Here, indicate whether each material, system or method listed is relevant to your study. If you are not sure if a list item applies to your research, read the appropriate section before selecting a response.

Materials & experimental systems

n/a	Involved in the study	
☐	☒	Antibodies
☐	☒	Eukaryotic cell lines
☒	☐	Palaeontology and archaeology
☒	☐	Animals and other organisms
☒	☐	Clinical data
☒	☐	Dual use research of concern
☒	☐	Plants

Methods

n/a	Involved in the study	
☒	☐	ChIP-seq
☒	☐	Flow cytometry
☒	☐	MRI-based neuroimaging

Antibodies

Antibodies used	1:10 D8Q2J rabbit monoclonal antibody (Cell Signaling #8757) was used for the detection of PR-A and PR-B in proximity ligation assays (PLA). 1:10 F10 mouse monoclonal antibody (Santa Cruz Biotechnology #sc-8002) was used for the detection of ERα in PLA. F10 and anti-IRS1 mouse monoclonal antibody (Santa Cruz Biotechnology #sc-8038) were used for immunoblot detection of ERα and IRS1, respectively, both at 1:10 using the Bio-Techne ProteinSimple WES platform. 1:100 AC-15 mouse monoclonal antibody (Santa Cruz Biotechnology #sc-69879) was used for the detection of β-actin as a loading control in immunoblot detection. KD68 rat monoclonal antibody (originally generated by Greene et al. [1988] and produced and purified by the University of Chicago Flow Cytometry Core) was used for immunoblot detection of PR, as well as for chromatin immunoprecipitation (ChIP) to immunoprecipitate chromatin to which PR-A or PR-B was bound. The ERα C-terminal antibody from Epicypher (#13-2012) was used for ERα immunoprecipitation in ChIP. Normal rabbit IgG and normal rat IgG (Santa Cruz Biotechnology #sc-2027 and #sc-2026, respectively) were used as negative control antibodies for Epicypher ERα C-terminal and KD68, respectively.
Validation	Anti-PR D8Q2J - highly cited and validated by Cell Signaling Technology for use in immunoblotting<br>Anti-ERα F10 - highly cited and validated by Santa Cruz Biotechnology for use in immunoblotting, confirmed specificity through siESR1 knockdown and immunoblot detection in this study (Supplemental Figure 7)<br>Anti-IRS1 E-12 -highly cited and validated by Santa Cruz Biotechnology for use in immunoblotting, confirmed specificity through siIRS1 knockdown and immunoblot detection in this study (Supplemental Figure 8)<br>Anti-b-actin AC15 - highly cited and validated by Santa Cruz Biotechnology for use in immunoblotting<br>Anti-PR KD68 - originally purified and validated by Greene et al. (1988), available from the authors upon request<br>Anti-ERα C-terminal - validated in MCF7 cells for multiple applications by EpiCypher, including immunoprecipitation and CUT&RUN<br>Normal rabbit IgG and normal rat IgG - highly cited, retrievable from Santa Cruz Biotechnology

Eukaryotic cell lines

Policy information about cell lines and Sex and Gender in Research

Cell line source(s)	MCF7 and T47D breast cancer cell lines were derived from female patients and originally obtained from the ATCC. MCF7 parent cells (MCF7 ERα WT) and MCF7 ERα Y537S-het were generated and gifted by Ben Ho Park, originally at Johns Hopkins University and now at Vanderbilt University. MCF7 ERα Y537S-hom cells were generated and gifted by Sarat Chandarlapaty at Memorial Sloan Kettering Cancer Center. T47D parent cells (T47D ERα WT) and T47D ERα Y537S-het cells were generated and gifted by Steffi Oesterreich at the University of Pittsburgh. T47D ERα Y537S-hom were generated by David Shapiro at the University of Illinois at Urbana-Champaign originally and were gifted from Carol Lange at the University of Minnesota.
Authentication	Cell lines were validated for ERα receptor status (WT, Y537S-heterozygous, or Y537S-homozygous) through next generation sequencing (NGS) completed by the University of Illinois at Chicago Genome Research Core.
Mycoplasma contamination	All cell lines were tested for mycoplasma after thawing fresh cells and prior to beginning experimentation. Testing was completed using the MycoAlert Mycoplasma Detection Kit (Lonza Bioscience #LT07-318).
Commonly misidentified lines<br>(See ICLAC register)	n/a
